# Novel trifluoromethylpyridine piperazine derivatives as potential plant activators

**DOI:** 10.3389/fpls.2022.1086057

**Published:** 2022-11-28

**Authors:** Wei Zhang, Shengxin Guo, Ya Wang, Hong Tu, Lijiao Yu, Zhichao Zhao, Zhenchao Wang, Jian Wu

**Affiliations:** ^1^ State Key Laboratory Breeding Base of Green Pesticide and Agricultural Bioengineering, Ministry of Education, Guizhou University, Guiyang, China; ^2^ Key Laboratory of Green Pesticide and Agricultural Bioengineering, Ministry of Education, Guizhou University, Guiyang, China

**Keywords:** piperazine, synthesis, anti-viral activity, mechanisms, qRT-PCR analysis, plant activator

## Abstract

Plant virus diseases seriously affect crop yield, especially tobacco mosaic virus (TMV) and cucumber mosaic virus (CMV). The development of plant immune activators has been an important direction in the innovation of new pesticides. Therefore, we designed and synthesized a series of trifluoromethyl pyridine piperazine derivatives (A1-A27), and explored the action mechanism of active compound. The antiviral activity test showed that compounds A1, A2, A3, A9, A10, A16, A17 and A21 possessed higher activities than commercialized ningnanmycin. Particularly, the in vivo antiviral activity indicated that compound A16 showed the most potent protective activity toward TMV (EC50 = 18.4 μg/mL) and CMV (EC50 = 347.8 μg/mL), compared to ningnanmycin (50.2 μg /mL for TMV, 359.6 μg/mL for CMV). The activities of defense enzyme, label -free proteomic and qRT-PCR analysis showed that compound A16 could enhance the defensive enzyme activities of superoxide dismutase (SOD),polyphenol oxidase (PPO) and phenylalanine ammonialyase (PAL), and activate the phenylpropanoid biosynthesis pathway to strenthen the antiviral activities of tobacco. This study provides reliable support for the development of new antiviral pesticides and potential antiviral mechanism.

## 1 Introduction

Plant virus diseases seriously affect crop production, causing global economic losses of up to $60 billion annually (Bos, 2000; Zhao et al., 2017). Taking tobacco mosaic virus (TMV) and cucumber mosaic virus (CMV) as examples, they can host hundreds of crops (Wei et al., 2019; Yuan et al., 2022). TMV is one of the oldest known plant viruses, and once infected with TMV, plants develop viral diseases with symptoms including stunting, leaf mosaic and shedding (Xiang et al., 2019). CMV could infect many crops in addition to cucumbers, and plants infected by CMV generally exhibited dwarfing, leaf curls, necrosis, and even plant death and often bring about huge economic losses (Palukaitis et al., 1992; Garcia-Arenal et al., 2000). Therefore, it is urgent to develop efficient and stable pesticide.

**Figure 1:**
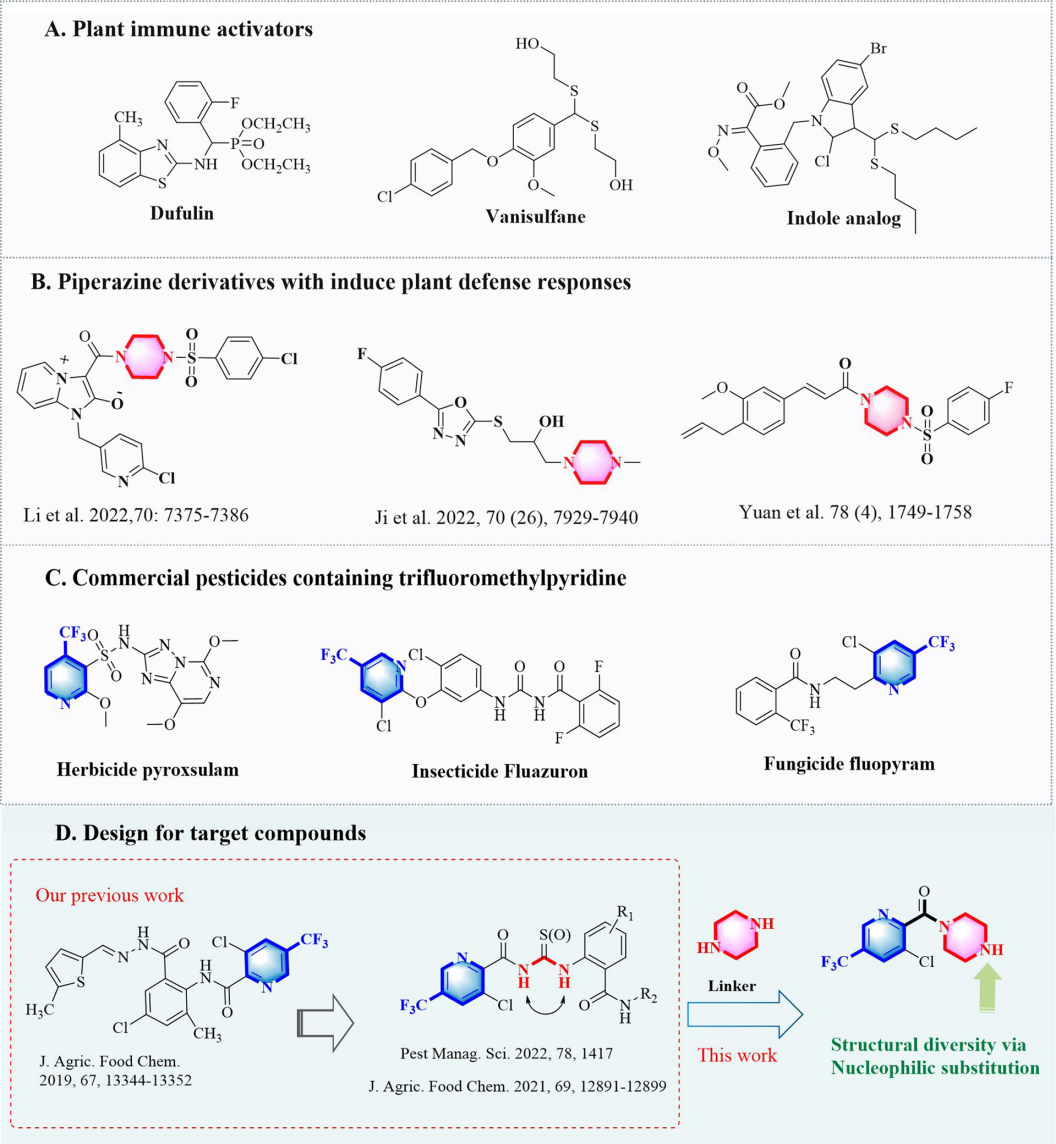
Design ideas for target compounds. **(A)** "Plant immune activators". **(B)** " Piperazine derivatives with induce plant defense responses". **(C)** "Commercial pesticides containing trifluoromethylpyridine". **(D)** "Design for target compounds".

In the long-term evolution and the game with the pests, plants have already formed a relatively complete immune system (Aljbory et al., 2018; Jones et al., 2006; Zhang et al., 2019). Plants could resist the foreign invasion by releasing phytoalexin, ethylene, salicylic acid and other substances (Hu et al., 2017; Klessig et al., 2018; He et al., 2021; Guo et al., 2021). Studies have shown that exogenous substances could elicit the defense responses of plants to give the SAR (systemic acquired resistance) for resisting the infection of microorganisms. More than 10 exogenous substances have been developed as activators of endogenous substances for controlling plant virus diseases (Xu et al., 2006; Qiu, 2014). The development of plant immune activators has been an important direction in the innovation of new pesticides (Nakashita, 2021; Stephens et al., 2022). As an activator of salicylic acid pathway, dufulin (**Figure 1A**) has been successfully developed for controlling the viruses in rice and vegetable in China (Chen et al., 2012). More recently, vanisulfane (Shi et al., 2018; Shi et al., 2022) and an indole analog (Wei et al., 2019) were discovered as activators by switching on the abscisic acid (ABA) signal pathway and malate dehydrogenase (MDH) pathway in plants, respectively.

As an important and special organic base with a symmetrical diamine structure, piperazine often serves as a bridge connecting the different active groups to expand the diversity of structures (Elliott, 2011; Zhang et al., 2021; Garg et al., 2021; Acharya et al., 2021). Compounds containing the structure of piperazine with different biological activities (Stoilkova et al., 2014; Han et al., 2020; Fang et al., 2022), are widely used as the potential plant activators for striving against the plant viruses. Li and co-workers (Li et al., 2022) (**Figure 1B**) found that the piperazine derivatives with a moiety of imidazo could activate the glycolysis/gluconeogenesis pathway in tobacco to mitigate the infection of potato virus Y. Ji et al. (Ji et al., 2022) synthesized some piperazine derivatives *via* installing an oxadiazole sulfide, which were confirmed to inhibit the systemic spread of TMV from adjacent tissues of tobacco plants and the biosynthesis process of TMV. Additionally, increasing expression of genes in photosystem II and parts of the cytochrome b6/f complex could uniquely express and activate photosynthesis by the piperazine derivatives with the substitution of sulfonyl and unsaturated phenylpropionic acid (Yuan et al., 2022).

Moreover, the unique electronic effects and fat-soluble penetration effects of trifluoromethyl pyridine could greatly affect the conformation and metabolism of compounds (Zheng et al., 2022). It has become one of the main active structures of many commercial pesticides, such as the herbicide pyroxsulam (Nugent et al., 2015), the insecticide fluazuron (Cai et al., 2010; Chen et al., 2019), and the fungicide fluopyram (Xie et al., 2014), *etc* (**Figure 1C**). Our previous work (**Figure 1D**, left), revealed the trifluoromethyl pyridine derivatives showed significant anti-viral activity (Wang et al., 2019a;; Guo et al., 2021; Xu et al., 2022). Herein, in order to discover antiviral molecule with trifluoromethyl pyridine, we sought to make a cyclization for the replacement of the thiourea/urea (Guo et al., 2021; Xu et al., 2022) by combining the trifluoromethyl pyridine with piperazine, and installing the substitutions *via* nucleophilic substitution reaction at opposite end of piperazine, which may result in trifluoromethyl pyridine piperazine derivatives with good antiviral activity (**Figure 1D**, right). Consequently, 27 novel trifluoromethyl pyridine piperazine derivatives were synthesized. Bioassays against TMV and CMV indicated that some of the compounds showed excellent antiviral activities. Further studies indicated that the compounds with excellent protective activity could induce the activities of superoxide dismutase (SOD), polyphenol oxidase (PPO) and phenylalanine ammonialyase (PAL). The phenylpropanoid biosynthetic pathway could also be triggered by the active compounds, thereby the systemic acquired resistance (SAR) could be enhanced by the trifluoromethyl pyridine piperazine derivatives. This type of piperazine derivative could be regarded as the potential plant activators for controlling plant viruses.

## 2 Materials and methods

### 2.1 Chemicals and instruments

All reagents and solvents were obtained from TCI (Tokyo Chemical Industrial Development Co., Ltd) and used without further purification. Newly synthesized intermediates and piperazine derivatives were characterized using an AVANCE III HD 400 MHz nuclear magnetic resonance (NMR, ^1^H, ^13^C, and ^19^F) spectrometer (Bruker Corp., Fallanden, Switzerland) and using an XT-4 binocular microscope (Beijing Tech Instrument Co., China) to determine the melting points. Thermo Scientific, St. Louis, MO, U.S.A. was used for high-resolution mass spectrometry (HR-MS). The qRT-PCR analysis was performed using a PCR thermal cycler (Bio-RAD, USA). Defense enzyme detection (Suzhou Comin Biotechnology Co., Ltd., China).

### 2.2 Synthetic of compounds A1 − A27

**Figure 2:**
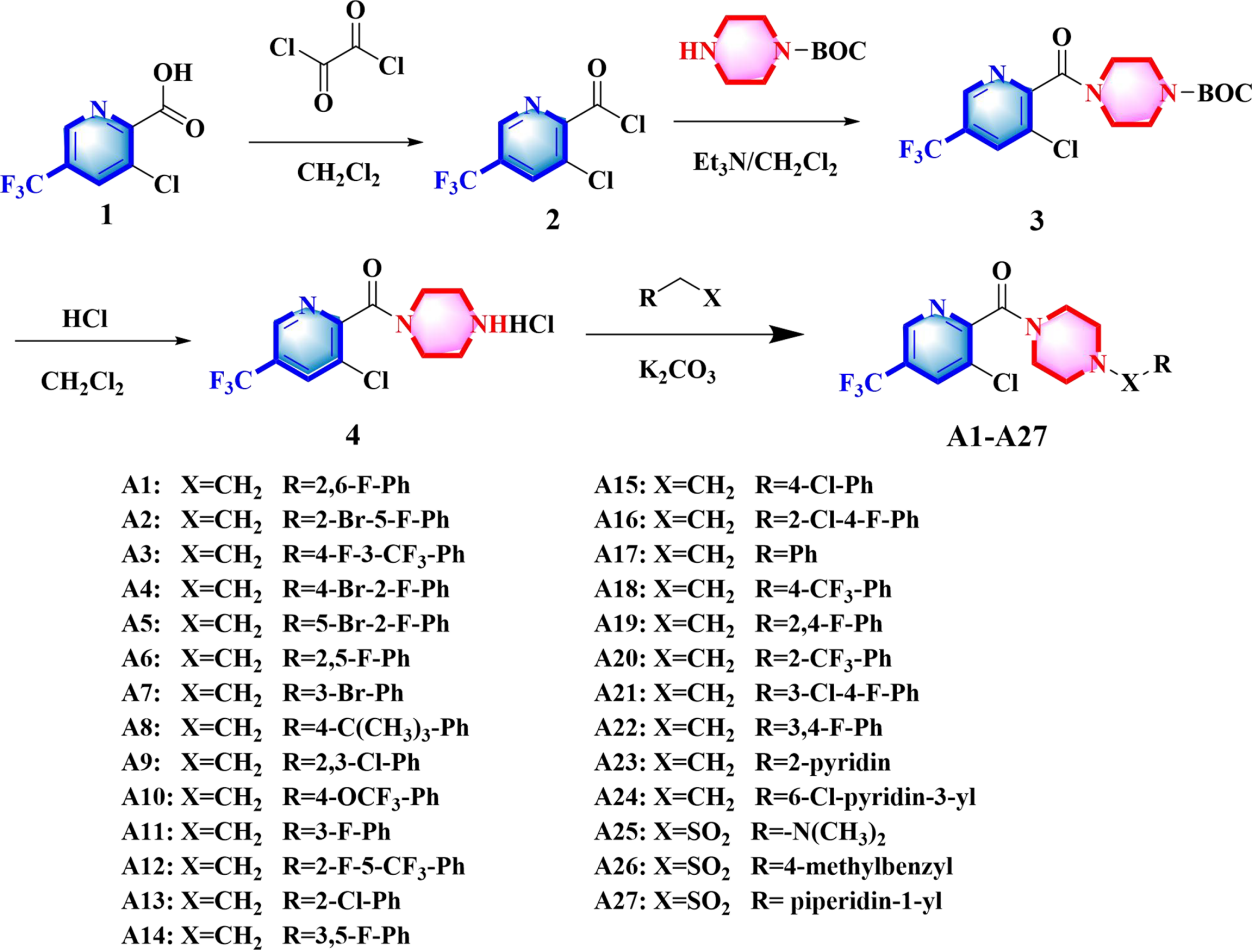
Synthesis of compounds **A1−A27**.

The synthetic route for **A1 − A27** was depicted as **Figure 2**. More details for the protocols and the spectral information (^1^H, ^13^C, ^19^F NMR, and HR-MS) of synthesized compounds were given in the Supporting Information.

### 2.3 Antiviral activity assay

For antiviral activity assays, the *nicotiana tabacum* L. and *chenopodium amaranticolor* plants were used as experimental plants. The anti-TMV activity of compounds A1−A27 were preliminarily screened at 500 μg/mL by the half-leaf method (Song et al., 2005; Wang et al., 2019b). Then the compounds with outstanding activity were tested for their anti-TMV activities at 500, 250, 125, 62.5 and 31.25 μg/mL with NNM as the positive control agent. After the lesions recording 2−3 days later, half maximal effective concentrations (EC_50_) value were calculated. Finally, for target compounds with outstanding anti-TMV activities (Gooding and Hebert, 1967), anti-CMV activities were evaluated on *chenopodium amaranticolor* by using the same half-leaf method (Luo et al., 2013).

#### 2.3.1 Curative activity of target compounds

The silicon carbide was sprinkled evenly on the leaves, then the virus was dipped in a brush and gently rubbed on the leaves. After inoculation with the virus, the leaves were rinsed with clean water half an hour later. When the leaves were dry, the target compounds solutions were applied on the left side of the leaves, and the other side was smeared with 1% tween 80 solvent as a control. After 2 − 3 days, the number of spots was counted, and each experiment was repeated 3 times.

#### 2.3.2 Protective activity of target compounds

Different from the experimental procedure for curative activity, the target compounds solution was smeared on the left side of the leaf, the 1% tween 80 solvent was smeared on the other side as a control. Then 24 h later, the virus was inoculated on leaves, and the leaves were rinsed with clean water after half an hour. The number of spots followed 2 − 3 days appearing was counted, and each experiment was repeated 3 times.

#### 2.3.3 Inactivation activity of target compounds

Firstly, the silicon carbide was sprinkled evenly on the leaves. After the target compounds mixing with the virus solution for 30 min, the mixture was then smeared on the left side of the leaves, and the other side of the leaves were coated with a mixture of solvent and virus as a control. After half an hour of inoculation, the leaves were washed with water. The number of the appeared spots was counted after 2 − 3 days. Each experiment was repeated 3 times.

### 2.4 Defense enzyme activity assays

K326 tobacco at the six-leaf stage was selected and treated with compound **A16**, NNM (positive control) and CK (negative control), respectively. Four treatment modes were applied in this work, such as CK, “CK + TMV”, “A16 + TMV” and “NNM + TMV”. After 24h spraying of compound A16 and NNM (500 μg/mL) evenly, the TMV was inoculated on the leaves. Then the tobacco leaves were collected on the 1^st^, 3^rd^, 5^th^, and 7^th^ days, and stored at -80 °C. Finally, the activities of SOD, PPO and PAL were determined with a defense enzyme detection kit (Suzhou Comin Biotechnology Co., Ltd., China), and all experiments were repeated 3 times.

### 2.5 Label-free proteomic analysis

When the defense enzyme activities were measured, it were found that the activities were significantly different on the third day after infection with the TMV virus, so K326 tobacco on the third day was used as a sample for proteomic analysis. Both protein extraction and protein identification were entrusted to APTBIO (Shanghai China). GO functional annotation of all differentially expressed proteins (DEPs) were analyzed by Blast2Go software. (https://www.blast2go.com/). Analysis and annotation of proteins through the Kyoto Encyclopedia of Genes and Genomes (KEGG) pathway database were produced (http://www.genome.jp/kegg/pathway.html).

### 2.6 RNA extraction and qRT-PCR analysis

The total RNA from the tobacco sample was extracted by using the Trizol kit (Vazyme, China). RNA reverse transcription was explored by using a cDNA kit (Vazyme) with *β*-actin as the endogenous control. qRT-PCR experiments and calculations used in this work were followed by the literature (Livak and Schmittgen, 2001). The design and synthesis of primers were entrusted to Sangon Bioengineering (Shanghai) Co., Ltd.

## 3 Results and discussion

### 3.1 Anti-TMV activity

The primary anti-TMV activity (including curative, protective, and inactivation activities) of A1−A27 are shown in **Table 1**. The EC_50_ values of some active compounds are shown in **Table 2**. The following is a preliminary structure-activity relationship analysis.

**Table 1:** Activity of compounds A1–A27 on TMV.

Compounds	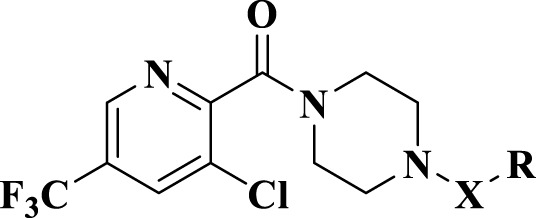		Curativeactivity (%)	Protective activity (%)	Inactivation activity (%)
	X	R			
**A1**	CH_2_	2,6-F-Ph	64.3±4.9	66.0±5.6	49.1±4.5
**A2**	CH_2_	2-Br-5-F-Ph	66.1±3.5	70.3±5.0	67.3±1.8
**A3**	CH_2_	4-F-3-CF_3_-Ph	59.4±3.5	79.1±4.6	67.0±4.5
**A4**	CH_2_	4-Br-2-F-Ph	41.9±5.0	64.9±0.6	81.1±4.1
**A5**	CH_2_	5-Br-2-F-Ph	57.5±5.0	68.2±3.6	65.6±4.6
**A6**	CH_2_	2,5-F-Ph	32.5±5.5	64.1±5.2	79.3±5.0
**A7**	CH_2_	3-Br-Ph	39.7±2.4	44.0±3.6	83.3±3.8
**A8**	CH_2_	4-C(CH_3_)_3_-Ph	56.6±1.3	56.1±4.9	84.9±2.8
**A9**	CH_2_	2,3-Cl-Ph	59.0±1.8	48.5±1.4	88.4±4.3
**A10**	CH_2_	4-OCF_3_-Ph	43.4±2.8	46.0±5.6	93.1±3.4
**A11**	CH_2_	3-F-Ph	58.3±2.9	59.1±3.1	78.2±4.0
**A12**	CH_2_	2-F-5-CF_3_-Ph	26.5±3.4	52.5±3.8	61.1±4.4
**A13**	CH_2_	2-Cl-Ph	39.3±5.0	43.6±1.1	67.8±1.4
**A14**	CH_2_	3,5-F-Ph	34.8±4.4	50.2±5.0	83.6±4.9
**A15**	CH_2_	4-Cl-Ph	60.7±4.9	66.0±5.1	81.2±4.8
**A16**	CH_2_	2-Cl-4-F-Ph	62.8±3.9	87.0±3.7	78.8±3.5
**A17**	CH_2_	Ph	68.6±3.6	60.3±4.3	76.2±3.7
**A18**	CH_2_	4-CF_3_-Ph	57.3±2.7	57.0±4.7	64.0±4.0
**A19**	CH_2_	2,4-F-Ph	53.8±2.6	46.1±3.8	74.8±2.5
**A20**	CH_2_	2-CF_3_-Ph	43.8±4.5	71.3±4.9	59.4±3.2
**A21**	CH_2_	3-Cl-4-F-Ph	47.3±3.1	76.7±1.9	71.5±5.1
**A22**	CH_2_	3,4-F-Ph	40.4±6.0	33.1±1.5	77.8±2.7
**A23**	CH_2_	2-pyridin	51.2±5.0	64.8±2.3	72.9±4.3
**A24**	CH_2_	6-Cl-pyridin-3-yl	37.3±6.3	60.0±2.2	67.8±1.9
**A25**	SO_2_	-N(CH_3_)_2_	56.4±4.5	68.5±5.0	78.3±4.9
**A26**	SO_2_	4-methylbenzyl	29.1±1.5	56.4±0.7	74.5±2.9
**A27**	SO_2_	piperidin-1-yl	32.3±2.3	63.4±6.7	69.3±3.3
**NNM**			56.6±3.2	74.5±4.9	91.4±2.9

**Table 2:** EC_50_ of active title compounds against TMV.

Compounds	Curative effect		Protective effect				Inactivation effect		
	Regression equation	R^2^	EC_50_ (μg/mL)	Regression equation	R^2^	EC_50_ (μg/mL)	Regression equation	R^2^	EC_50_ (μg/mL)
**A1**	y=0.58x+3.72	0.92	155.3	/	/	/	/	/	/
**A2**	y=0.73x+3.52	0.95	112.3	/	/	/	/	/	/
**A3**	/	/	/	y=0.39x+4.49	0.92	20.2	/	/	/
**A9**	/	/	/	/	/	/	y=0.81x+3.66	0.97	43.1
**A10**	/	/	/	/	/	/	y=1.15x+3.00	0.96	54.5
**A16**	y=0.72x+3.54	0.98	107.8	y=0.59x+4.25	0.91	18.4	/	/	/
**A17**	y=0.79x+3.47	0.97	86.1	/	/	/	/	/	/
**A21**	/	/	/	y=0.62x+3.91	0.95	57.2	/	/	/
**NNM**	y=0.72x+3.48	0.99	131.7	y=0.62x+3.93	0.94	50.2	y=1.56x+2.53	0.90	38.0

“/” indicates no test activity.

The curative activities of most compounds against TMV were higher than that of NNM at 500 μg/mL, and compound A17 (R = benzene) had the highest curative activity (68.6%, EC_50_ = 86.1 μg/mL), which was higher than that of NNM (56.6%, EC_50_ = 131.7 μg/mL). When X = CH_2_, R contains a benzene ring, the curative activity was generally more prominent. However, with the addition of other groups to the benzene ring of R, the activity decreased slightly. For example, the curative activities of R = 2,6-difluorobenzyl (A1), R = 2-bromo-5-fluorobenzyl (A2), R = 4-fluoro-3-(trifluoromethyl)benzyl (A3), R = 5-bromo-2- fluorobenzyl (A5), R = 2,3-dichlorobenzyl (A9), R = 3-fluorobenzyl (A11), R = 4-chlorobenzyl (A15), R = 2-chloro-4-fluorobenzyl (A16) were 64.3, 66.1, 59.4, 57.5, 59.0, 58.3, 60.7 and 62.8%, respectively, and the activities were higher than that of NNM. In particular, the EC_50_ = 112.3 μg/mL of compound A2 and the EC_50_ = 107.8 μg/mL of A16 were lower than those of NNM. When X = SO_2_, R = *N, N*-dimethy (A25) had a curative activity of 56.4%, which was comparable to that of NNM, but the overall curative activity is not as good as when X is -CH_2_ and R is a benzene ring.

Some compounds had good protective activities against TMV at 500 μg/mL, especially the protective activities of compounds A3 (X = CH_2_, R = 4-fluoro-3-(trifluoromethyl)benzyl)) and A16 (X = CH_2_, R = 2-chloro-4- fluorobenzyl)) are 79.1%, EC_50_ = 20.2 μg/mL and 87.0%, EC_50_ = 18.4 μg/mL, which were higher than that of NNM (74.5%, EC_50_ = 50.2 μg/mL). The activity of A21 was 76.7% higher than that of NNM at 500 μg/mL.

Compound A10 (X = CH_2_, R = 4-(trifluoromethoxy)benzyl) had the highest inactivation activity (93.1%, EC_50_ = 54.5 μg/mL), which was higher than that of NNM (91.4%, EC_50_ = 38.0 μg/mL). The activity of compound A9 (X = CH_2_, R = 2,3-dichlorobenzyl) with EC_50_ 43.1 μg/mL was similar to that of NNM.

### 3.2 Anti-CMV activity

Because of the favorable anti-TMV activities of these compounds, the curative, protective and inactivation activities against CMV were also evaluated. As shown in **Table 3**, some compounds showed good curative and protective activities. The curative activities of compounds A1 and A3 at 500 μg/mL were 64.1 and 61.0%, respectively, which were higher than NNM (59.0%). Meanwhile, A3 and A16 showed a remarkable protective effect on CMV, with protective values of 58.0 and 47.8%, respectively, which were superior to that of NNM (44.2%). The protective activity of compound A16 (EC_50_ = 347.8 μg/mL) was higher than that of NNM (EC_50_ = 359.64 μg/mL). The inactivation activities of compounds A9 and A10 were 62.4% and 63.3%, respectively, lower than that of NNM (89.6%).

**Table 3:** Activity of compounds A1-A27 on CMV.

Compounds	Curative activity (%)	EC_50_ of curative activity (μg/mL)	Protective activity (%)	EC_50_ of protective activity (μg/mL)	Inactivating activity (%)
**A1**	64.1 ± 4.1	242.3	40.7 ± 4.8	/	/
**A2**	46.7 ± 2.1	/	42.2 ± 4.9	/	/
**A3**	61.0 ± 3.8	256.5	58.0 ± 4.6	399.5	/
**A9**	49.6 ± 2.2	/	29.2 ± 3.9	/	62.4 ± 4.3
**A10**	47.8 ± 2.2	/	37.3 ± 1.4	/	63.3 ± 2.9
**A16**	49.0 ± 3.8	/	47.8 ± 2.9	347.8	/
**A17**	54.2 ± 4.2	/	36.3 ± 3.3	/	/
**A21**	48.2 ± 1.8	/	40.0 ± 4.0	/	/
NNM	59.0 ± 3.0	234.7	44.2 ± 1.2	359.6	89.6 ± 4.1

“/” indicates no test activity.

### 3.3 Defensive enzyme activity assay

**Figure 3:**
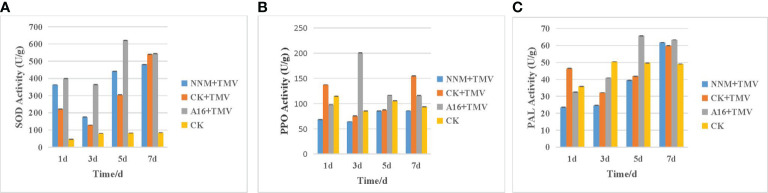
Effects of compound **A16** on SOD **(A)**, PPO **(B)** and PAL **(C)** activities in tobacco leaves. Ningnanmycin is NNM. Vertical bars refer to mean ± SD.

Among the protective activities, compound A16 explored the best and most stable characteristic, so the preliminary antiviral mechanism of A16 was further investigated. SOD, PAL and PPO are protective enzymes in plants, and their strength is directly related to the ability of plants to resist diseases. Therefore, the effects of compound A16 on the above enzymes in tobacco were examined (**Figure 3**). PAL (Zhang et al., 2018; Li et al., 2019) induced by the biosynthetic pathway of secondary metabolites such as lignin and phytoalexin, activates the system to acquire resistance, strengthens the cell wall, and inhibits pathogen infection. In defense enzyme assays, “A16 + TMV” presented an earlier increase and later decrease trend both in PPO and PAL. The PAL activity of “A16 + TMV” reached the peak on the fifth day (65 units/g), which was 1.67, 1.57 and 1.32 times that of “NNM + TMV”, “CK + TMV” and CK, respectively. SOD is a metal antioxidant enzyme widely existing in natural organisms (Pan et al., 2020) that defends against the damage of superoxide free radicals to cells, and maintains the normal physiological metabolism and biochemical reactions of cells in the organism. After infecting tobacco with TMV, the SOD activity of the “A16 + TMV” was higher than that of “CK + TMV” and “NNM + TMV”. At the same time, the SOD activity of “A16 + TMV” group reached the peak on the fifth day (622 units/g), which was 1.41, 2.03 and 7.58 times of “NNM + TMV”, “CK + TMV” and CK, respectively. PPO is a copper-containing oxidoreductase (Boeckx et al., 2015), which catalyzes the formation of quinones from phenolic substances during the growth and development of crops, and has a defensive effect on insect pests and pathogenic bacteria. The PPO activity of “A16 + TMV” reached a peak (200 units/g) on the third day, which were 3.13, 2.63 and 2.35 times that of “NNM + TMV”, “CK + TMV” and CK, respectively. These results indicated that compound A16 could enhance the activities of SOD, PPO and PAL defense enzymes after TMV infection, thereby improving the disease resistance of plants.

### 3.4 Label-free proteomic analysis

**Figure 4:**
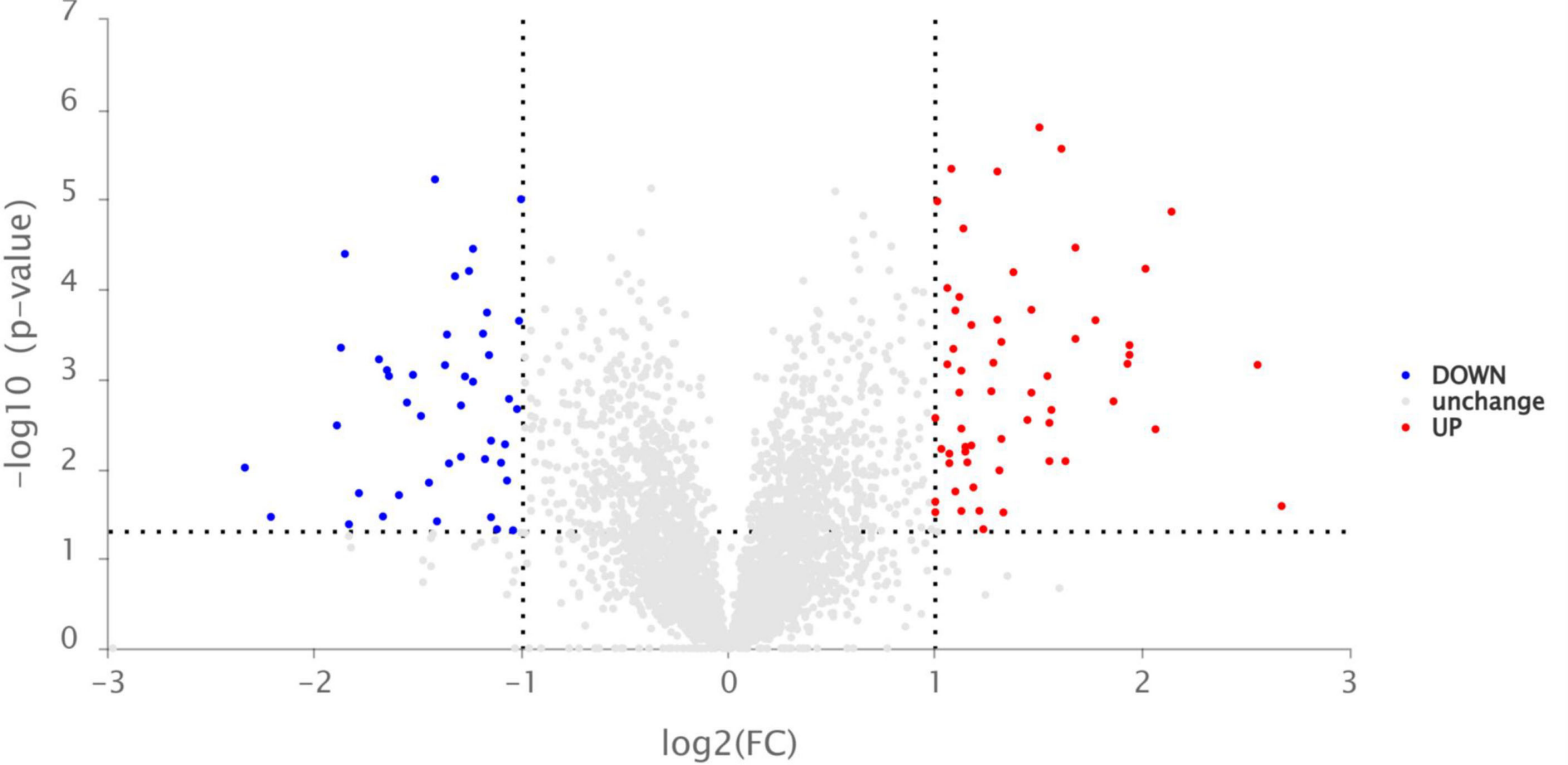
Volcano plot of the relative protein abundance changes between the “A16 + TMV” and “CK + TMV” treatments.

Given the promising determination of defense enzyme activity, the expression levels of SOD, PPO, and PAL in the tobacco leaves treated by compound A16 explored significant presentation on the third day. Hence, in order to investigate the anti-TMV mechanism of A16 on tobacco, Label-Free quantitative proteomics were carried out. Different protein expressions (DEPs) of tobacco in the “CK+TMV” and “**A16** + TMV” groups on the third day were analyzed. As indicated in **Figure 4**, a total of 107 DEPs were screened and identified, of which 63 proteins were upregulated (red dots; Fold Change, FC > 2, P value < 0.05), 44 proteins were downregulated (blue dots; Fold Change, FC < 0.50, P value < 0.05).

### 3.5 GO analysis

**Figure 5:**
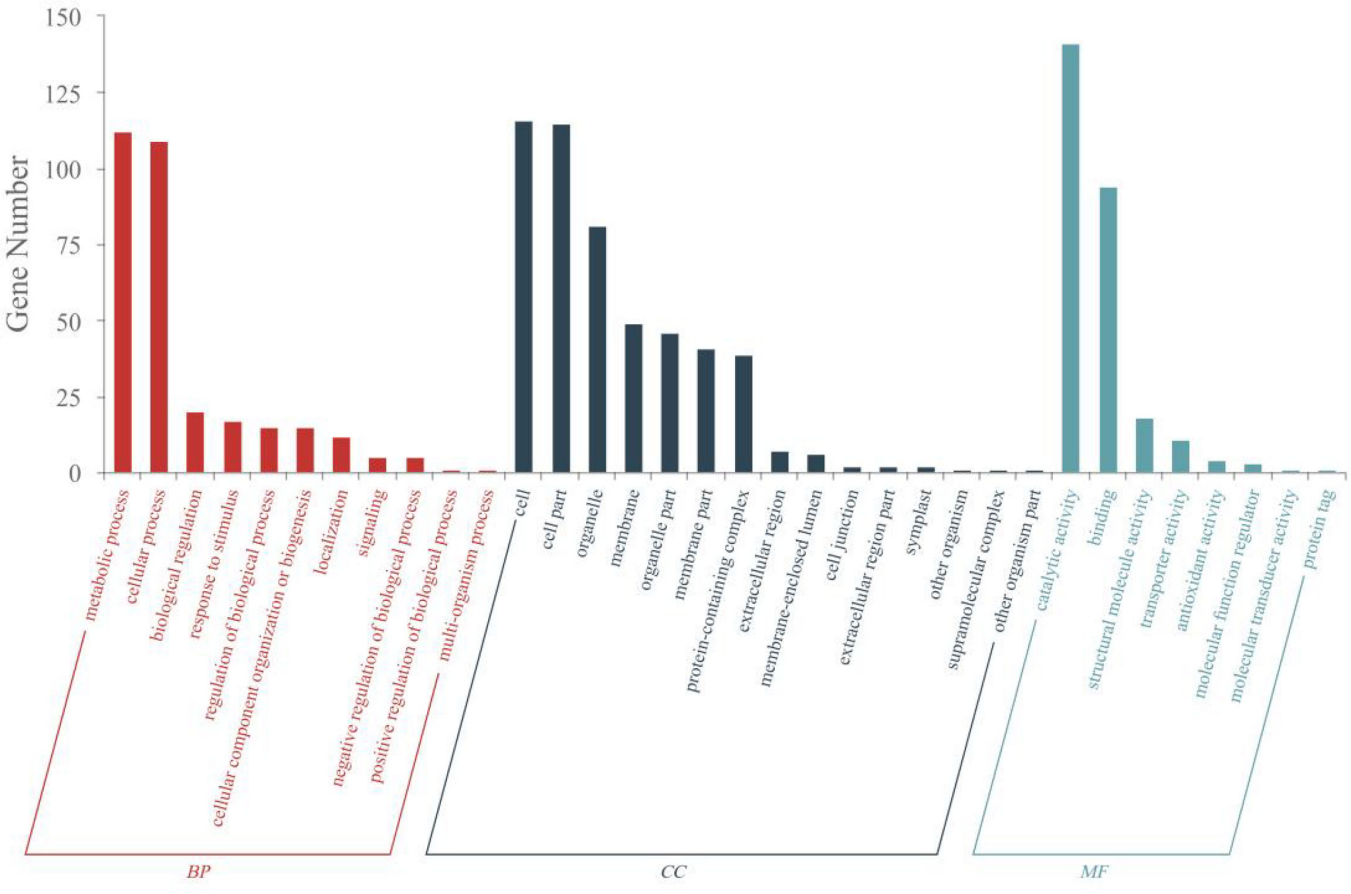
GO annotation statistics of DEPs (P value <0.05) of “CK+TMV” and “A16+TMV”.

DEPs induced by **A16** in tobacco was further interpreted by using gene ontology (GO) annotation (p value < 0.05). The functional information of DEPs is divided into three categories: biological process (BP), molecular function (MF), and cellular component (CC). BP were involved in metabolic processes, cellular processes, biological regulation, response to stimulus, regulation of biological process, cellular component organization or biogenesis, localization, signaling, negative regulation of biological process, positive regulation of biological process, multi-organism process. MF were mainly involved in catalytic activity, binding, structural molecule activity, transporter activity, antioxidant activity, molecular function regulator, molecular transducer activity, protein tag. CC included cell, cell part, organelle, membrane, organelle part, membrane part, protein-containing complex, extracellular region, membrane-enclosed lumen, cell junction, extracellular region part, symplast, other organism, supramolecular complex, other organism part. These results suggested that compound **A16** could alter plant physiology in many ways, some of which were related to plant resistance to viruses (**Figure 5**).

### 3.6 KEGG analysis

**Figure 6:**
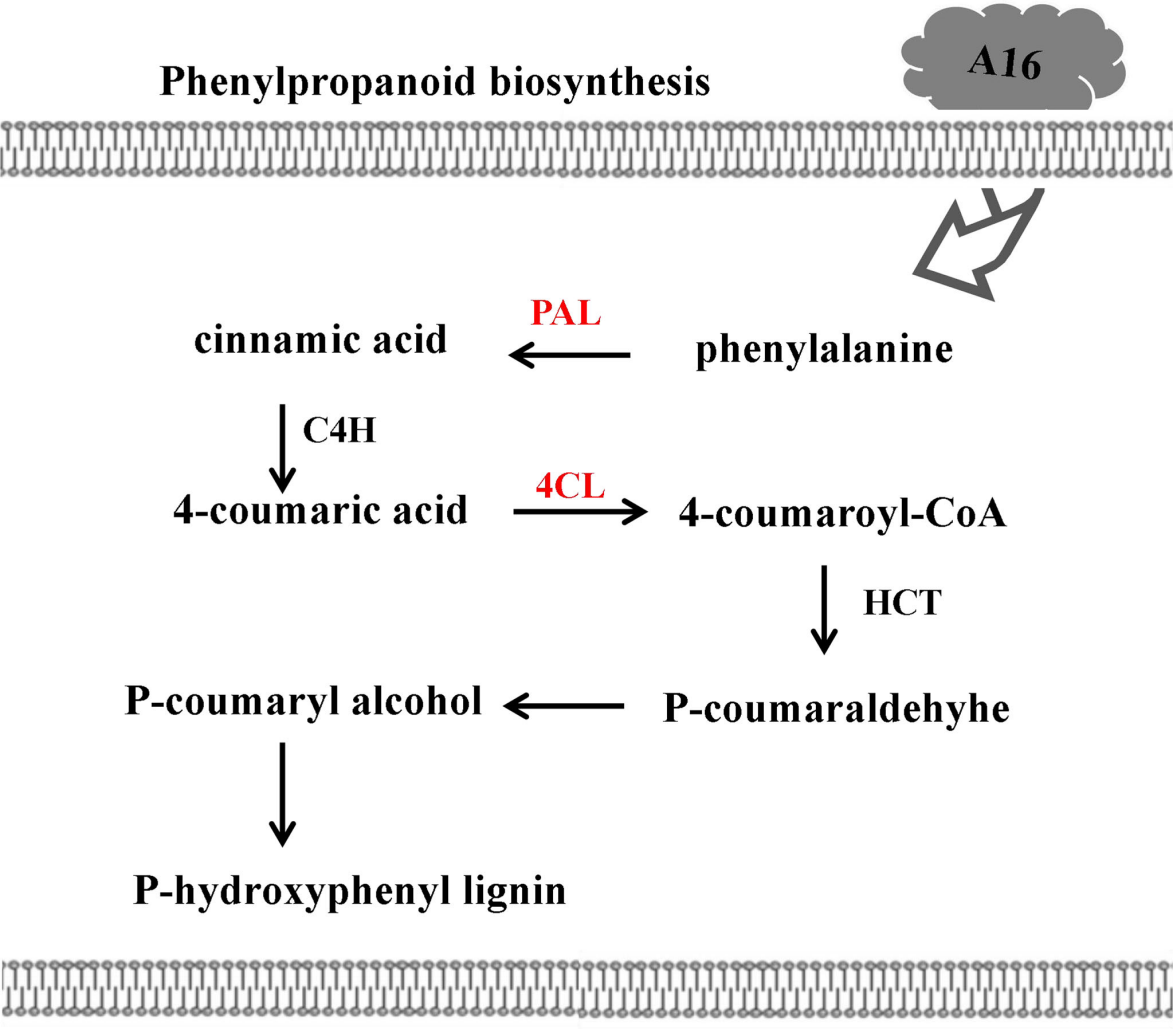
Phenylpropanoid biosynthesis pathway in tobacco response to A16. The red color represents upaccumulated proteins in this pathway. PAL, phenylalanine ammonialyaze; C4H, cinnamate acid 4-hydroxylase; 4CL, 4-coumarate-CoA; HCT, hydroxycinnamoyl transferase.

Through the database KEGG, the potential biological pathways (P value < 0.05) involved in DEPs of compound **A16** were investigated (**Figure 6** and **Table 4**). Seven DEPs related to the phenylpropanoid biosynthesis pathway including four upregulated proteins (A0A1S3X5R5_TOBAC, 7.60-fold; Q70G33_TOBAC, 1.06-fold; A0A1S3ZTJ1_TOBAC, 1.36-fold; A1XEL3_TOBAC, 1.34-fold) and three downregulated proteins (A0A1S3Y048_TOBAC, 0.27-fold; Q9XIV9_TOBAC, 0.0008-fold; A0A1S4CAV2_TOBAC, 0.15-fold). Phenylpropanoid biosynthesis is one of the important secondary metabolic pathways in plants, and the upregulated protein A0A1S3X5R5 (PAL) is the first key enzyme for the production of phenolic compounds in plants. Cinnamate acid 4-hydroxylase (C4H) and 4-coumarate-COA ligase (A0A1S3ZTJ1, 4CL) are key enzymes for the synthesis of phenolic compounds associated with disease resistance. When plants are infected by a virus, the activity of PAL increases rapidly (Reinold and Hahlbrock, 1996), promotes the synthesis of phenolic compounds (Xia and Gao, 2009), inhibits virus infection and proliferation, and induces plants resistance (French et al., 1991; Li et al., 2021). At the same time, PAL could induce 4CL enzyme activity to promote lignin synthesis and improve plants disease resistance (Lawton et al., 1980; Subramaniam et al., 1993). The phenylpropanoid biosynthesis pathway was involved in plants disease resistance and immunity. On the one hand, due to 4CL promotes the synthesis of lignin, it thickens the cell wall and forms a physical barrier that prevents viruses from invading cells. On the other hand, the produced phenolic metabolites can further synthesize phytoalexin, inhibit viruses, and comprehensively regulate the disease resistance and defense capabilities of plants (Mauch-Mani and Slusarenko, 1996). Q70G33_TOBAC, A0A1S3ZTJ1_TOBAC and A1XEL3_TOBAC all belong to phenols, which indicates that these upregulated DEPs play an important role in plants resistance. These results showed that the phenylpropanoid biosynthesis pathway may be the main reason for the protective mechanism of the compound A16 against TMV.

**Table 4:** DEPs involved in the phenylpropanoid biosynthesis pathway.

Protein ID	Protein names	Gene names	Organism	Sig/Specific
A0A1S3X5R5_TOBAC	phenylalanine ammonialyase	LOC107761482	nicotiana tabacum	up
Q70G33_TOBAC	hydroxycinnamoyl CoA quinate transferase	N/A	nicotiana tabacum	up
A0A1S3ZTJ1_TOBAC	4-coumarate–CoA ligase	LOC107790326	nicotiana tabacum	up
A1XEL3_TOBAC	CYP73A47v2	N/A	nicotiana tabacum	up
A0A1S3Y048_TOBAC	peroxidase (EC 1.11.17)	LOC107770624	nicotiana tabacum	down
Q9XIV9_TOBAC	peroxidase (EC 1.11.17)	LOC107825099	nicotiana tabacum	down
A0A1S4CAV2_TOBAC	peroxidase (EC 1.11.17)	LOC107817021	nicotiana tabacum	down

**Figure 7:**
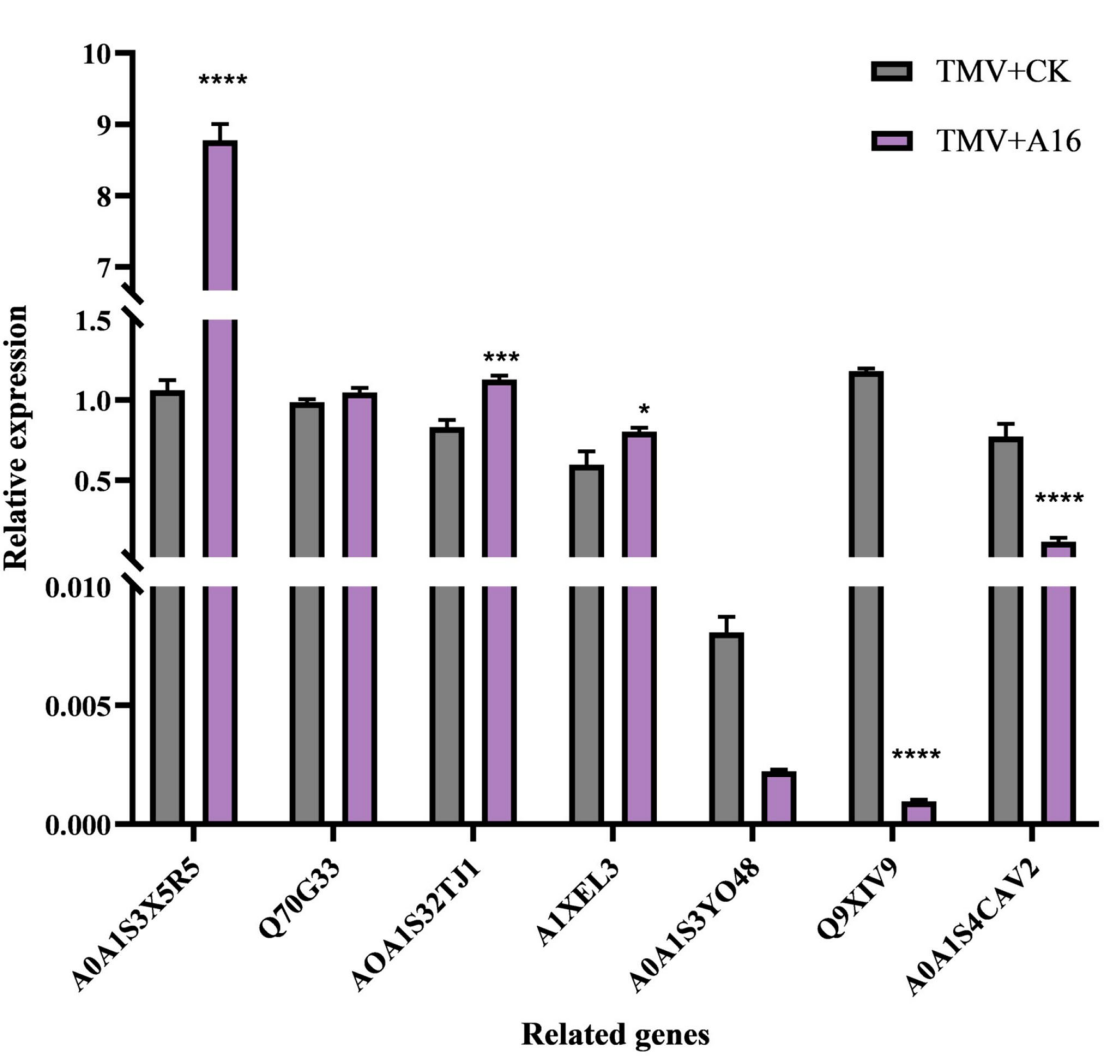
Gene expression analysis of the related genes of the phenylpropanoid biosynthesis by qRT-PCR. (β-actin gene served as the internal control). Vertical bars refer to mean ± SD. ****", "***" and "*" represent significance at the 0.0001, 0.001 and 0.05 level, respectively.

Gene Expression Analysis. To enhance the understanding of the above proteomic conclusions, the confirmation of DEPs expression was accomplished by a qRT-PCR method. As listed in **Table 5**, the tested seven genes include A0A1S3X5R5_TOBAC, Q70G33_TOBAC, A0A1S3ZTJ1_TOBAC, A1XEL3_ TOBAC, A0A1S3Y048_TOBAC, Q9XIV9_TOBAC and A0A1S4CAV2_ TOBAC, with *β*-actin as the endogenous control. As shown in **Figure 7**, after the tobacco plant was triggered by compound A16, the upregulated gene A0A1S3X5R5_TOBAC was strongly activated with a 7-fold change. The other three upregulated genes: Q70G33_TOBAC, A0A1S3ZTJ1_TOBAC and A1XEL3_TOBAC also changed at least 1-fold. The downregulated genes A0A1S3Y048_TOBAC, Q9XIV9_TOBAC and A0A1S4CAV2_TOBAC were also strongly inhibited. These results are consistent with the proteomic results, further confirming that compound A16 can modulate the phenylpropanoid biosynthesis pathway and it can be used as a promising lead compound for controlling plant viruses with inducer function.

**Table 5:** Primer sequences of qRT-PCR.

Protein ID reverse	Forward primer	Reverse primer
A0A1S3X5R5_TOBAC	ATGCTCTCCGAACATCTCCACAATG	AGTTGCCACCATGTAACGCCTTG
Q70G33_TOBAC	CACTGATGGTAGGTCTAGGCTTTGC	TTGCCATAGGTGTGCCTGTGAAC
A0A1S3ZTJ1_TOBAC	CAGAGCGGTTCAAGAGCAGGTTC	AGGCGACCAGCAGATCCATACC
A1XEL3_TOBAC	GGAAGAAACCCGAAGAGTTCAGACC	GCTCCTCCTACCAACGCCAAAC
A0A1S3Y048_TOBAC	CCATTGCTGCTAGGGACTCTGTTG	GACTGAGGACGACTGTGGTGTTG
Q9XIV9_TOBAC	GGGACAACAATTTGGCACCACTTG	TCACAATCGAATCGGCAGATCCAC
A0A1S4CAV2_TOBAC	TTGCTGCTAGAGAAGGCGTTGTG	TTGGTGCTGGGATTTGGGTGTTG
β-actin	AGGGTTTGCTGGAGATGATG	CGGGTTAAGAGGTGCTTCAG

In summary, we synthesized novel trifluoromethyl piperazine derivatives and evaluated their TMV and CMV activities. The biological activities showed that some of the compounds showed good to excellent antiviral activities on TMV and CMV. Particularly, the protective activity of A16 was significantly higher than that of NNM, which could enhance the defense enzymes activities of SOD, PPO, and PAL. Label-free quantitative proteome bioinformatics and the qRT-PCR confirmation studies showed that the phenylpropanoid biosynthesis pathway was induced by compound A16 against TMV. Our findings suggested that compound A16 is a promising new lead compound for antiviral molecule. Further modifications and derivations are under way in our laboratory.

## Data availability statement

The original contributions presented in the study are included in the article/supplementary materials. Further inquiries can be directed to the corresponding authors.

## Author contributions

WZ, SG, JW conceived and designed the experiments. Synthesis and bio-assay were carried out by WZ, YW, and LY; WZ, ZZ and JW analyzed the data; WZ wrote the original draft; HT, ZZ, ZW and JW reviewed and edited the manuscript. All authors contributed to the article and approved the submitted version.

## Funding

The financial supports from NSFC (National Natural Science Foundation of China) (Nos. 32072445, 21867004), the Program of Introducing Talents to Chinese Universities (D20023), Frontiers Science Center for Asymmetric Synthesis and Medicinal Molecules, Department of Education, Guizhou Province [Qianjiaohe KY (2020)004], and Graduate Research Fund in Guizhou Province YJSKYJJ[2021]038, and the Specific Research Fund of The Innovation Platform for Academicians of Hainan Province (SQ2020PTZ0009).

## Acknowledgments

The assistance for the proteomics test from APTBIO (Shanghai China) was also appreciated.

## Conflict of interest

The authors declare that the research was conducted in the absence of any commercial or financial relationships that could be construed as a potential conflict of interest.

## Publisher’s note

All claims expressed in this article are solely those of the authors and do not necessarily represent those of their affiliated organizations, or those of the publisher, the editors and the reviewers. Any product that may be evaluated in this article, or claim that may be made by its manufacturer, is not guaranteed or endorsed by the publisher.
