# Supplementary material for: Novel trifluoromethylpyridine piperazine derivatives as potential plant activators
Source: Front Plant Sci. 2022 Nov 28;13:1086057. doi: 10.3389/fpls.2022.1086057 (PMC9742420; doi:10.3389/fpls.2022.1086057)

Supporting Information

Novel Trifluoromethylpyridine Piperazine Derivatives as Potential Plant Activators

Wei Zhang[†], Shengxin Guo[†], Ya Wang[†], Hong Tu[†], Lijiao Yu[†], Zhichao Zhao[†], Zhenchao Wang*[, †], and Jian Wu*[, †]

***CORRESPONDING AUTHOR FOOTNOTE**

*Authors to whom correspondence should be addressed;

E-mails: jwu6@gzu.edu.cn. zcwang@gzu.edu.cn

[†]State Key Laboratory Breeding Base of Green Pesticide and Agricultural Bioengineering, Key Laboratory of Green Pesticide and Agricultural Bioengineering, Ministry of Education, Guizhou University, Huaxi District, Guiyang 550025, China

Contents

1. Synthetic of Compound A1 − A27 3
2. General remarks 3
3. ^{1}H NMR, ^{13}C NMR , ^{19} F NMR and HRMS spectra for the target compounds (A1-A27). 3
4. NMR Spectra and HRMS spectra of Products 18

1. Synthetic of Compound A1 − A27

1.0 g of starting material **1** (1.0 mmol) was added to a round bottom flask and dissolved with 5 mL of dichloromethane (DCM). Then 675.1 mg of oxalyl chloride (1.2 mmol) and three drops of *N,N*-dimethylformamide were added and stirred at room temperature (rt) for 3 h, to give **2**. The solution of intermediate **2** (1.0 g, 1.0 mmol) in DCM was dropwise added into the mixed solution of 1-tert-butoxycarbonylpiperazine (827.1 mg, 1 mmol) with triethylamine (1.1 mL, 2.0 mmol) in DCM (5 mL). The resulting mixture was stirred at room temperature and monitored by TLC. After the reaction is completed, the resulting mixture was poured into 5% NaOH aqueous solution to extract DCM. Then, the organic phase was separated, combined and dried with anhydrous magnesium sulfate, and intermediate **3** was obtained followed by filtering and desolubilization, subsequently . Intermediate **4** was then synthesized by reaction of intermediate **3** (1.0 mmol) in DCM with dropwise adding hydrochloric acid (12.0 mmol) to the solution. After the mixture was treated by stirring at rt and monitoring by TLC, intermediate **4** was obtained by desolvation. Anhydrous potassium carbonate (5.0 mmol) and 5 mL acetonitrile were added to intermediate **4** (1 mmol), then the different acyl group/sulfonyl active groups (1.2 mmol) were added after stirring at rt for 5 min. Afterward the reaction was detected by TLC, and the target compounds (**A1**−**A27**) was isolated by column chromatography

2. General remarks

The following abbreviations were used to designate chemical shift multiplicities, s = singlet, d = doublet, t = triplet, q = quartet, m = multiplet, and brs = broad singlet. High resolution mass spectrometer (HRMS) data was conducted using a Thermo Scientific Q Exactive (Thermo, USA). Reaction progress was monitored with thin-layer chromatography (TLC) on silica gel GF_{254}. Column chromatographic purification was carried out using silica gel 200 to 300 mesh. All solvents and reagents were of analytical reagent grade and were dried and purified in accordance with standard procedures before use.

3. ^{1}H NMR, ^{13}C NMR , ^{19} F NMR and HRMS spectra for the target compounds (A1-A27).

(3-chloro-5-(trifluoromethyl)pyridin-2-yl)(4-(2,6-difluorobenzyl)piperazin-1-yl)methanone (A1)

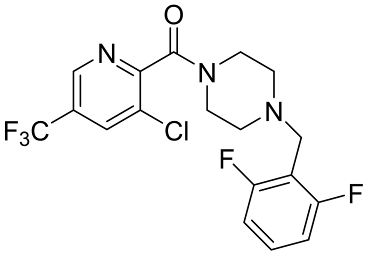

Yield 95.4%; Yellow solid; m. p. 92 - 94°C.

<u>^{1}H NMR</u> (400 MHz, DMSO) δ 8.98 (dd, *J* = 1.8, 0.8 Hz, 1H,

Ar-H), 8.63 (d, *J* = 1.3 Hz, 1H, Ar-H), 7.42 (td, *J* = 8.3, 4.2 Hz, 1H, Ph-H), 7.15 – 7.07 (m, 2H, Ph-H), 3.66 (d, *J* = 4.8 Hz, H, -CH_2-), 3.63 (s, 2H, Piperazine-H), 3.16 – 3.09 (m, 2H, Piperazine-H), 2.51 – 2.44 (m, 2H, Piperazine-H), 2.38 – 2.29 (m, 2H, Piperazine-H).

^{13}C NMR (100 MHz, DMSO) δ 163.5 (s), 161.8 (dd, *J* = 247.0, 8.4 Hz), 156.4(d, *J* = 1.0 Hz), 145.2 (q, *J* = 4.0 Hz), 136.1 (q, *J* = 3.4 Hz), 132.3 – 129.4 (m), 128.4 (s), 126.9 (d, *J* = 33.4 Hz), 123.0 (d, *J* = 273.3 Hz), 112.6 (t, *J* = 19.8 Hz), 112.0 (d, *J* = 6.2 Hz), 111.9 (d, *J* = 6.0 Hz), 52.6 (s), 52.0 (s), 48.5 (s), 46.2 (s), 41.4 (s).

^{19}F NMR (376 MHz, DMSO) δ -60.87 , -113.73.

HRMS(ESI): Calculated for $C_{18}H_{16}ON_3ClF_5[M+H]^+$: 420.0897, found: 420.0891.

(4-(2-bromo-5-fluorobenzyl)piperazin-1-yl)(3-chloro-5-(trifluoromethyl)pyridin-2-yl)methanone (A2)

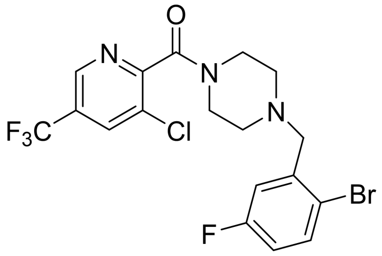

Yield 24.4%; Yellow solid; m. p. 106 - 108°C.

^{1}H NMR (400 MHz, DMSO) δ 9.00 (d, *J* = 1.0 Hz, 1H, Ar-H), 8.65 (d, *J* = 1.3 Hz, 1H, Ar-H), 7.59 – 7.56 (m, 1H, Ph-H), 7.54 – 7.51 (m, 1H, Ph-H), 7.26 (d, *J* = 2.6 Hz, 1H, Ph-H), 3.69 (s, 2H, -CH_2-), 3.57 (s, 2H, Piperazine-H), 3.19 – 3.06 (m, 2H, Piperazine-H), 2.53 – 2.45 (m, 2H, Piperazine-H), 2.40 (s, 2H, Piperazine-H).

^{13}C NMR (100 MHz, DMSO) δ 163.5 (s), 159.1(dd, *J* = 119.9, 1.9 Hz), 157.3 (d, *J* = 2.0 Hz), 156.4 (s), 156.1 (d, *J* = 2.1 Hz), 145.2 (q, *J* = 3.9 Hz), 136.1 (q, *J* = 3.3 Hz), 128.4(s), 126.9 (d, *J* = 33.1 Hz), 123.0 (q, *J* = 273.3 Hz), 117.9 (d, *J* = 28.4 Hz), 117.2 (dd, *J* = 25.3, 8.9 Hz), 116.1 (dd, *J* = 22.6, 10.1 Hz), 54.4 (s), 52.9 (s), 52.2 (s), 46.2 (s), 41.4 (s).

^{19}F NMR (376 MHz, DMSO) δ -60.86 , -113.50.

HRMS(ESI): Calculated for $C_{18}H_{16}ON_3BrClF_4[M+H]^+$: 480.0095, found: 480.0085.

(3-chloro-5-(trifluoromethyl)pyridin-2-yl)(4-(4-fluoro-3-(trifluoromethyl)benzyl)piperazin-1-yl)methanone (A3)

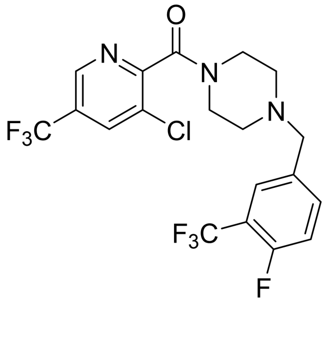

Yield 95.0%; White solid; m. p. 102 - 104°C.

^{1}H NMR (400 MHz, DMSO) δ 8.99 (s, 1H, Ar-H), 8.64 (s, 1H, Ar-H), 7.70 (d, *J* = 6.9 Hz, 2H, Ph-H), 7.48 (t, *J* = 9.9 Hz, 1H,

Ph-H), 3.68 (s, 2H, -CH_2-), 3.59 (s, 2H, Piperazine-H), 3.15 (d, J = 4.3 Hz, 2H, Piperazine-H), 2.48 – 2.45 (m, 2H, Piperazine-H), 2.34 (d, J = 4.2 Hz, 2H, Piperazine-H).

^{13}C NMR (100 MHz, DMSO) δ 163.6 (s), 156.8 (d, J = 249.4 Hz), 156.4 (s), 146.3 – 14.26 (m), 136.1 (t, J = 7.5 Hz), 135.9 – 135.3 (m), 128.4 (s), 127.9 – 127.3 (m), 123.0 (q, J = 273.4 Hz), 123.2 (d, J = 271.5 Hz),117.5 (d, J = 20.1 Hz), 100.0 (s), 60.4 (s), 53.0 (s), 52.3 (s), 46.3 (s), 41.5 (s).

^{19}F NMR(376 MHz, DMSO) δ -60.01 , -60.90 , -118.47 .

HRMS(ESI): Calculated for $C_{19}H_{16}ON_3ClF_7[M+H]^+$: 470.0865, found: 470.0860.

(4-(4-bromo-2-fluorobenzyl)piperazin-1-yl)(3-chloro-5-(trifluoromethyl)pyridin-2-yl)methanone (A4)

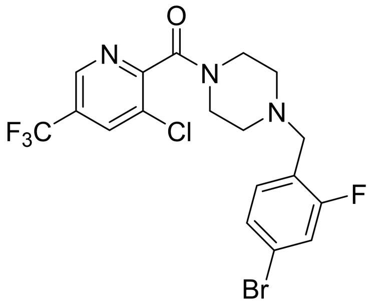

Yield 62.4%; Yellow solid; m. p. 92 - 94°C.

^{1}H NMR (400 MHz, DMSO) δ 8.99 (dd, J = 1.8, 0.8 Hz, 1H, Ar-H), 8.65 – 8.60 (m, 1H, Ar-H), 7.54 – 7.49 (m, 1H, Ph-H), 7.39 (d, J = 6.6 Hz, 2H, Ph-H), 3.70 – 3.62 (m, 2H, -CH_2-), 3.55 (s, 2H, Piperazine-2H), 3.19 – 3.10 (m, 2H, Piperazine-2H), 2.47 (t, J = 4.9 Hz, 2H, Piperazine-2H), 2.39 – 2.27 (m, 2H, Piperazine-2H).

^{13}C NMR (100 MHz, DMSO) δ 163.5 (s), 161.1 (d, J = 249.7 Hz), 156.4 (s), 145.2 (q, J = 3.7 Hz), 136.9 – 135.2 (m), 133.7 (d, J = 5.2 Hz), 128.4 (s), 127.9 (d, J = 3.5 Hz), 126.9 (d,J=33.2Hz),124.2 (d, J = 14.8 Hz), 123.0 (q, J = 273.3 Hz), 121.0 (d, J = 9.8 Hz), 119.1 (d, J = 25.7 Hz), 54.3 (s), 52.9 (s), 52.2 (s), 46.2 (s), 41.5 (s).

^{19}F NMR(376 MHz, DMSO) δ -60.87 , -114.75 .

HRMS(ESI): Calculated for $C_{18}H_{16}BrClF_4[M+H]^+$: 480.0096, found: 480.0093.

(4-(5-bromo-2-fluorobenzyl)piperazin-1-yl)(3-chloro-5-(trifluoromethyl)pyridin-2-yl)methanone (A5)

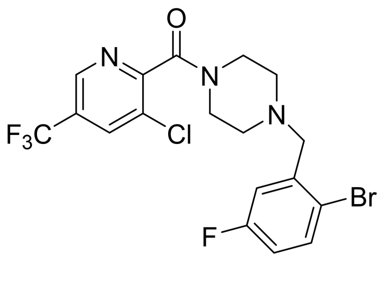

Yield 62.4%; White solid; m. p. 168 - 169°C.

^{1}H NMR (400 MHz, DMSO) δ 9.00 (d, J = 1.0 Hz, 1H, Ar-H), 8.65 (d, J = 1.3 Hz, 1H, Ar-H), 7.65 (dd, J = 8.8, 5.4 Hz, 1H, Ph-H), 7.37 (dd, J = 9.8, 3.2 Hz, 1H, Ph-H), 7.12 (td, J = 8.5,

3.2 Hz, 1H, Ph-H), 3.74 – 3.67 (m, 2H, -CH_2-), 3.58 (s, 2H, Piperazine-H), 3.26 – 3.07 (m, 2H, Piperazine-H), 2.60 – 2.52 (m, 2H, Piperazine-H), 2.46 – 2.38 (m, 2H, Piperazine-H).

^{13}C NMR (100 MHz, DMSO) δ 163.5 (s), 161.9 (d, *J* = 244.3 Hz), 156.4 (s), 145.2 (d, *J* = 3.9 Hz), 140.1 (d, *J* = 7.3 Hz), 136.1 (d, *J* = 3.5 Hz), 134.6 (d, *J* = 8.2 Hz), 128.4 (s), 126.9 (d, *J* = 33.2 Hz), 123.0 (q, *J* = 273.3 Hz), 118.5 (d, *J* = 2.8 Hz), 117.8 (d, *J* = 23.5 Hz), 116.6 (d, *J* = 22.6 Hz), 60.9 (s), 53.2 (s), 52.6 (s), 46.3 (s), 41.5 (s).

^{19}F NMR (376 MHz, DMSO) δ -60.84, -114.64 .

HRMS(ESI): Calculated for $C_{18}H_{16}ON_3BrClF_4[M+H]^+$: 480.0096, found: 480.0095.

(3-chloro-5-(trifluoromethyl)pyridin-2-yl)(4-(2,5-difluorobenzyl)piperazin-1-yl)methanone (A6)

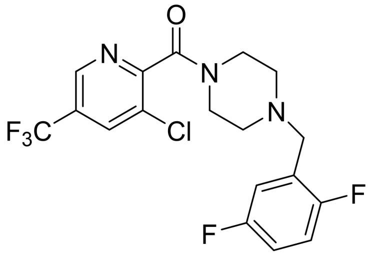

Yield 65.0%; Yellow solid; m. p. 107 - 109°C.

^{1}H NMR (400 MHz, DMSO) δ 8.99 (dd, *J* = 1.8, 0.8 Hz, 1H, Ar-H), 8.64 (d, *J* = 1.3 Hz, 1H, Ar-H), 7.30 – 7.14 (m, 3H, Ph-H), 3.69 (s, 2H, -CH_2-), 3.58 (s, 2H, Piperazine-H), 3.20 – 3.11 (m, 2H, Piperazine-H), 2.50 (d, *J* = 1.7 Hz, 2H, Piperazine-H), 2.38 (s, 2H, Piperazine-H).

^{13}C NMR (100 MHz, DMSO) δ 163.5 (s), 158.5 (d, *J* = 240.0 Hz),158.5 (s),156.4 (s), 156.1 (s), 145.2 (q, *J* = 3.8 Hz), 136.1 (dd, *J* = 7.0, 3.5 Hz), 128.4 (s), 126.9 (d, *J* = 33.2 Hz), 123.0 (q, *J* = 273.3 Hz), 117.9 (d, *J* = 26.5 Hz), 117.2 (dd, *J* = 25.3, 8.9 Hz), 116.0 (d, *J* = 19.8 Hz),54.4 (s), 52.9 (s), 52.2 (s), 46.2 (s), 41.4 (s).

^{19}F NMR (376 MHz, DMSO) δ -60.87 , -118.99 , -123.50 .

HRMS(ESI): Calculated for $C_{18}H_{16}ON_3ClF_5[M+H]^+$: 420.0897, found: 420.0893.

(4-(3-bromobenzyl)piperazin-1-yl)(3-chloro-5-(trifluoromethyl)pyridin-2-yl)methanone (A7)

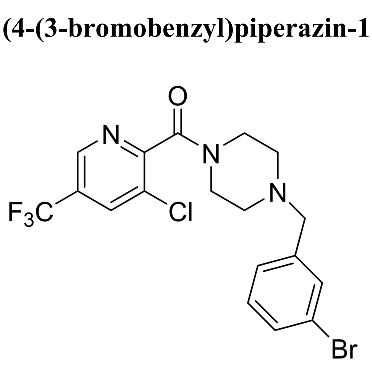

Yield 65.7%; White solid; m. p. 103 - 105°C.

^{1}H NMR (400 MHz, DMSO) δ 8.99 (s, 1H, Ar-H), 8.64 (s, 1H, Ar-H), 7.52 (s, 1H, Ph-H), 7.46 (d, *J* = 7.2 Hz, 1H, Ph-H), 7.35 – 7.22 (m, 2H, Ph-H), 3.68 (s, 2H, -CH_2-), 3.52 (s, 2H, Piperazine-H), 3.19 – 3.09 (m, 2H, Piperazine-H), 2.48 – 2.41

(m, 2H, Piperazine-H), 2.37 – 2.28 (m, 2H, Piperazine-H).

^{13}C NMR (100 MHz, DMSO) δ 163.5 (s), 156.5 (s), 145.2 (d, *J* = 4.0 Hz), 141.2(s), 136.1 (dd, *J* = 6.6, 3.2 Hz), 131.9 (s), 130.9 (s), 130.4 (s), 128.4 (s), 126.9 (d, *J* = 33.3 Hz), 123.0 (q, *J* = 273.3 Hz), 122.1 (s), 61.3 (s), 53.1 (s), 52.4 (s), 46.3 (s), 41.5 (s).

^{19}F NMR (376 MHz, DMSO) δ -60.85 .

HRMS(ESI): Calculated for $C_{18}H_{17}N_3BrClF_3[M+H]^+$: 462.0190, found: 462.0187.

(4-(4-(tert-butyl)benzyl)piperazin-1-yl)(3-chloro-5-(trifluoromethyl)pyridin-2-yl)methanone (A8)

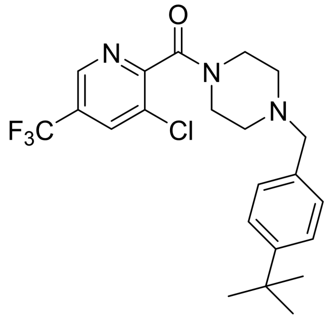

Yield 43.6%; White solid; m. p. 140 - 142°C.

^{1}H NMR (400 MHz, DMSO) δ 8.99 (dd, *J* = 1.8, 0.8 Hz, 1H, Ar-H), 8.64 (d, *J* = 1.3 Hz, 1H, Ar-H), 7.35 (s, 1H, Ph-H), 7.33 (s, 1H, Ph-H), 7.23 (s, 1H, Ph-H), 7.21 (s, 1H, Ph-H), 3.70 – 3.62 (m, 2H, -CH_2-), 3.47 (s, 2H, Piperazine-H), 3.18 – 3.10 (m, 2H, Piperazine-H), 2.45 – 2.39 (m, 2H, Piperazine-H), 2.36 – 2.29 (m, 2H, Piperazine-H), 1.26 (s,9H, -$(CH_3)_3$).

^{13}C NMR (100 MHz, DMSO)δ 163.6 (s), 156.6 (s), 149.9 (s), 145.3 (d, *J* = 3.7 Hz), 136.2 (d, *J* = 3.1 Hz), 135.1 (s), 129.2 (s), 128.4 (s), 126.9 (d, *J* = 33.0 Hz), 125.9 (s), 125.5 (s), 123.1 (q, *J* = 273.5 Hz), 61.9 (s), 53.4 (s), 52.4 (s), 46.4 (s), 41.6(s), 34.7 (s), 31.7 (s), 31.6 (s), 31.5 (s), 31.3 (s).

^{19}F NMR (376 MHz, DMSO) δ -60.84 .

HRMS(ESI): Calculated for $C_{22}H_{26}ON_3ClF_3[M+H]^+$: 440.1711, found: 440.1711.

(3-chloro-5-(trifluoromethyl)pyridin-2-yl)(4-(2,3-dichlorobenzyl)piperazin-1-yl)methanone (A9)

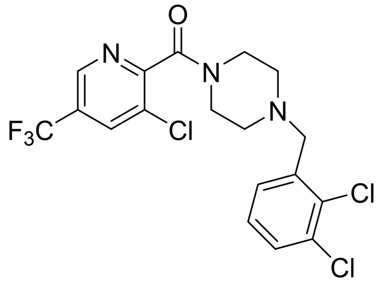

Yield 92.2%; White solid; m. p. 120 - 122°C.

^{1}H NMR (400 MHz, DMSO) δ 9.01 – 8.98 (m, 1H, Ar-H), 8.65 (d, *J* = 1.3 Hz, 1H, Ar-H), 7.56 (dd, *J* = 7.9, 1.5 Hz, 1H, Ph-H), 7.50 (dd, *J* = 7.7, 1.5 Hz, 1H, Ph-H), 7.36 (t, *J* = 7.8 Hz, 1H, Ph-H), 3.74 – 3.67 (m, 2H, -CH_2-), 3.65 (s, 2H,

Piperazine-H), 3.27 – 3.09 (m, 2H, Piperazine-H), 2.60 – 2.51 (m, 2H, Piperazine-H), 2.48 – 2.36 (m, 2H, Piperazine-H).

^{13}C NMR (100 MHz, DMSO) δ 163.6 (s), 156.5 (s), 145.3 (d, *J* = 4.0 Hz), 138.5 (s), 136.12 (d, *J* = 3.6 Hz), 132.2 (s), 131.6 (s), 129.7 (d, *J* = 10.8 Hz), 128.4 (d, *J* = 3.3 Hz), 127.5 (s), 126.9 (d, *J* = 33.2 Hz), 126.5 (s), 123.0 (q, *J* = 273.3 Hz), 59.5 (s), 53.3 (s), 52.6 (s), 46.3 (s), 41.5 (s).

^{19}F NMR (376 MHz, DMSO) δ -60.85 .

HRMS(ESI): Calculated for $C_{18}H_{16}N_3Cl_3F_3[M+H]^+$: 452.0306, found: 452.0304.

(3-chloro-5-(trifluoromethyl)pyridin-2-yl)(4-(4-(trifluoromethoxy)benzyl)piperazin-1-yl)methanone (A10)

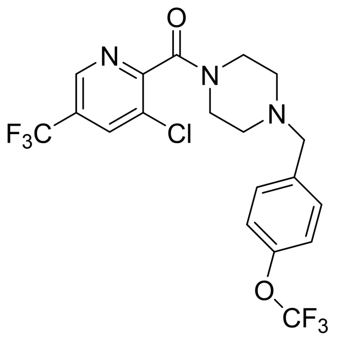

Yield 22.0%; Yellow solid; m. p. 78 - 80℃.

^{1}H NMR (400 MHz, DMSO) δ 8.99 (s, 1H, Ar-H), 8.64 (s, 1H, Ar-H), 7.46 (s, 1H, Ph-H), 7.44 (s, 1H, Ph-H), 7.33 (s, 1H, Ph-H), 7.31 (s, 1H, Ph-H), 3.68 (s, 2H, -CH$_2$-), 3.55 (s, 2H, Piperazine-H), 3.16 (s, 2H, Piperazine-H), 2.46 (s, 2H, Piperazine-H), 2.34 (s, 2H, Piperazine-H).

^{13}C NMR (100 MHz, DMSO) δ 163.5 (s), 156.5 (s), 147.8 (d, *J* = 1.3 Hz), 145.2 (dd, *J* = 8.0, 4.1 Hz), 139.5 – 137.2 (m), 136.1 (t, *J* = 3.2 Hz), 131.4 (s), 131.1 (s), 128.4 (s), 126.8 (s), 125.7 (q, *J* = 273.2 Hz), 122.9 (q, *J* = 273.9 Hz), 121.7 (d, *J* = 3.6 Hz), 121.3 (s), 61.1 (s), 53.2 (s), 52.4 (s), 46.3 (s), 41.5 (s).

^{19}F NMR (376 MHz, DMSO) δ -56.83 , -60.86.

HRMS(ESI): Calculated for $C_{19}H_{17}O_2N_3ClF_6[M+H]^+$: 468.0908, found: 468.0908.

(3-chloro-5-(trifluoromethyl)pyridin-2-yl)(4-(3-fluorobenzyl)piperazin-1-yl)methanone (A11)

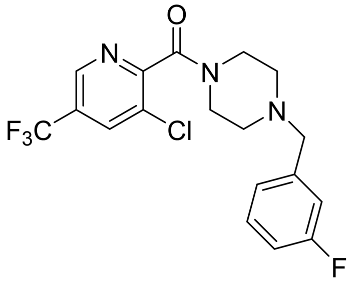

Yield 75.0%; Yellow solid; m. p. 82 - 84℃.

^{1}H NMR (400 MHz, DMSO) δ 8.99 (dd, *J* = 1.8, 0.8 Hz, 1H, Ar-H), 8.66 – 8.62 (m, 1H, Ar-H), 7.39 – 7.33 (m, 1H, Ar-H), 7.21 – 7.07 (m, 3H, Ar-H), 3.75 – 3.64 (m, 2H, -CH$_3$-), 3.54 (s, 2H, Piperazine-H), 3.22 – 3.08 (m, 2H, Piperazine-H), 2.48 – 2.42 (m, 2H, Piperazine-H), 2.37 – 2.30 (m, 2H, Piperazine-H).

^{13}C NMR (100 MHz, DMSO) δ 163.5 (s), 162.7 (d, *J* = 243.3 Hz), 156.5 (s), 145.2 (dd, *J* = 8.1, 3.9 Hz), 141.3 (d, *J* = 7.1 Hz), 136.1 (q, *J* = 3.6 Hz), 130.6 (d, *J* = 8.3 Hz), 128.4 (s), 126.9 (d, *J* = 33.2 Hz), 125.3 (d, *J* = 2.5 Hz), 123.0 (q, *J* = 273.3 Hz), 115.7 (t, *J* = 16.0 Hz), 114.3 (d, *J* = 20.9 Hz), 61.4 (s), 53.2 (s), 52.4 (s), 46.3 (s), 41.5 (s).

^{19}F NMR(376 MHz, DMSO) δ -60.84 , -113.67 .

HRMS(ESI): Calculated for $C_{18}H_{17}ON_3ClF_4[M+H]^+$: 402.0991, found: 402.0989

(3-chloro-5-(trifluoromethyl)pyridin-2-yl)(4-(2-fluoro-5-(trifluoromethyl)benzyl)piperazin-1-yl)methanone (A12)

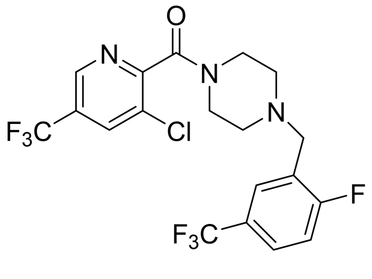

Yield 40.0%; White solid; m. p. 85 - 87℃.

^{1}H NMR (400 MHz, DMSO) δ 8.99 (dd, *J* = 1.8, 0.8 Hz, 1H, Ar-H), 8.64 (d, *J* = 1.3 Hz, 1H, Ar-H), 7.81 (dd, *J* = 6.4, 2.1 Hz, 1H, Ph-H), 7.77 – 7.69 (m, 1H, Ph-H), 7.44 (t, *J* = 9.1 Hz, 1H, Ph-H), 3.71 – 3.63 (m, 4H, -CH2- and Piperazine-H), 3.27 – 3.06 (m, 2H, Piperazine-H), 2.55 – 2.43 (m, 2H, Piperazine-H), 2.43 – 2.27 (m,2H, Piperazine-H).

^{13}C NMR (100 MHz, DMSO) δ 163.5 (s), 163.3 (d, *J* = 250.1 Hz), 156.4 (s), 145.2 (d, *J* = 4.2 Hz), 136.1 (d, *J* = 3.4 Hz), 129.2 (td, *J* = 7.5, 3.6 Hz), 128.4 (s), 127.5 – 126.9 (m), 126.8 (d, *J* = 5.3 Hz), 126.3 (s), 125.9 – 125.4 (m), 124.4 (q, *J* = 273.9 Hz), 123.0 (q, *J* = 273.3 Hz), 117.0 (dd, *J* = 23.7, 8.5 Hz), 54.3 (d, *J* = 5.9 Hz), 52.8 (d, *J* = 13.6 Hz), 52.3 (s), 46.2 (s), 41.4 (s).

^{19}F NMR (376 MHz, DMSO) δ -60.34 , -60.88, -111.57 .

HRMS(ESI): Calculated for $C_{19}H_{16}ON_3ClF_7[M+H]^+$: 470.0865, found: 470.0865.

(3-chloro-5-(trifluoromethyl)pyridin-2-yl)(4-(2-chlorobenzyl)piperazin-1-yl)methanone (A13)

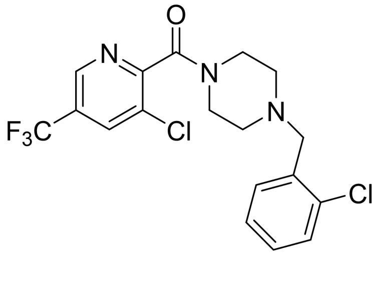

Yield 47.0%; White solid; m. p. 120 - 122℃.

^{1}H NMR (400 MHz, DMSO) δ 9.01 – 8.97 (m, 1H, Ar-H), 8.65 (d, *J* = 1.3 Hz, 1H, Ar-H), 7.51 (dd, *J* = 7.4, 1.9 Hz, 1H, Ph-H), 7.44 (dd, *J* = 7.6, 1.6 Hz, 1H, Ph-H), 7.36 – 7.28 (m, 2H, Ph-H), 3.72 – 3.65 (m, 2H, $-CH_2-$), 3.62 (s, 2H,

Piperazine-H), 3.21 – 3.12 (m, 2H, Piperazine-H), 2.54 – 2.49 (m, 2H, Piperazine-H), 2.43 – 2.35 (m, 2H, Piperazine-H).

^{13}C NMR (100 MHz, DMSO) δ 163.6 (s), 156.4 (s), 145.2 (q, *J* = 3.8 Hz), 136.5 – 135.9 (m), 135.6 (s), 133.8 (s), 131.5 (s), 129.8 (s), 129.3 (s), 128.4 (s), 127.5 (s), 126.9 (d, *J* = 33.2 Hz), 123.0 (q, *J* = 273.3 Hz), 58.8 (s), 53.3 (s), 52.6 (s), 46.3 (s), 41.5 (s).

^{19}F NMR (376 MHz, DMSO) δ -60.86.

HRMS(ESI): Calculated for $C_{18}H_{17}ON_3Cl_2F_3[M+H]^+$: 418.0695, found: 418.0694.

(3-chloro-5-(trifluoromethyl)pyridin-2-yl)(4-(3,5-difluorobenzyl)piperazin-1-yl)methanone (A14)

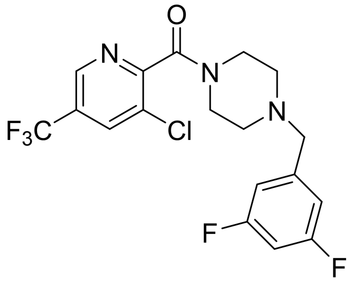

Yield 47.0%; White solid; m. p. 102 - 104°C.

^{1}H NMR (400 MHz, DMSO) δ 9.00 – 8.97 (m, 1H, Ar-H), 8.64 (d, *J* = 1.3 Hz, 1H, Ar-H), 7.12 (t, *J* = 2.3 Hz, 1H, Ph-H), 7.06 (d, *J* = 2.0 Hz, 1H, Ph-H), 7.04 (d, *J* = 2.0 Hz, 1H, Ph-H), 3.72 – 3.65 (m, 2H, -CH_2-), 3.56 (s, 2H, Piperazine-H), 3.23 – 3.08 (m, 2H, Piperazine-H), 2.49 – 2.44 (m, 2H, Piperazine-H), 2.37 – 2.31 (m, 2H, Piperazine-H).

^{13}C NMR (100 MHz, DMSO) δ 163.6 (s), 162.9 (d, *J* = 246.0 Hz), 162.7 (d, *J* = 245.9 Hz),145.24 (dd, *J* = 8.0, 4.0 Hz), 143.3 (t, *J* = 8.9 Hz), 136.1 (dd, *J* = 6.9, 3.5 Hz), 128.4 (s), 126.9 (d, *J* = 33.2 Hz), 123.0 (q, *J* = 273.3 Hz), 112.4 – 111.8 (m), 111.7 (d, *J* = 6.5 Hz), 103.2 (s), 102.8 (dd, *J* = 25.9, 6.4 Hz), 60.8 (s), 53.0 (s), 52.3 (s), 46.3 (s), 41.5 (s).

^{19}F NMR (376 MHz, DMSO) δ -60.86 , -110.29.

HRMS(ESI): Calculated for $C_{18}H_{16}ON_3ClF_5[M+H]^+$: 420.0897, found: 420.0894.

(3-chloro-5-(trifluoromethyl)pyridin-2-yl)(4-(4-chlorobenzyl)piperazin-1-yl)methanone (A15)

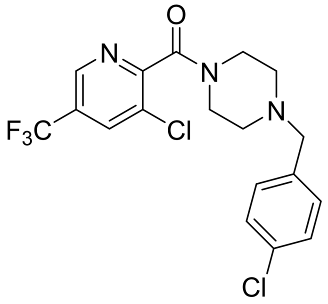

Yield 41.0%; White solid; m. p. 106 - 108°C.

^{1}H NMR (400 MHz, DMSO) δ 8.99 (dd, *J* = 1.8, 0.8 Hz, 1H, Ar-H), 8.66 – 8.62 (m, 1H, Ar-H), 7.38 (d, *J* = 2.1 Hz, 2H, Ph-H), 7.35 (d, *J* = 3.1 Hz, 2H, Ph-H), 3.70 – 3.64 (m, 2H, -CH_2-), 3.51 (s, 2H, Piperazine-H), 3.21 – 3.08 (m, 2H, Piperazine-H), 2.47 – 2.40

(m, 2H, Piperazine-H), 2.37 – 2.26 (m, 2H, Piperazine-H).

^{13}C NMR (100 MHz, DMSO) δ 163.5 (s), 156.5 (s), 145.2 (q, *J* = 4.0 Hz), 137.2 (s), 136.1 (q, *J* = 3.2 Hz), 132.0 (s), 131.1 (s), 128.6 (s), 128.4 (s), 127.4 (s), 126.9 (d, *J* = 33.2 Hz), 126.4 (s), 123.0 (q, *J* = 273.3 Hz), 61.2 (s), 53.1 (s), 52.3 (s), 46.2 (s), 41.5 (s).

^{19}F NMR (376 MHz, DMSO) δ -60.85 (s).

HRMS(ESI): Calculated for $C_{18}H_{17}N_3Cl_2F_3[M+H]^+$: 418.0695, found: 418.0692.

(4-(2-chloro-4-fluorobenzyl)piperazin-1-yl)(3-chloro-5-(trifluoromethyl)pyridin-2-yl)methanone (A16)

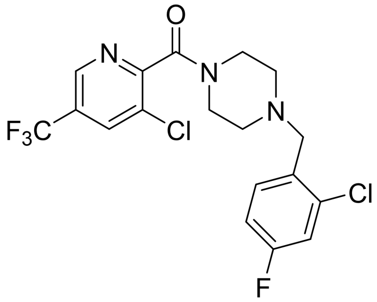

Yield 30.0%; White solid; m. p. 109 - 111°C.

^{1}H NMR (400 MHz, DMSO) δ 8.99 (dd, *J* = 1.7, 0.7 Hz, 1H, Ar-H), 8.65 (d, *J* = 1.3 Hz, 1H, Ar-H), 7.54 (dd, *J* = 8.6, 6.5 Hz, 1H, Ph-H), 7.42 (dd, *J* = 8.9, 2.6 Hz, 1H, Ph-H), 7.22 (td, *J* = 8.5, 2.7 Hz, 1H, Ph-H), 3.71 – 3.65 (m, 2H,-CH2-), 3.58 (s, 2H, Piperazine-H), 3.20 – 3.10 (m, 2H, Piperazine-H), 2.51 – 2.49 (m, 2H, Piperazine-H), 2.42 – 2.36 (m, 2H, Piperazine-H).

^{13}C NMR (100 MHz, DMSO) δ 163.6 (s), 161.5 (d, *J* = 246.6 Hz), 156.5 (s), 145.3 (d, *J* = 3.7 Hz), 136.1 (d, *J* = 3.1 Hz), 134.6 (d, *J* = 10.5 Hz), 132.9 (d, *J* = 8.6 Hz), 132.0 (d, *J* = 3.2 Hz), 128.5 (s), 127.0 (d, *J* = 33.0 Hz), 123.0 (d, *J* = 273.4 Hz), 117.0 (d, *J* = 24.9 Hz), 114.7 (d, *J* = 20.7 Hz), 58.1 (s), 53.2 (s), 52.5 (s), 46.3 (s), 41.6 (s).

^{19}F NMR (471 MHz, DMSO) δ -60.74 , -113.26 .

HRMS(ESI): Calculated for $C_{18}H_{16}ON_3Cl_2F_4[M+H]^+$: 436.0601, found: 436.0600.

(4-benzylpiperazin-1-yl)(3-chloro-5-(trifluoromethyl)pyridin-2-yl)methanone (A17)

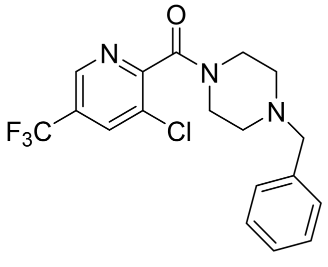

Yield 22.0%; White solid; m. p. 115 - 117°C.

^{1}H NMR (400 MHz, DMSO) δ 9.01 – 8.97 (m, 1H, Ar-H), 8.64 (d, *J* = 1.3 Hz, 1H, Ar-H), 7.34 – 7.24 (m, 5H, Ph-H), 3.72 – 3.62 (m, 2H, -CH2-), 3.51 (s, 2H, Piperazine-H), 3.20 – 3.07 (m, 2H, Piperazine-H), 2.47 – 2.39 (m, 2H, Piperazine-H), 2.35 – 2.30 (m, 2H, Piperazine-H).

^{13}C NMR (100 MHz, DMSO) δ 163.5 (s), 156.4 (s), 145.2 (dd, *J* = 9.1, 4.7 Hz), 138.0 (s), 136.1 (dd, *J* = 8.1, 4.1 Hz), 129.4 (s), 128.7 (s), 128.4 (s), 127.6 (s), 127.1 (s), 123.4 (s), 123.0 (q, *J* = 273.3 Hz), 121.8 (s), 62.1 (s), 53.1 (s), 52.4 (s), 46.3 (s), 41.5 (s).

^{19}F NMR (376 MHz, DMSO) δ -60.84.

HRMS(ESI): Calculated for $C_{18}H_{18}ON_3Cl_2F_3[M+H]^+$: 384.1085, found: 384.1085.

(3-chloro-5-(trifluoromethyl)pyridin-2-yl)(4-(4-(trifluoromethyl)benzyl)piperazin-1-yl)methanone (A18)

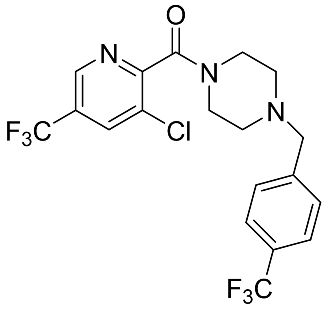

Yield 32.0%; White solid; m. p. 102 - 104°C.

^{1}H NMR (400 MHz, DMSO) δ 8.99 (d, *J* = 1.0 Hz, 1H, Ar-H), 8.64 (d, *J* = 1.3 Hz, 1H, Ar-H), 7.68 (t, *J* = 8.0 Hz, 2H, Ph-H), 7.55 – 7.48 (m, 2H, Ph-H), 3.73 – 3.64 (m, 2H, -CH$_2$-), 3.62 (s, 2H, Piperazine-H), 3.20 – 3.09 (m, 2H, Piperazine-H), 2.49 – 2.42 (m, 2H, Piperazine-H), 2.40 – 2.30 (m, 2H, Piperazine-H).

^{13}C NMR (100 MHz, DMSO) δ 163.6 (s), 156.5 (s), 145.2 (q, *J* = 3.9 Hz), 143.3 (s), 136.1 (q, *J* = 3.3 Hz), 129.9 (s), 128.7 (d, *J* = 18.5 Hz), 128.4 (d, *J* = 7.2 Hz), 127.9 (d, *J* = 31.5 Hz), 126.9 (d, *J* = 33.1 Hz), 126.4 (s), 125.5 (q, *J* = 3.6 Hz), 123.0 (q, *J* = 273.3 Hz),124.8 (q, *J* = 271.9 Hz), 61.4 (s), 53.2 (s), 52.4 (s), 46.3 (s), 41.5 (s).

^{19}F NMR (376 MHz, DMSO) δ -60.87.

HRMS(ESI): Calculated for $C_{19}H_{17}ON_3ClF_6[M+H]^+$: 452.0959, found: 452.0957.

(3-chloro-5-(trifluoromethyl)pyridin-2-yl)(4-(2,4-difluorobenzyl)piperazin-1-yl)methanone (A19)

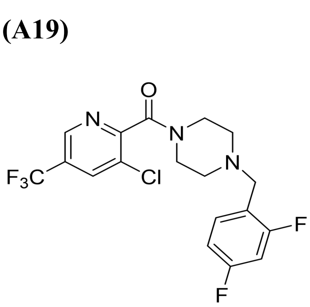

Yield 35.0%; White solid; m. p. 92 - 94°C.

^{1}H NMR (400 MHz, DMSO) δ 8.99 (d, *J* = 0.9 Hz, 1H, Ar-H), 8.64 (d, *J* = 1.3 Hz, 1H, Ar-H), 7.46 (d, *J* = 6.9 Hz, 1H, Ph-H), 7.22 (d, *J* = 2.0 Hz, 1H, Ph-H), 7.08 (d, *J* = 2.0 Hz, 1H, Ph-H), 3.69 – 3.63 (m, 2H, -CH$_2$-), 3.56 (s, 2H, Piperazine-H), 3.21 – 3.05 (m, 2H, Piperazine-H), 2.49 – 2.44 (m, 2H, Piperazine-H), 2.38 – 2.28 (m, 2H, Piperazine-H).

^{13}C NMR (100 MHz, DMSO) δ 163.5 (s), 162.0 (dd, *J* = 245.6, 12.3 Hz), δ 161.2 (dd, *J* = 247.5, 12.4 Hz),156.4 (d, *J* = 1.3 Hz), 145.2 (d, *J* = 4.0 Hz), 136.1 (d, *J* = 3.6 Hz), 133.9 – 132.0 (m), 128.4 (s), 126.9 (d, *J* = 33.3 Hz), 123.0 (q, *J* = 273.3 Hz), 120.8 (dd, *J* = 15.0, 3.7 Hz), 111.7 (dd, *J* = 20.9, 3.5 Hz), 104.1 (t, *J* = 26.1 Hz), 54.3 (s), 52.8 (s), 52.2 (s), 46.2 (s), 41.5 (s).

^{19}F NMR (376 MHz, DMSO) δ -60.85, -111.53 , -113.43 .

HRMS(ESI): Calculated for $C_{18}H_{16}ON_3ClF_5[M+H]^+$: 420.0897, found: 420.0896.

(3-chloro-5-(trifluoromethyl)pyridin-2-yl)(4-(2-(trifluoromethyl)benzyl)piperazin-1-yl)methanone (A20)

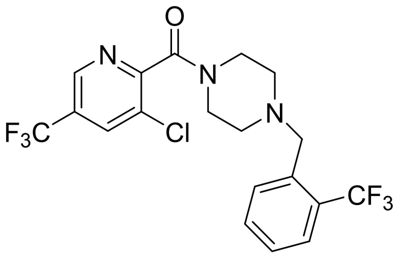

Yield 30.0%; White solid; m. p. 100 - 102℃.

^{1}H NMR (400 MHz, DMSO) δ 9.00 (d, *J* = 1.0 Hz, 1H, Ar-H), 8.65 (d, *J* = 1.3 Hz, 1H, Ar-H), 7.80 (d, *J* = 7.7 Hz, 1H, Ph-H), 7.74 – 7.65 (m, 2H, Ph-H), 7.48 (t, *J* = 7.7 Hz, 1H, Ph-H), 3.74 – 3.68 (m, 2H, -CH_2-), 3.67 (s, 2H, Piperazine-H), 3.22 – 3.14 (m, 2H, Piperazine-H), 2.50 – 2.47 (m, 2H, Piperazine-H), 2.39 – 2.34 (m, 2H, Piperazine-H).

^{13}C NMR (100 MHz, DMSO) δ 163.5 (s), 156.4 (s), 145.2 (dd, *J* = 8.2, 3.9 Hz), 137.3 (d, *J* = 1.4 Hz), 136.1 (d, *J* = 3.5 Hz), 133.0 (s), 131.1 (s), 128.2 (d, *J* = 41.9 Hz), 127.7 (d, *J* = 29.7 Hz), 126.9 (d, *J* = 33.2 Hz), 126.2 (d, *J* = 5.8 Hz), 123.0 (q, *J* = 273.3 Hz),122.1 (q, *J* = 274.1 Hz), 57.8 (s), 53.3 (s), 52.6 (s), 46.3 (s), 41.5 (s).

^{19}F NMR (376 MHz, DMSO) δ -57.85 (s), -60.85 (s).

HRMS(ESI): Calculated for $C_{19}H_{17}ON_3ClF_6[M+H]^+$: 452.0959, found: 452.0958.

(4-(3-chloro-4-fluorobenzyl)piperazin-1-yl)(3-chloro-5-(trifluoromethyl)pyridin-2-yl)methanone (A21)

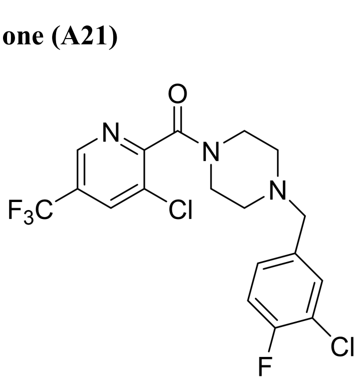

Yield 38.0%; White solid; m. p. 102 - 104℃.

^{1}H NMR (400 MHz, DMSO) δ 8.99 (dd, *J* = 1.7, 0.7 Hz, 1H, Ar-H), 8.64 (d, *J* = 1.3 Hz, 1H, Ar-H), 7.54 (dd, *J* = 8.6, 6.5 Hz, 1H, Ph-H), 7.42 (dd, *J* = 8.9, 2.6 Hz, 1H, Ph-H), 7.22 (td, *J* = 8.5, 2.7 Hz, 1H, Ph-H), 3.68 (s, 2H, -CH_2-), 3.59 (s, 2H,

Piperazine-H), 3.19 – 3.07 (m, 2H, Piperazine-H), 2.52 – 2.50 (m,2H, Piperazine-H), 2.44 – 2.32 (m, 2H, Piperazine-H).

^{13}C NMR (100 MHz, DMSO) δ 163.5 (s), 161.4 (d, *J* = 246.7 Hz), 156.4 (s), 145.2 (dd, *J* = 7.8, 3.9 Hz), 136.8 – 135.7 (m), 134.5 (d, *J* = 10.6 Hz), 132.8 (d, *J* = 8.8 Hz), 132.0 (d, *J* = 3.3 Hz), 128.5 (s), 126.9 (d, *J* = 33.2 Hz), 123.0 (q, *J* = 273.3 Hz), 116.9 (d, *J* = 24.9 Hz), 114.7 (d, *J* = 20.8 Hz), 58.1 (s), 53.1 (s), 52.4 (s), 46.2 (s), 41.5 (s).

^{19}F NMR (376 MHz, DMSO) δ -60.89 (s), -113.37 (dd, *J* = 16.0, 9.1 Hz).

HRMS(ESI): Calculated for $C_{18}H_{16}ON_3Cl_2F_4[M+H]^+$: 436.0601, found: 436.0601.

(3-chloro-5-(trifluoromethyl)pyridin-2-yl)(4-(3,4-difluorobenzyl)piperazin-1-yl)methanone (A22)

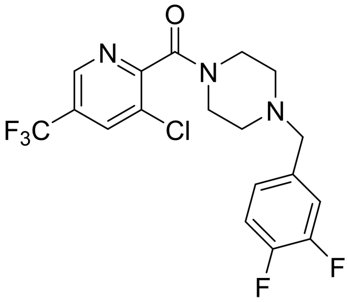

Yield 32.0%; White solid; m. p. 88 - 96℃.

^{1}H NMR (500 MHz, DMSO) δ 8.95 (dd, *J* = 1.8, 0.8 Hz,1H, Ar-H), 8.60 (dd, *J* = 1.9, 0.6 Hz,1H, Ar-H), 7.35 – 7.30 (m,2H, Ph-H), 7.12 (ddd, *J* = 6.2, 3.7, 1.5 Hz, 1H, Ph-H), 3.70 – 3.58 (m, 2H, -CH_2-), 3.47 (s, 2H, Piperazine-H), 3.15 – 3.05 (m, 2H, Piperazine-H), 2.40 (t, *J* = 5.0 Hz, 2H, Piperazine-H), 2.33 – 2.26 (m, 2H, Piperazine-H).

^{13}C NMR (126 MHz, DMSO) δ 163.6 (s), 156.4 (s), 149.7 (dd, *J* = 245.3, 12.7 Hz), 148.9 (dd, *J* = 244.4, 12.6 Hz),145.6 – 144.9 (m), 136.6 – 135.3 (m), 128.4 (s), 126.9 (d, *J* = 33.2 Hz), 125.9 (dd, *J* = 6.5, 3.2 Hz), 123.0 (q, *J* = 273.4 Hz), 117.8 (dd, *J* = 37.8, 16.8 Hz), 60.7 (s), 53.0 (s), 52.2 (s), 46.2 (s), 41.5 (s).

^{19}F NMR (376 MHz, DMSO) δ -60.87 (s), -138.80 – -140.69 (m), -139.99 – -142.57 (m).

HRMS(ESI): Calculated for $C_{18}H_{16}ON_3ClF_5[M+H]^+$: 420.0897, found: 420.0893.

(3-chloro-5-(trifluoromethyl)pyridin-2-yl)(4-(pyridin-2-ylmethyl)piperazin-1-yl)methanone (A23)

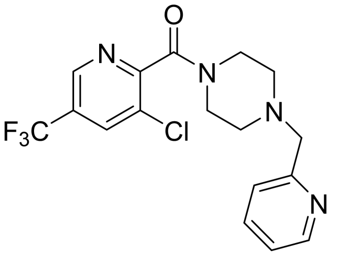

Yield 47.0%; White solid; m. p. 104 - 106℃.

^{1}H NMR (400 MHz, DMSO) δ 9.02 – 8.97 (m, 1H, Ar-H), 8.64 (d, *J* = 1.3 Hz, 1H, Ar-H), 8.52 – 8.45 (m, 1H, Ph-H), 7.78 (td, *J* =

7.7, 1.8 Hz, 1H, Ph-H), 7.46 (d, J = 7.8 Hz, 1H, Ph-H), 7.27 (ddd, J = 7.5, 4.9, 1.0 Hz, 1H, Ph-H), 3.74 – 3.66 (m, 2H, $-CH_2-$), 3.65 (s, 2H, Piperazine-H), 3.20 – 3.12 (m, 2H, Piperazine-H), 2.51 – 2.49 (m, 2H, Piperazine-H), 2.44 – 2.38 (m, 2H, Piperazine-H).

^{13}C NMR (100 MHz, DMSO) δ 163.6 (s), 158.3 (s), 156.5 (s), 149.4 (s), 145.3 (s), 137.1 (s), 136.2 (s), 128.2 (s), 127.1 (s), 123.4 (s), 122.8 (s), 120.0 (q, J = 275.5 Hz), 63.9 (s), 53.4 (s), 52.7 (s), 46.4 (s), 41.6 (s).

^{19}F NMR (376 MHz, DMSO) δ -60.73 (s).

HRMS(ESI): Calculated for $C_{17}H_{17}ON_4ClF_3[M+H]^+$: 385.1038, found: 385.1036.

(3-chloro-5-(trifluoromethyl)pyridin-2-yl)(4-((6-chloropyridin-3-yl)methyl)piperazin-1-yl)methanone (A24)

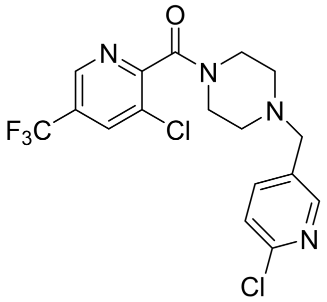

Yield 46.2%; White solid; m. p. 102 - 104℃.

^{1}H NMR (500 MHz,DMSO) δ 8.94 (s,1H, Ar-H), 8.60 (s,1H, Ar-H), 8.30 (s,1H, , Ar-H), 7.76 (d, J = 7.8 Hz,1H, Ar-H), 7.45 (d, J = 8.1 Hz,1H, Ar-H), 3.63 (m,2H, $-CH_2-$), 3.51 (s,2H, Piperazine-H), 3.11 (m,2H, Piperazine-H), 2.42 (m,2H, Piperazine-H), 2.30 (m,2H, Piperazine-H).

^{13}C NMR (126 MHz, DMSO)δ 163.6 (s), 156.5 (s), 150.7 (s), 149.6 (s), 145.3 (d, J = 3.4 Hz), 140.9 (s), 136.2 (d, J = 2.6 Hz), 133.4 (s), 128.5 (s), 127.0 (d, J = 33.5 Hz), 124.5 (s), 123.1 (q, J = 273.5 Hz), 58.2 (s), 53.1 (s), 52.3 (s), 46.3 (s), 41.5 (s).

^{19}F NMR (376 MHz, DMSO)δ -60.76 (s).

HRMS(ESI): Calculated for $C_{17}H_{16}ON_4Cl_2F_3[M+H]^+$: 419.0648, found: 419.0646.

4-(3-chloro-5-(trifluoromethyl)picolinoyl)-N,N-dimethylpiperazine-1-sulfonamide (A25)

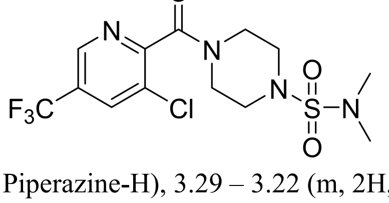

Yield 46.0%; White solid; m. p. 131 - 133℃.

^{1}H NMR (400 MHz, DMSO) δ 9.02 (d, J = 1.0 Hz, 1H, Ar-H), 8.68 (d, J = 1.3 Hz, 1H, Ar-H), 3.84 – 3.66 (m, 2H, Piperazine-H), 3.29 – 3.22 (m, 2H, Piperazine-H), 3.22 (d, J = 6.7 Hz, 2H, Piperazine-H), 3.14 – 3.01 (m, 2H, Piperazine-H), 2.78 (d, J = 2.2 Hz, 6H, $-N(CH_3)_2$).

^{13}C NMR (100 MHz, DMSO) δ 163.9 (s), 155.9 (s), 145.3 (s), 136.2 (s), 129.4 – 128.8 (m), 127.1 (d, J = 33.5 Hz), 125.2 (q, J = 272.9 Hz), 46.8 (s), 46.3 (s), 46.0 (s), 41.2 (s), 38.3 (s).

^{19}F NMR (376 MHz, DMSO) δ -60.85 .

HRMS(ESI): Calculated for $C_{13}H_{17}O_3N_4ClF_3S[M+H]^+$: 401.0657, found: 401.0656.

(3-chloro-5-(trifluoromethyl)pyridin-2-yl)(4-((4-methylbenzyl)sulfonyl)piperazin-1-yl)methanone (A26)

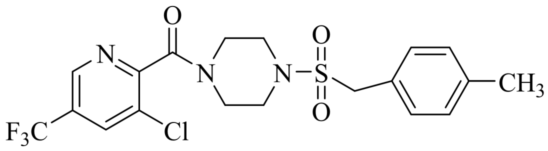

Yield 47.0%; White solid; m. p. 192 - 194°C.

^{1}H NMR (400 MHz, DMSO) δ 8.99 (dd, *J* = 1.7, 0.7 Hz, 1H, Ar-H), 8.65 (d, *J* = 1.3 Hz, 1H, Ar-H), 7.30 (d, *J* = 8.0 Hz, 2H, Ph-H), 7.20 (d, *J* = 7.9 Hz, 2H, Ph-H), 4.44 (s, 2H, -CH2-), 3.79 – 3.65 (m, 2H, Piperazine-H), 3.29 – 3.21 (m, 2H, Piperazine-H), 3.20 – 3.14 (m, 2H, Piperazine-H), 3.04 – 2.97 (m, 2H, Piperazine-H), 2.31 (s, 3H, -CH3).

^{13}C NMR (100 MHz, DMSO) δ 163.8 (s), 155.9 (s), 145.3 (dd, *J* = 8.1, 4.1 Hz), 138.2 (s), 136.2 (q, *J* = 3.7 Hz), 131.2 (s), 129.5 (s), 128.5 (s), 127.1 (d, *J* = 33.2 Hz), 126.5 (s), 122.9 (q, *J* = 273.3 Hz), 55.0 (s), 46.3 (d, *J* = 3.6 Hz), 45.6 (s), 41.5 (s), 21.2 (s).

^{19}F NMR (376 MHz, DMSO) δ -60.87 (s).

HRMS(ESI): Calculated for $C_{19}H_{20}O_3N_3ClF_3S[M+H]^+$: 462.0861, found: 462.0852

(3-chloro-5-(trifluoromethyl)pyridin-2-yl)(4-(piperidin-1-ylsulfonyl)piperazin-1-yl)methanone (A27)

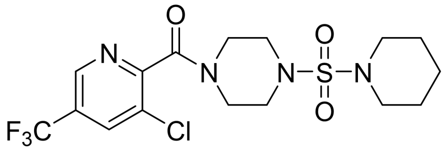

Yield 44.0%; White solid; m. p. 128 - 130°C.

^{1}H NMR (400 MHz, DMSO) δ 9.01 (dd, *J* = 1.7, 0.7 Hz, 1H, Ar-H), 8.66 (d, *J* = 1.3 Hz, 1H, Ar-H), 3.83 – 3.65 (m, 2H, Piperazine-H), 3.31 – 3.21 (m, 4H, Piperazine-H), 3.21 – 3.13 (m, 4H, Piperidine-H), 3.12 – 3.02 (m, 2H, Piperazine-H), 1.50 (s, 6H, Piperidine-H).

^{13}C NMR (100 MHz, DMSO) δ 163.8 (s), 155.9 (s), 145.3 (dd, *J* = 8.3, 4.2 Hz), 138.7 – 133.6 (m), 128.5 (s), 127.1 (d, *J* = 33.4 Hz), 123.0 (q, *J* = 273.2 Hz), 47.2 (s), 46.7 (s), 46.2 (s), 45.9 (s), 41.2 (s), 25.6 (s), 23.6 (s).

^{19}F NMR (376 MHz, DMSO) δ -60.87 (s).

HRMS(ESI): Calculated for $C_{16}H_{21}O_3N_4ClF_3S[M+H]^+$: 441.0969, found: 441.0962

4. NMR Spectra and HRMS spectra of Products

A1 ^{1}H NMR

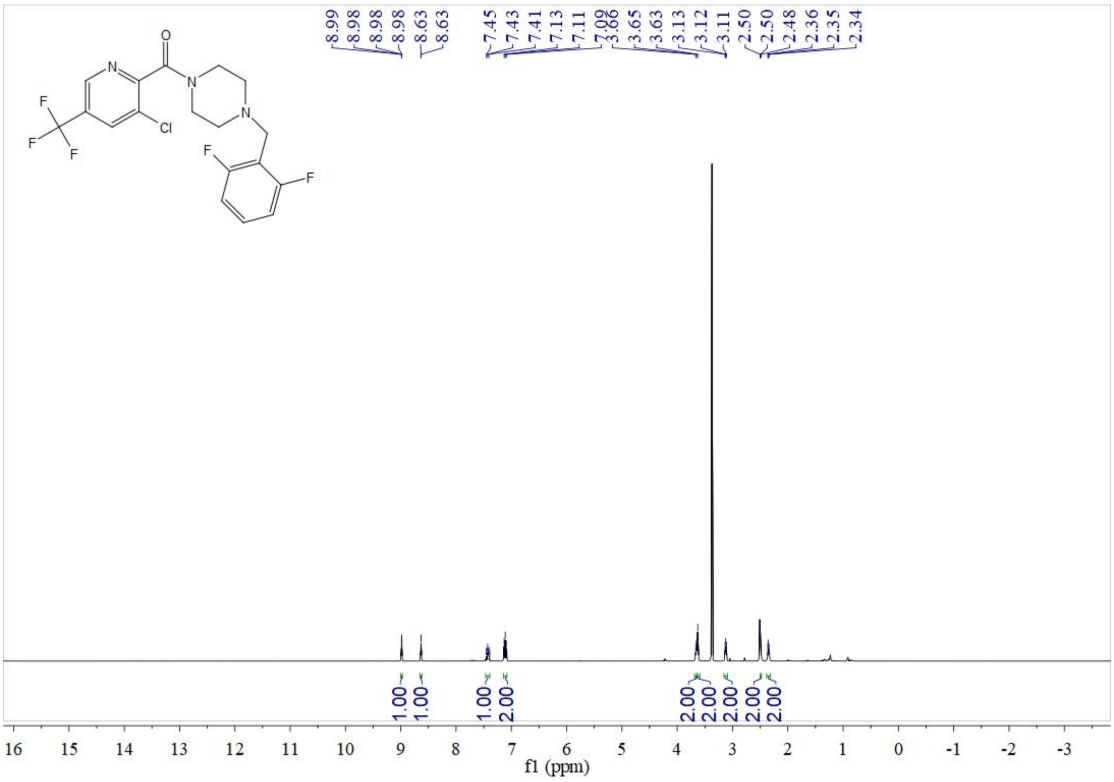

A1 ^{13}C NMR

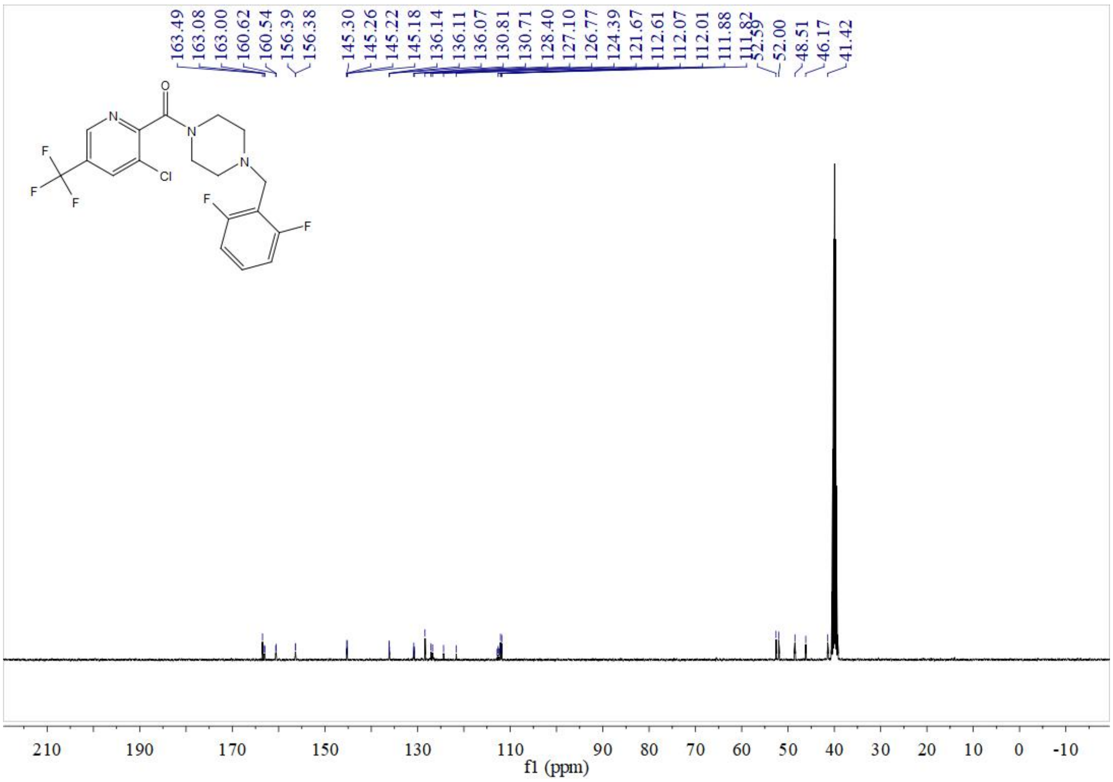

A1 ^{19}F NMR

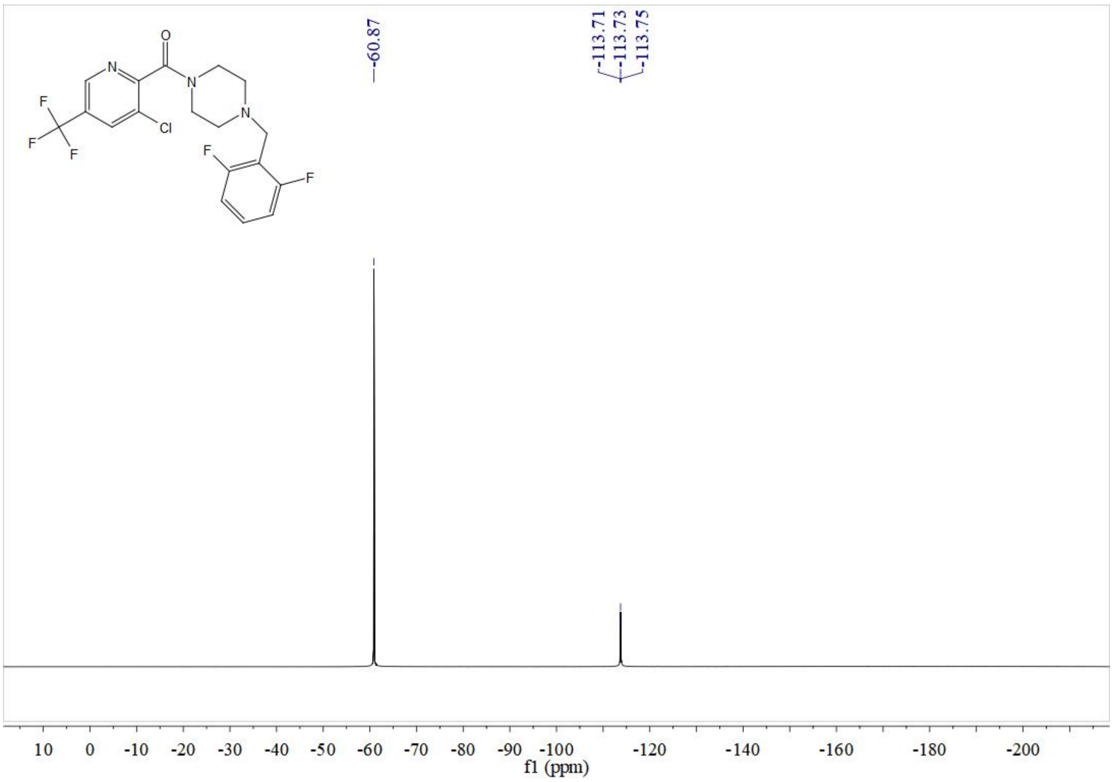

A1 HRMS

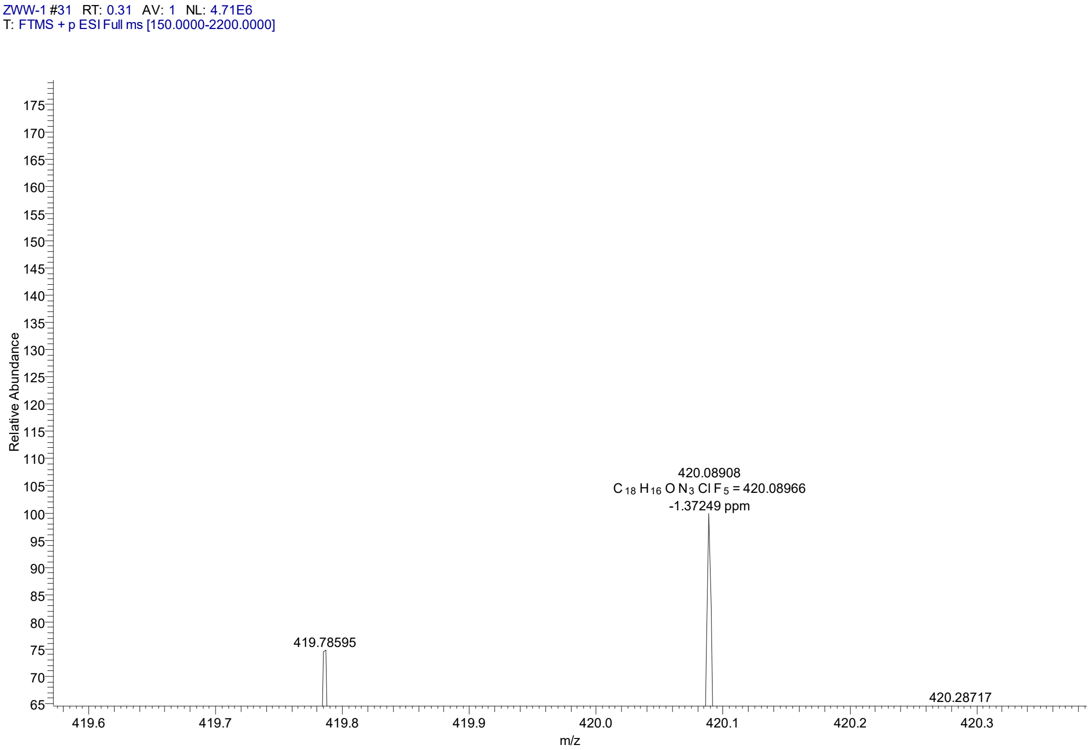

A2 ^{1}H NMR

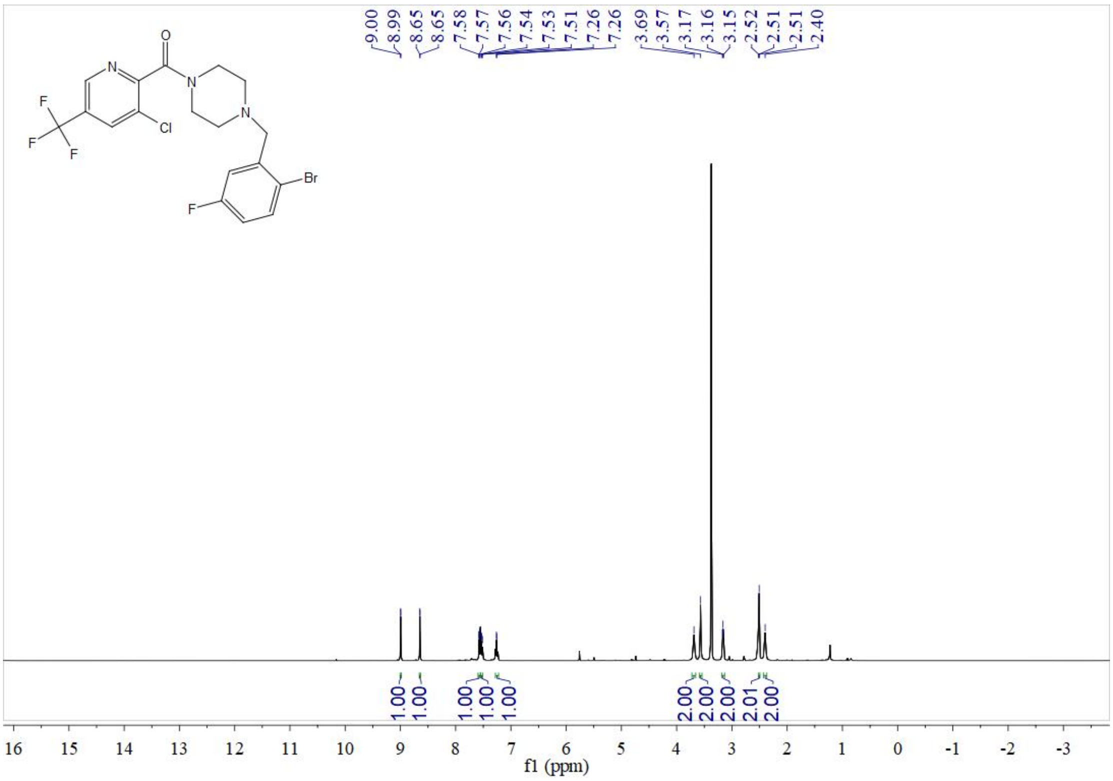

A2 ^{13}C NMR

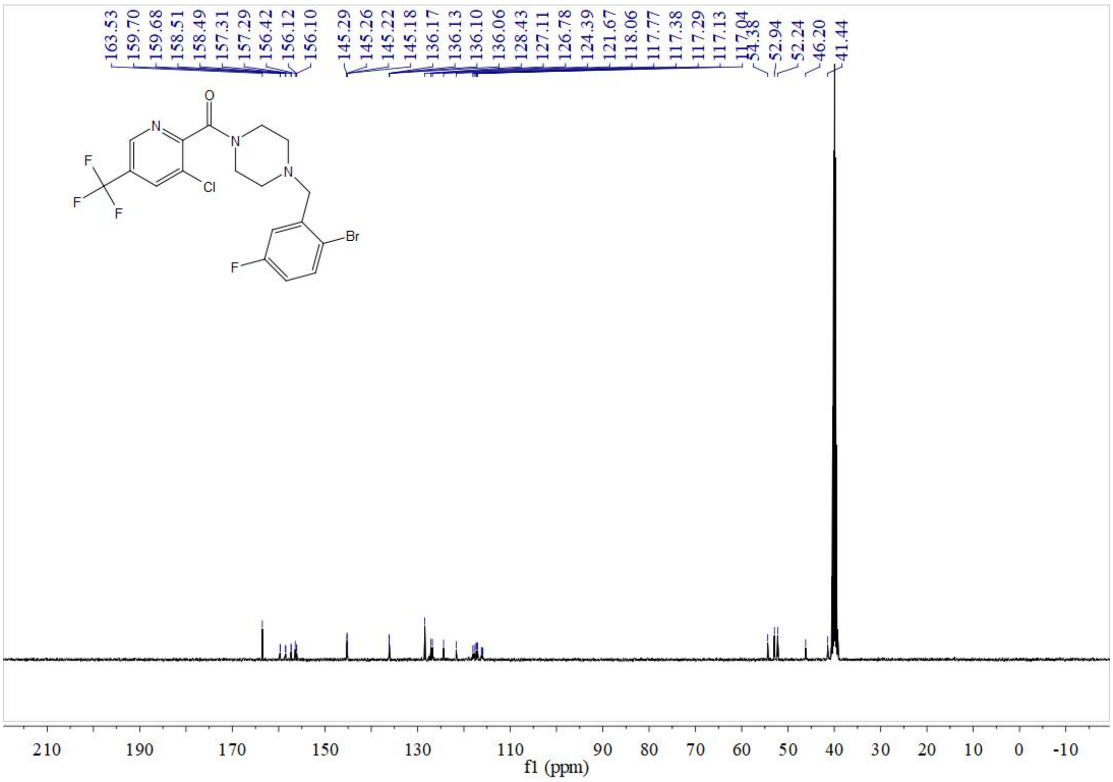

A2 ^{19}F NMR

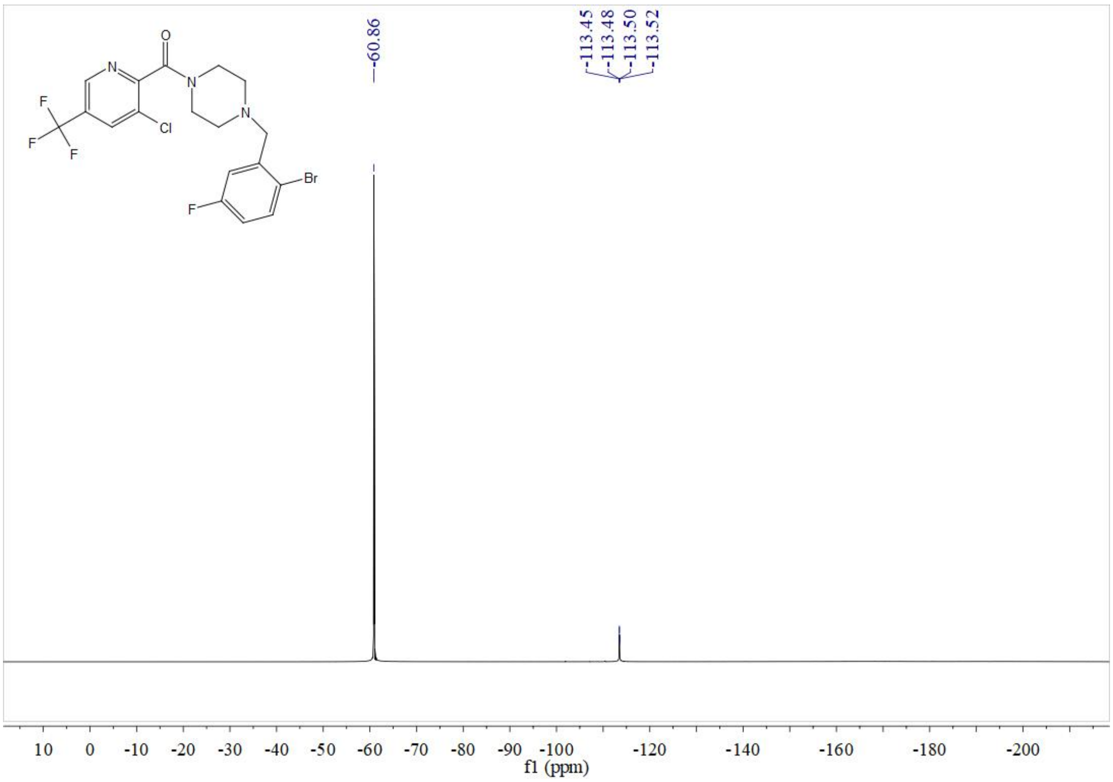

A2 HRMS

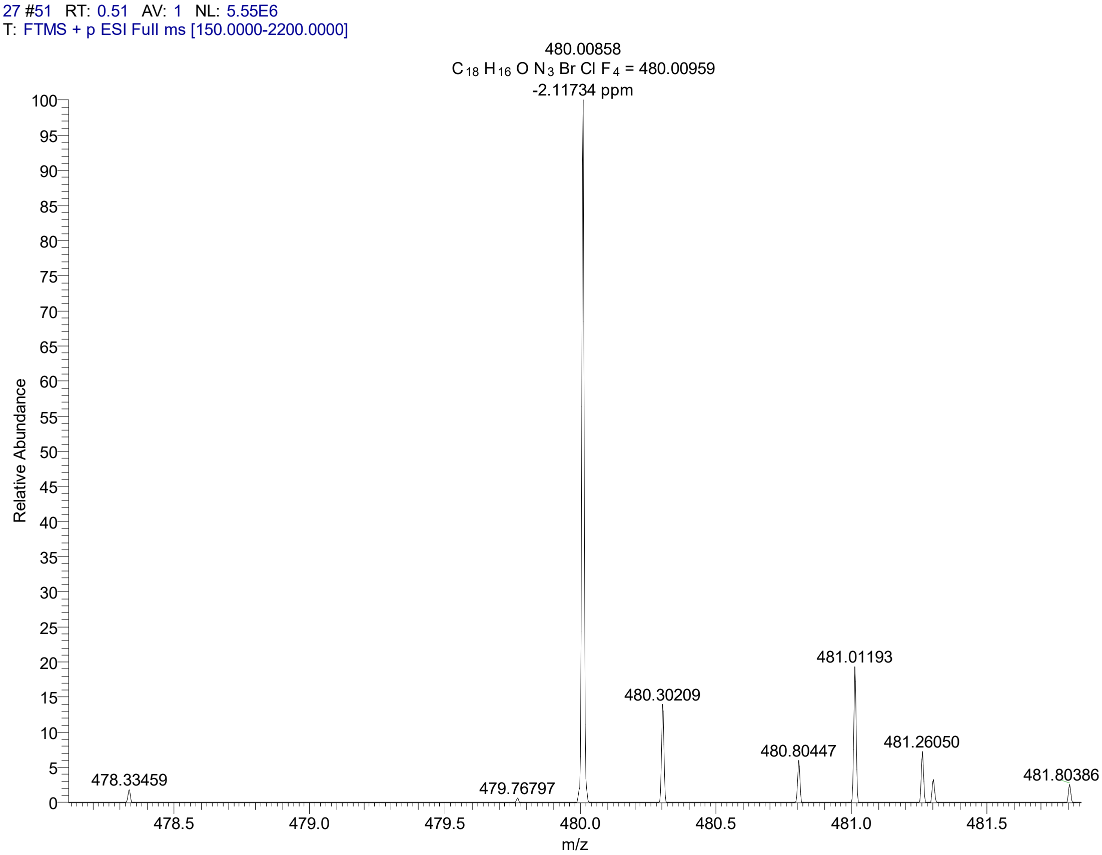

A3 ^{1}H NMR

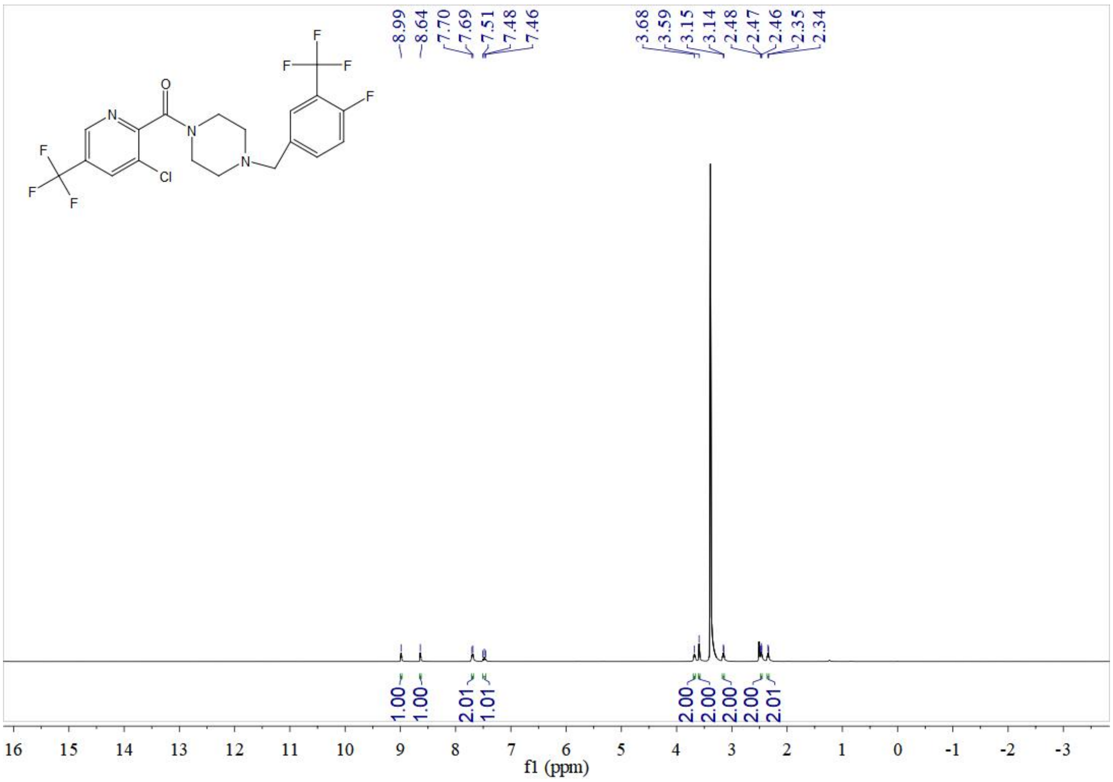

A3 ^{13}C NMR

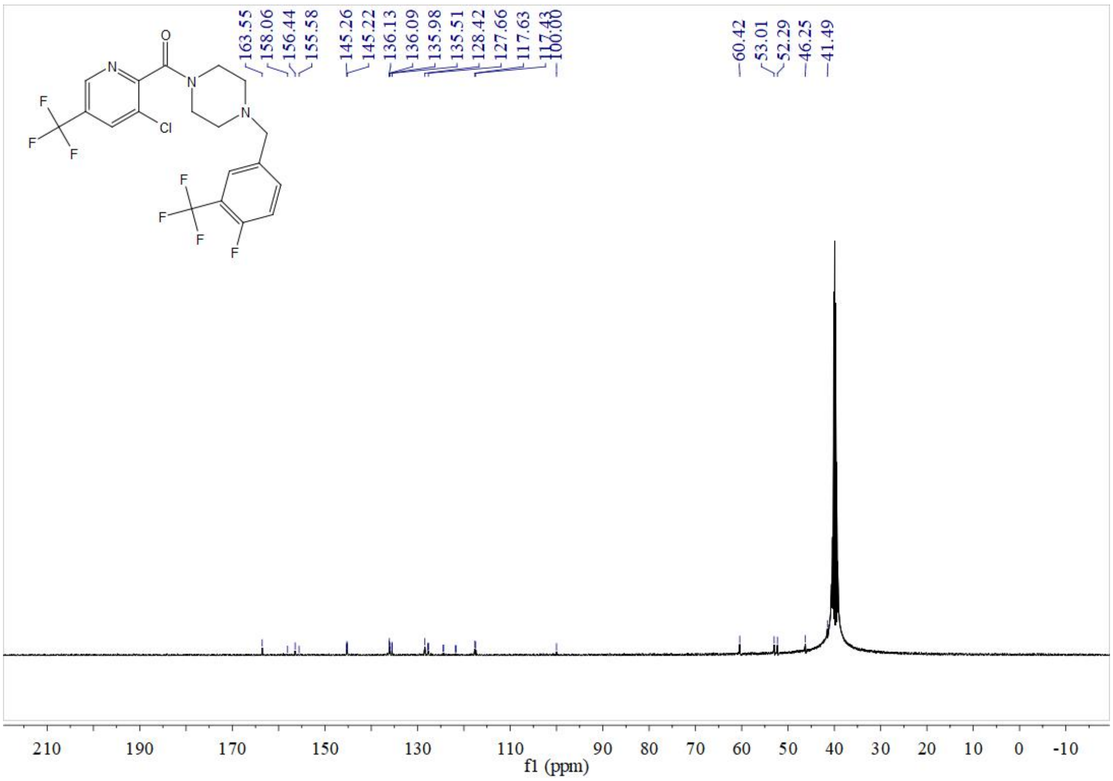

A3 ^{19}F NMR

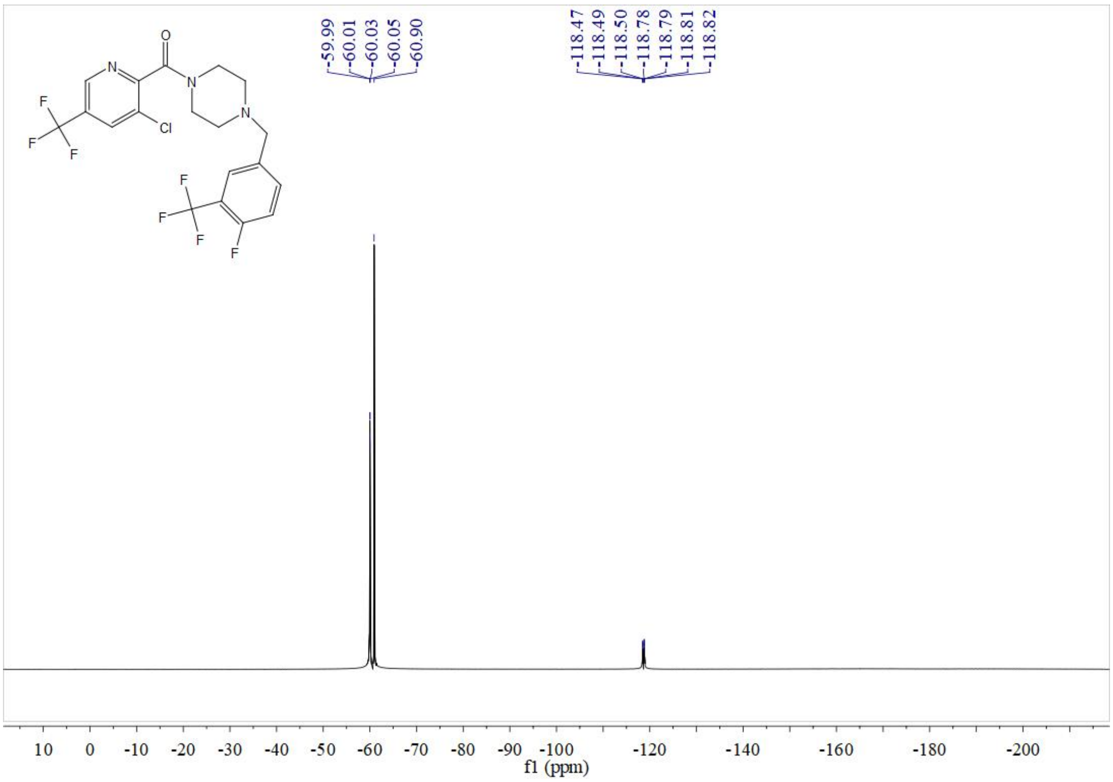

A3 HRMS

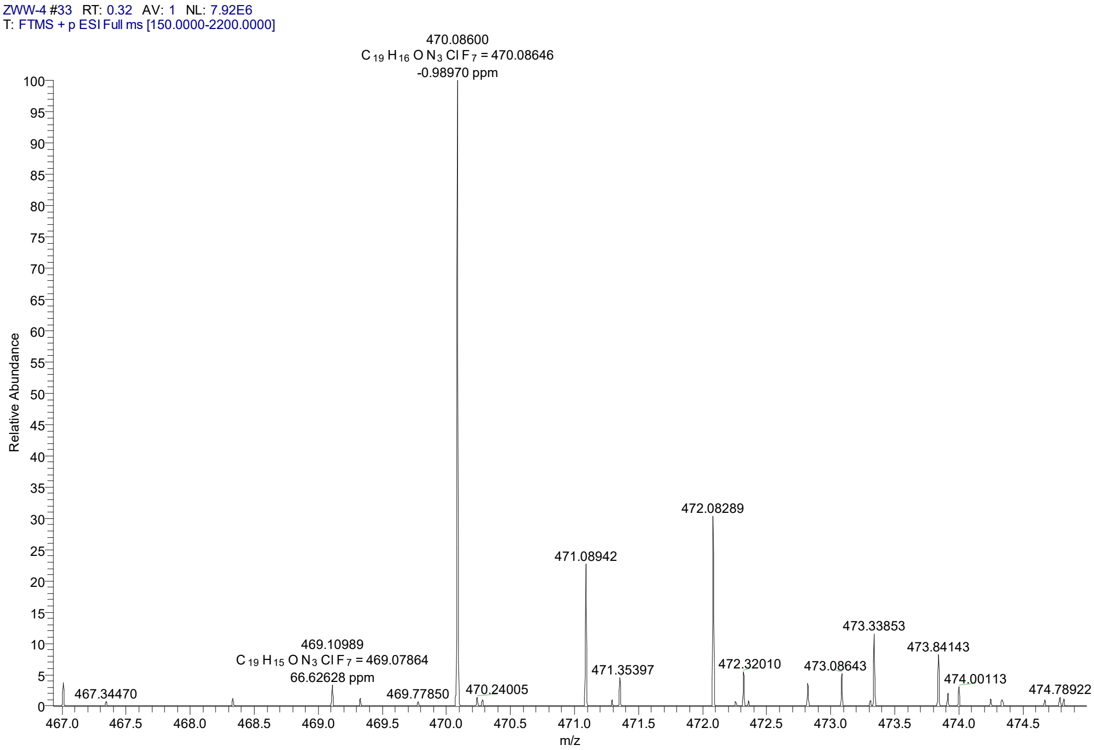

A4 ^{1}H NMR

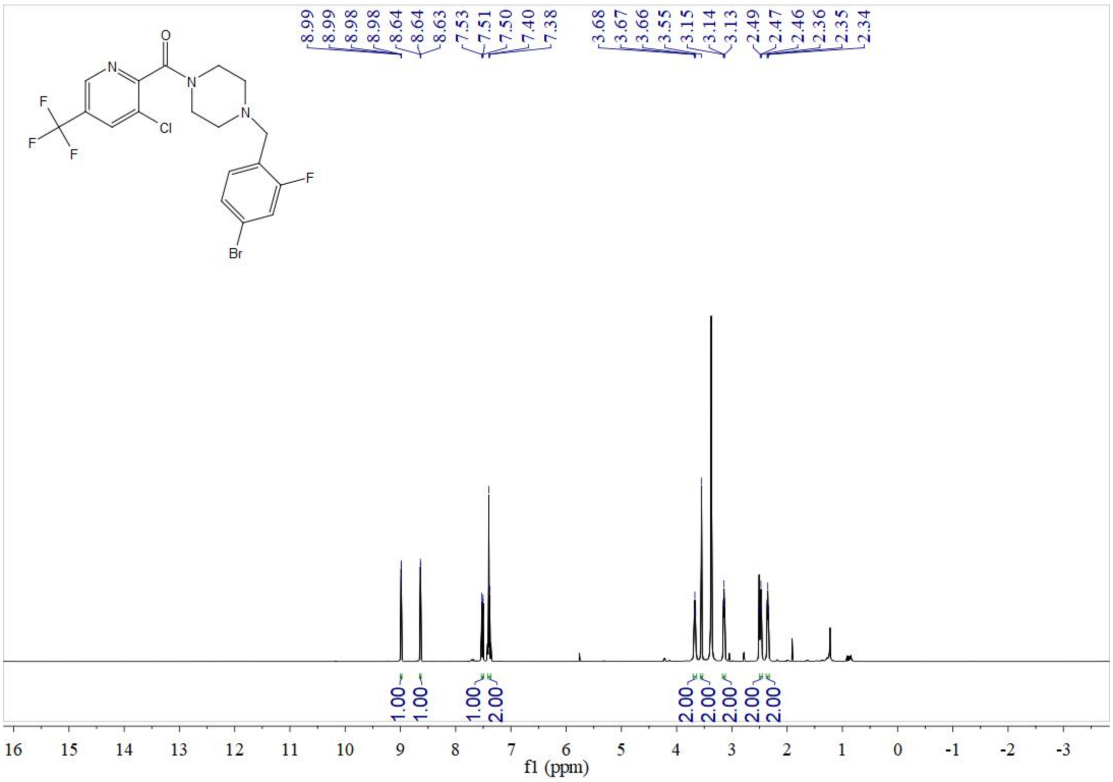

A4 ^{13}C NMR

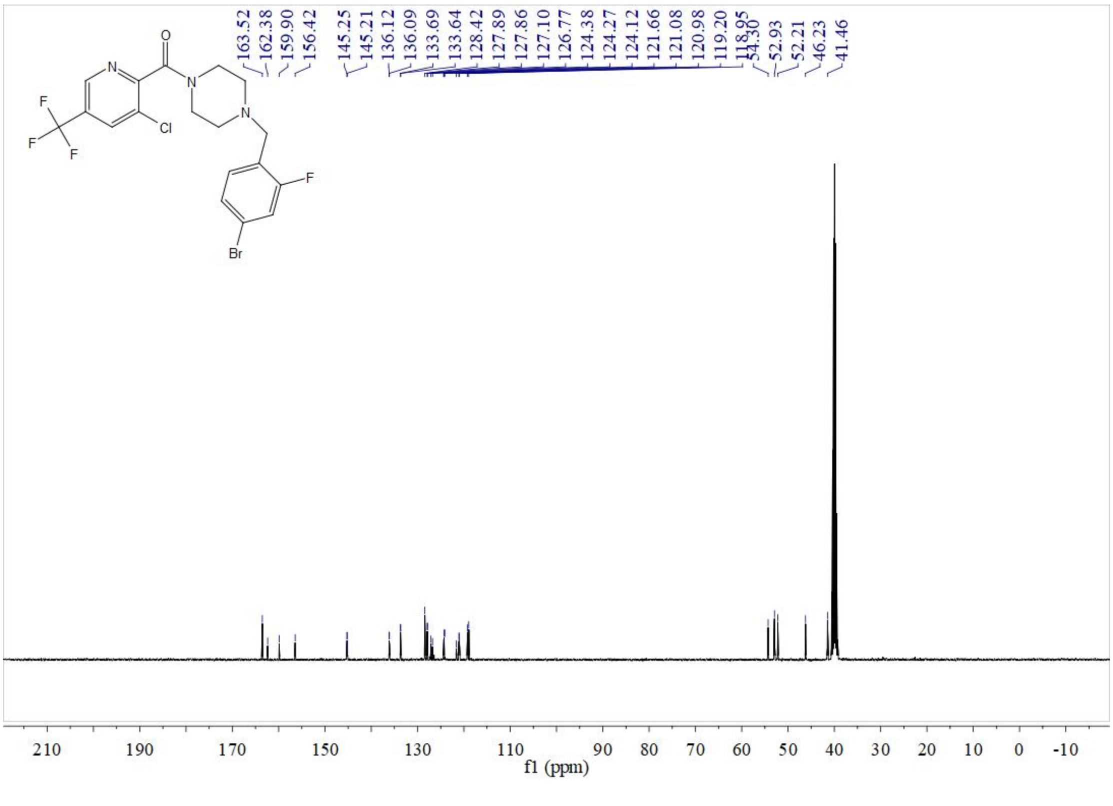

A4 ^{19}F NMR

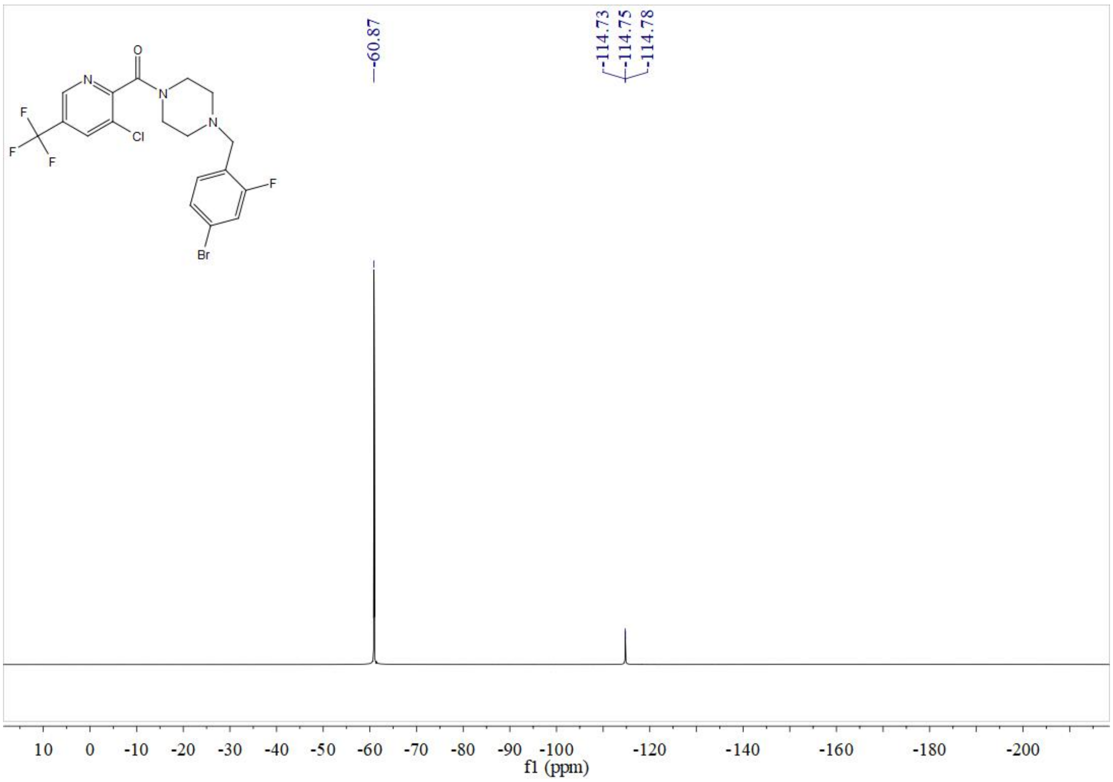

A4 HRMS

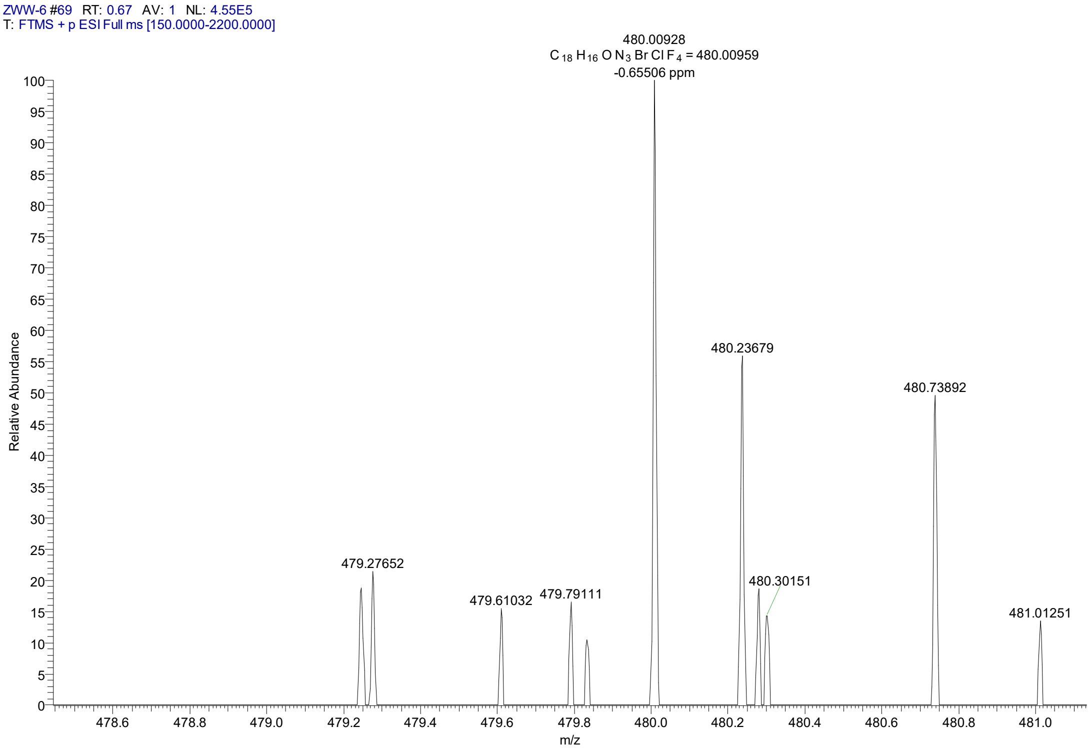

A5 ^{1}H NMR

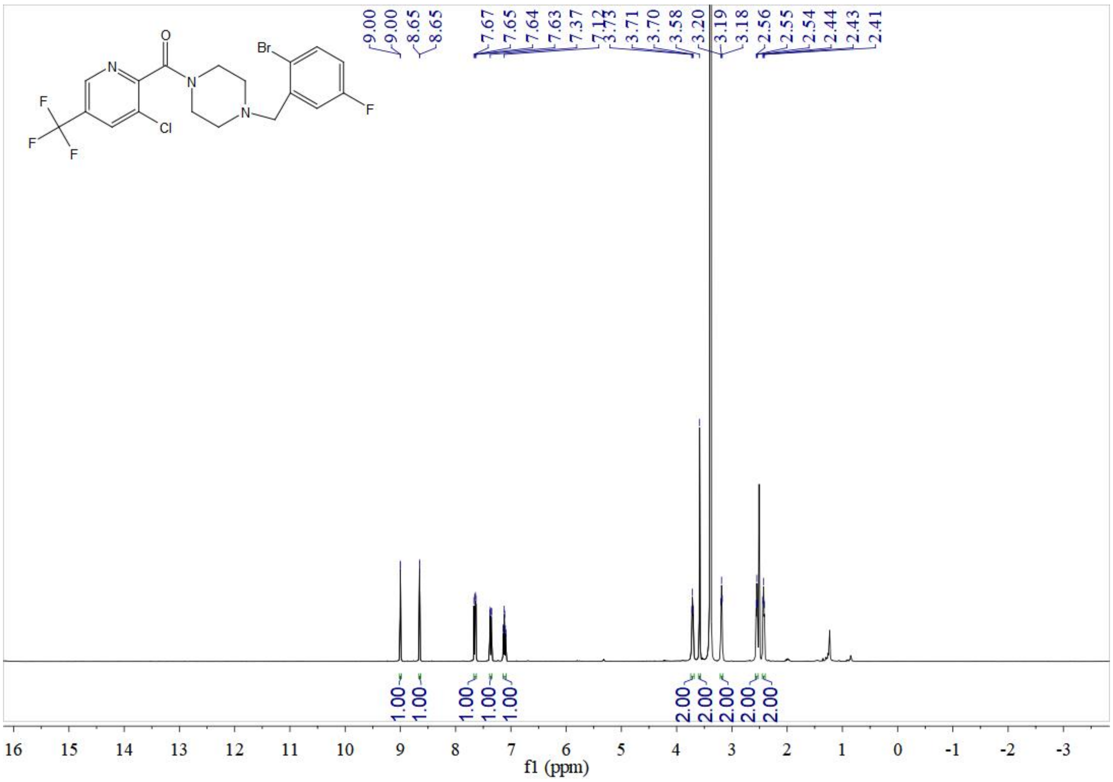

A5 ^{13}C NMR

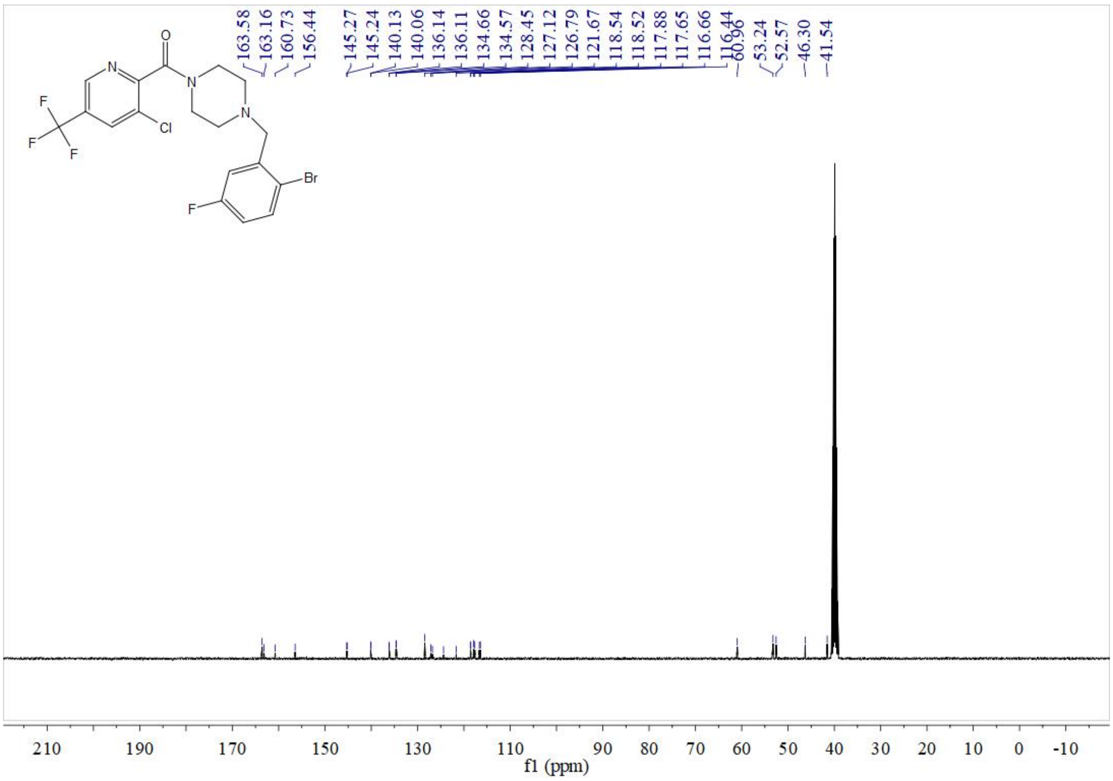

A5 ^{19}F NMR

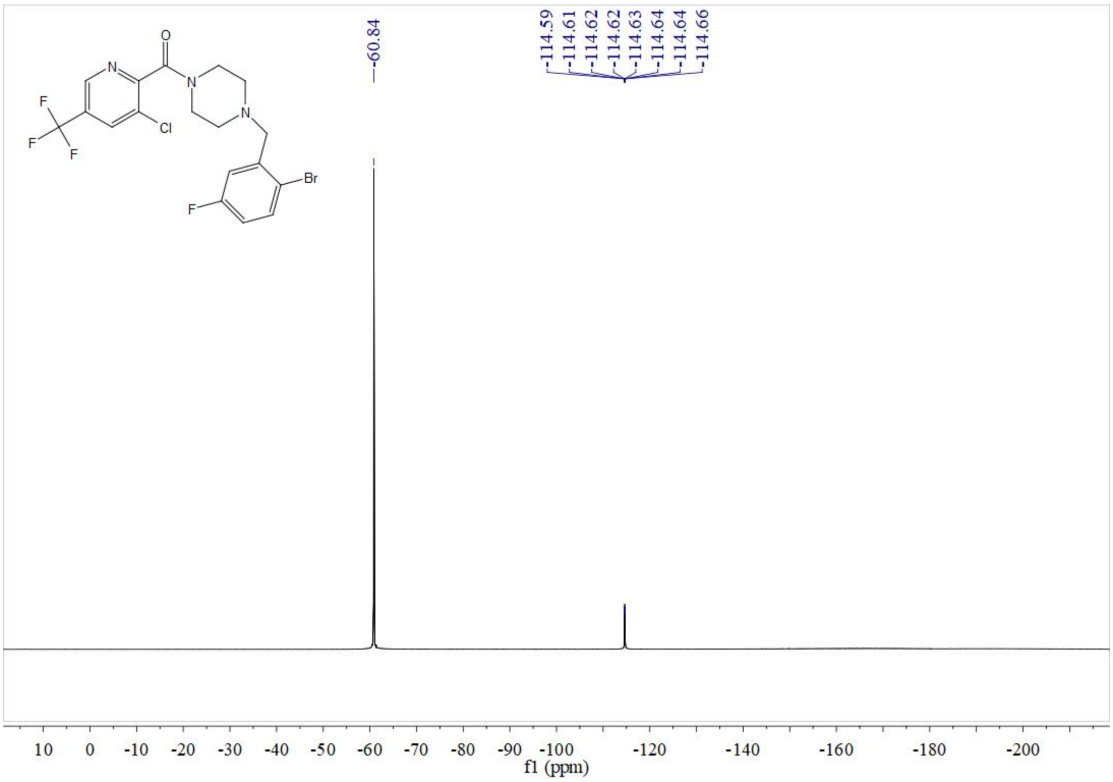

A5 HRMS

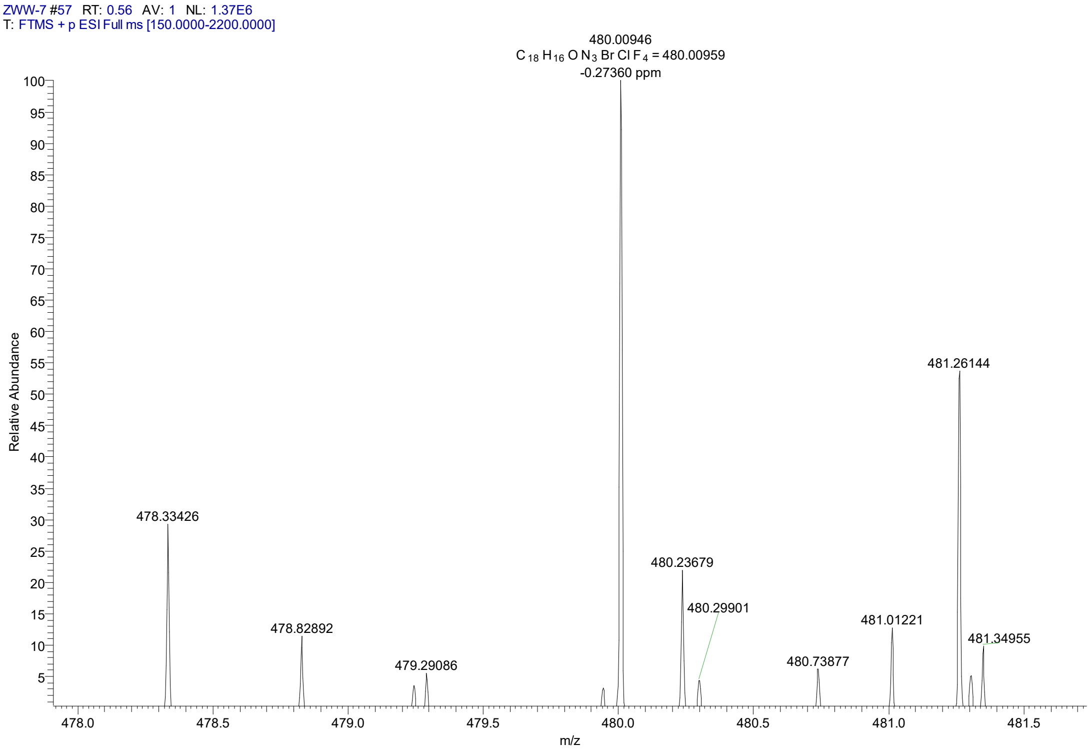

A6 ^{1}H NMR

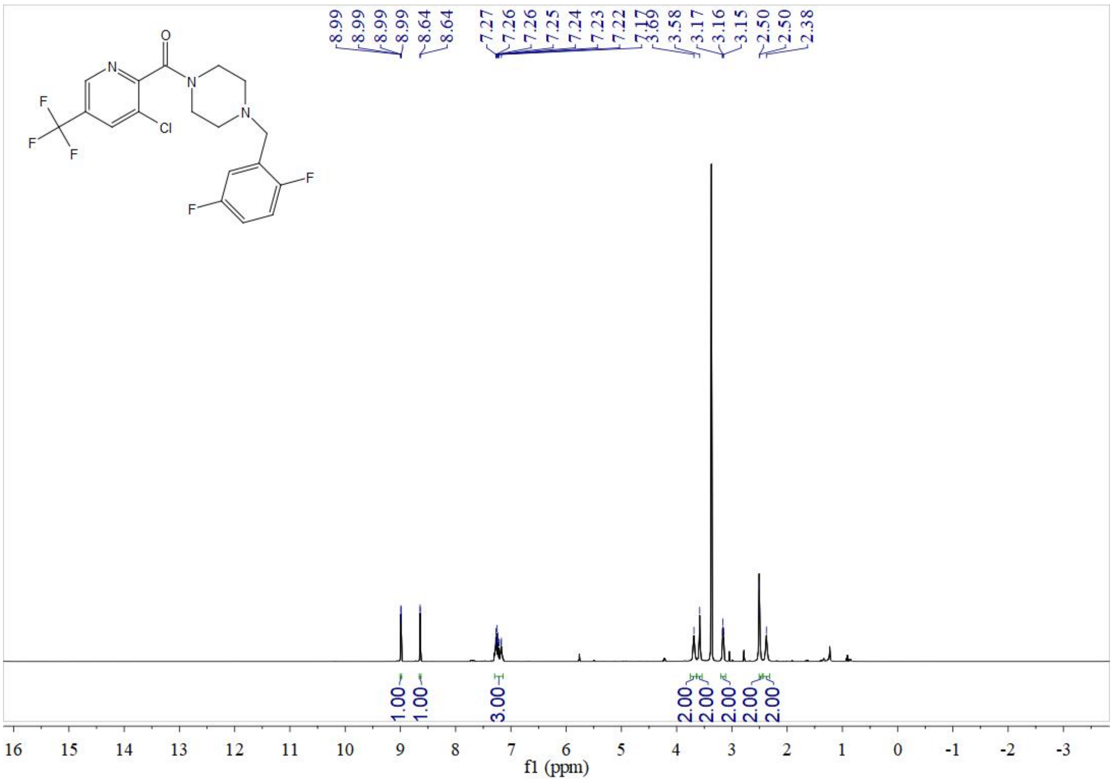

A6 ^{13}C NMR

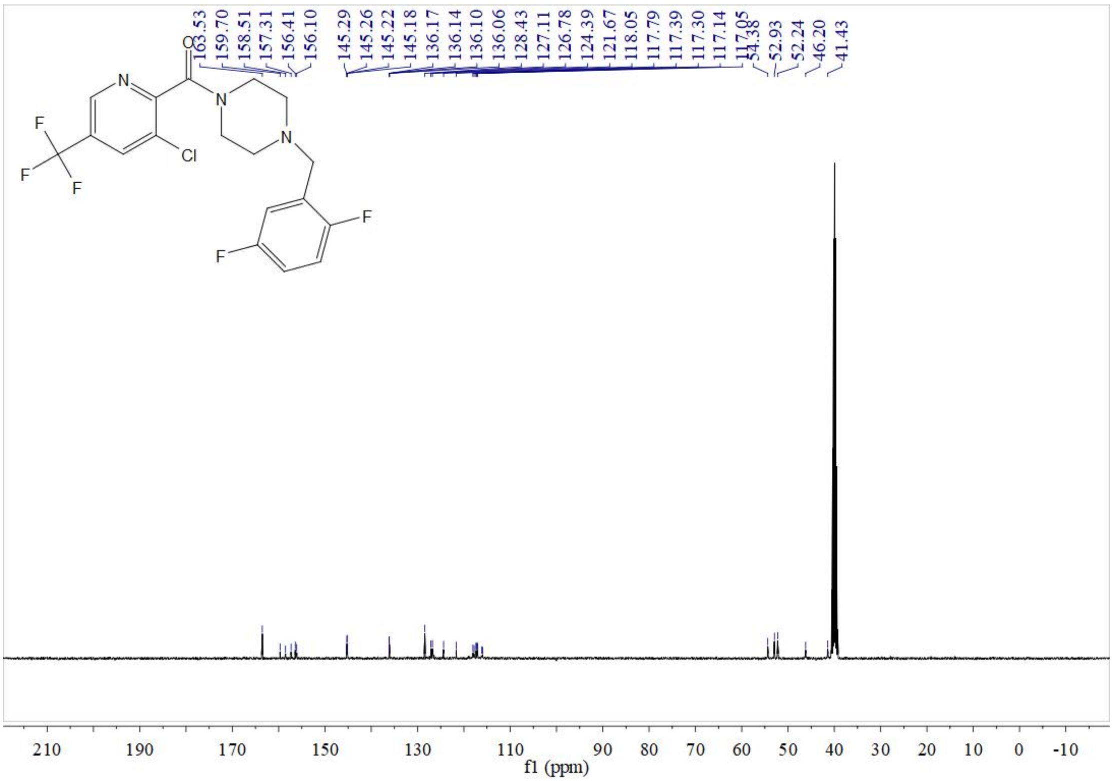

A6 ^{19}F NMR

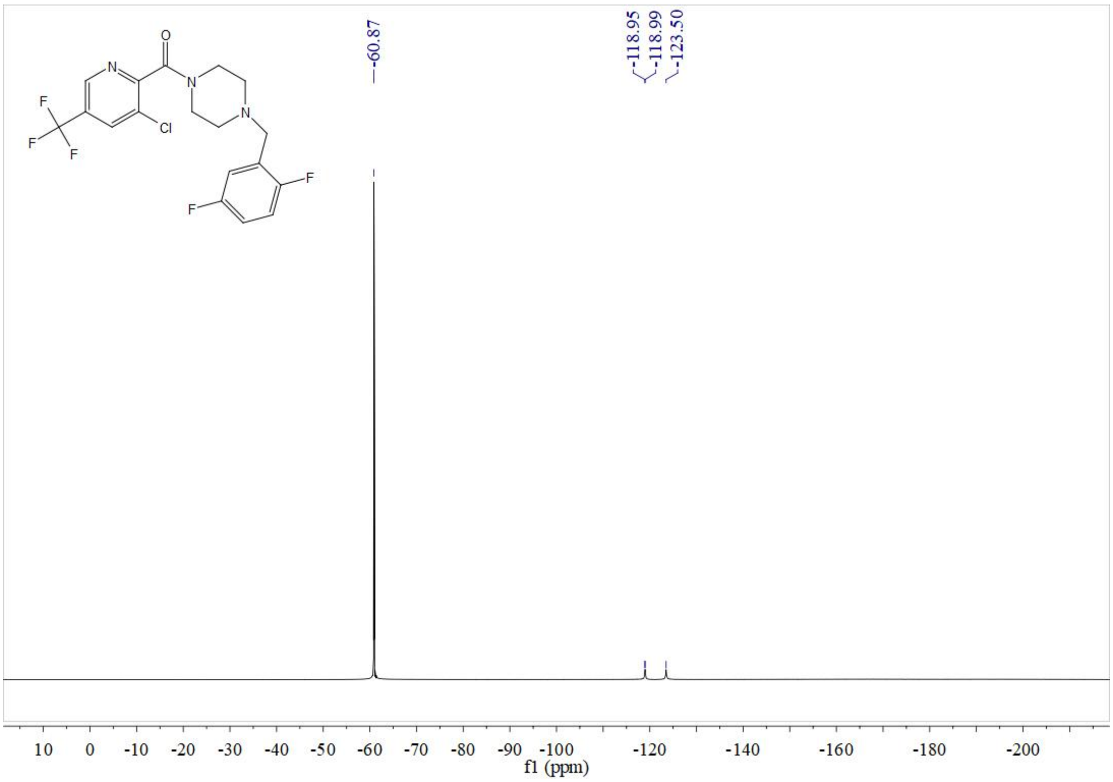

A6 HRMS

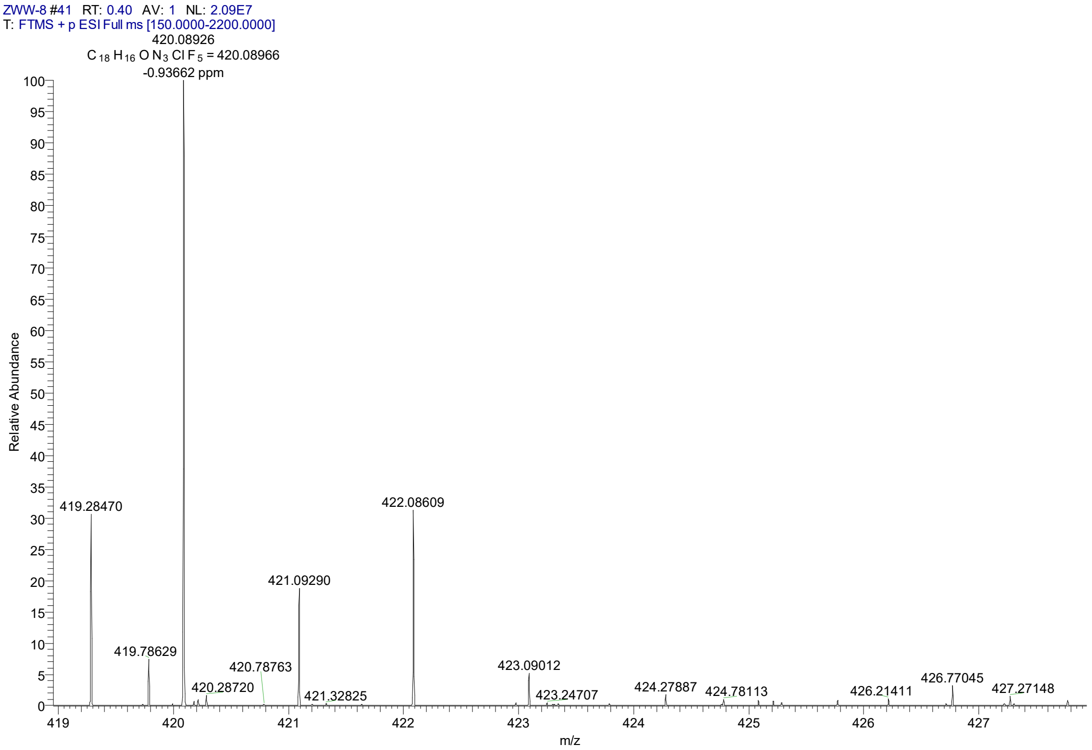

A7 ^{1}H NMR

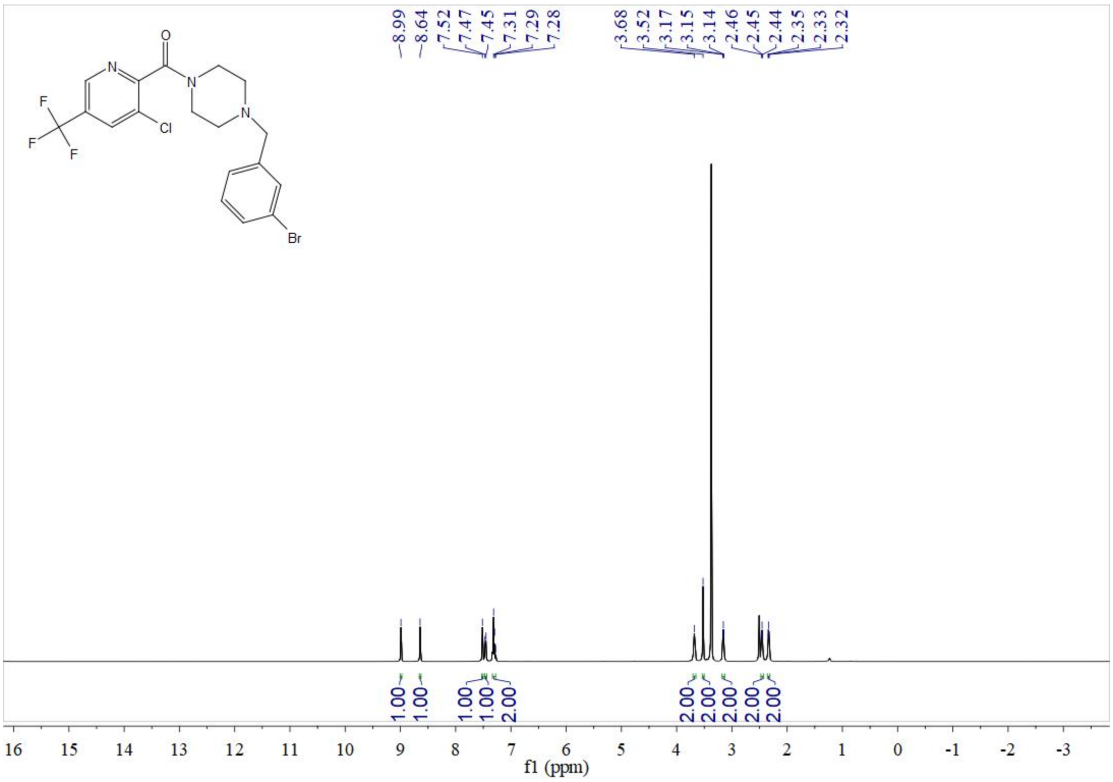

A7 ^{13}C NMR

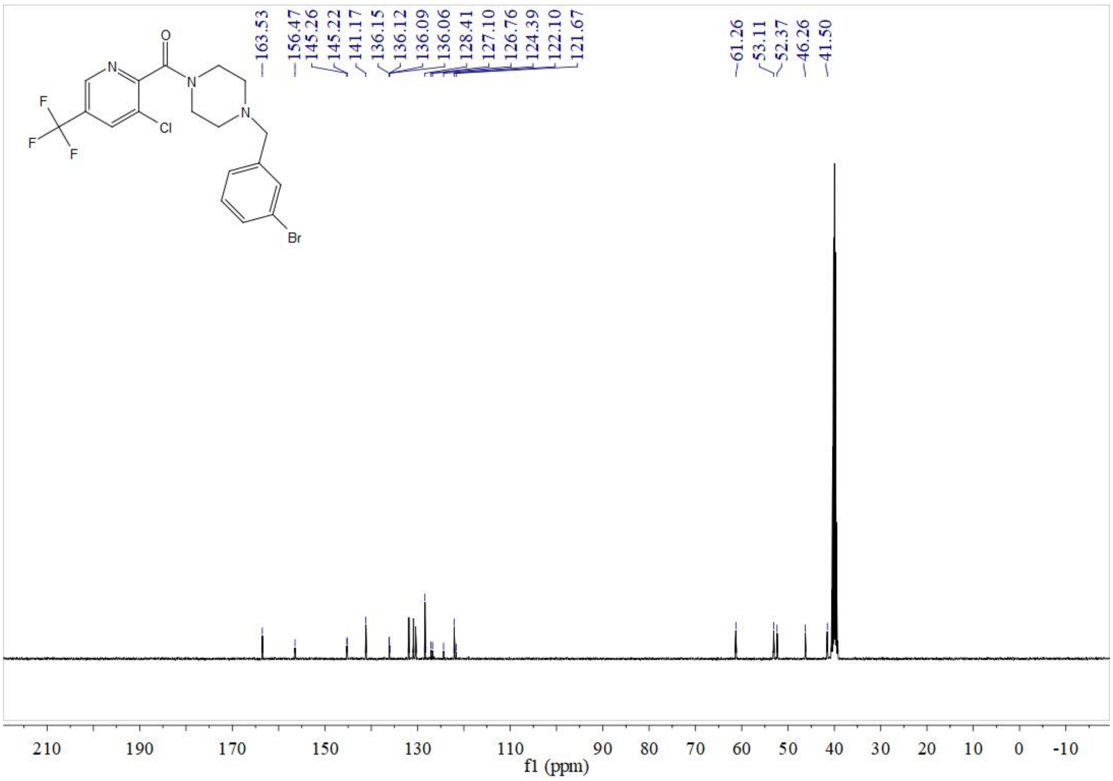

A7 ^{19}F NMR

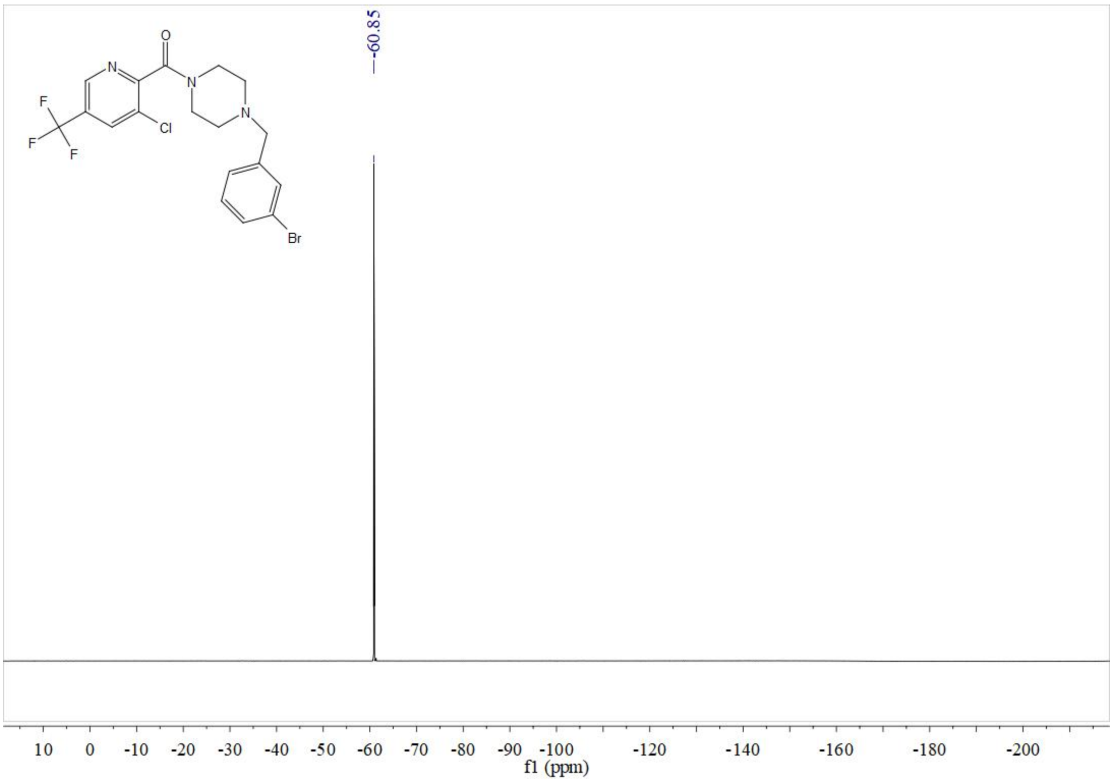

A7 HRMS

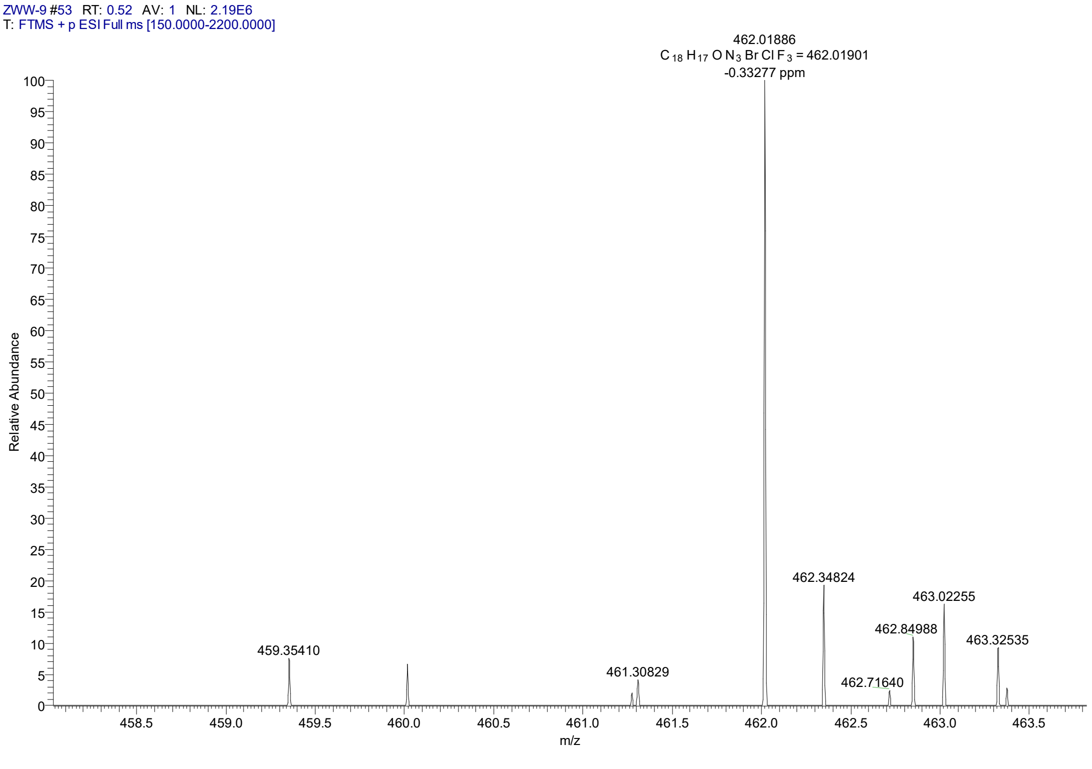

A8 ^{1}H NMR

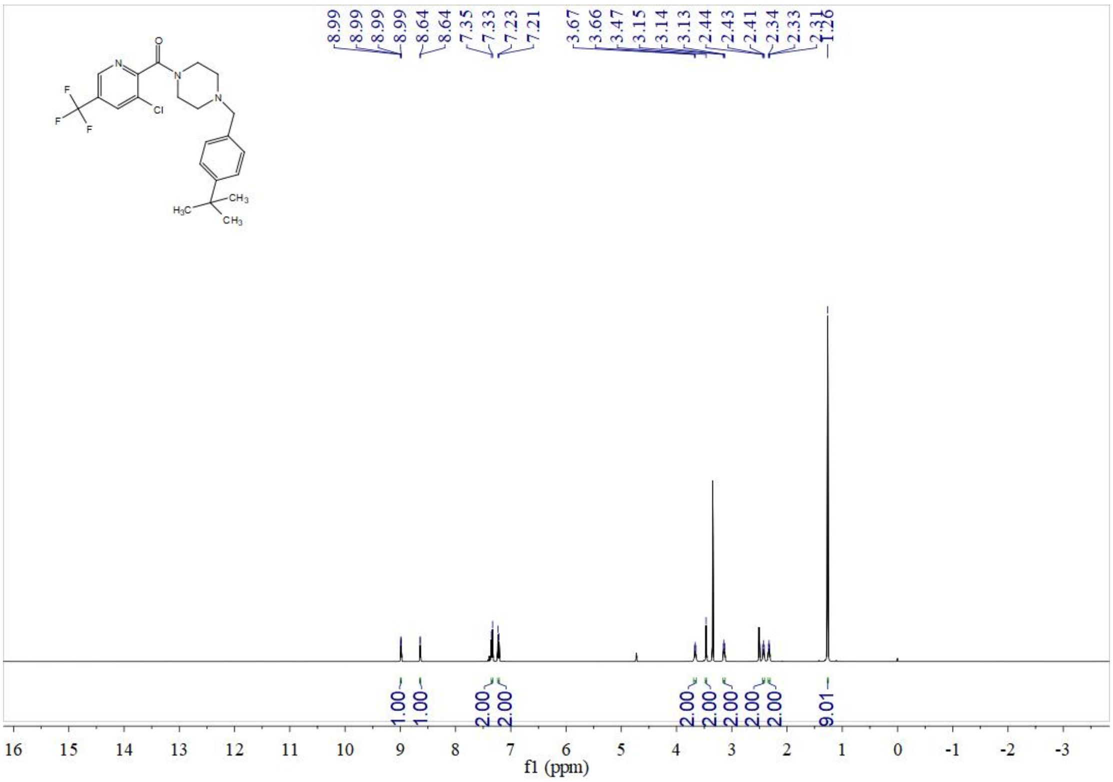

A8 ^{13}C NMR

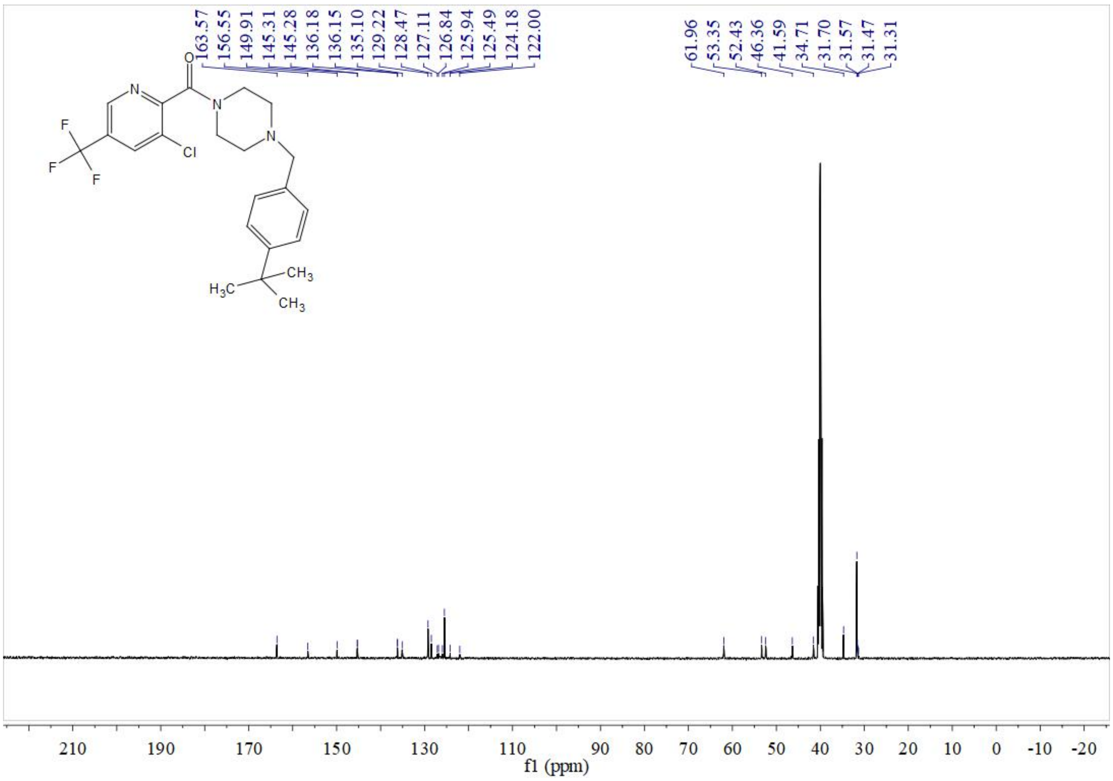

A8 ^{19}F NMR

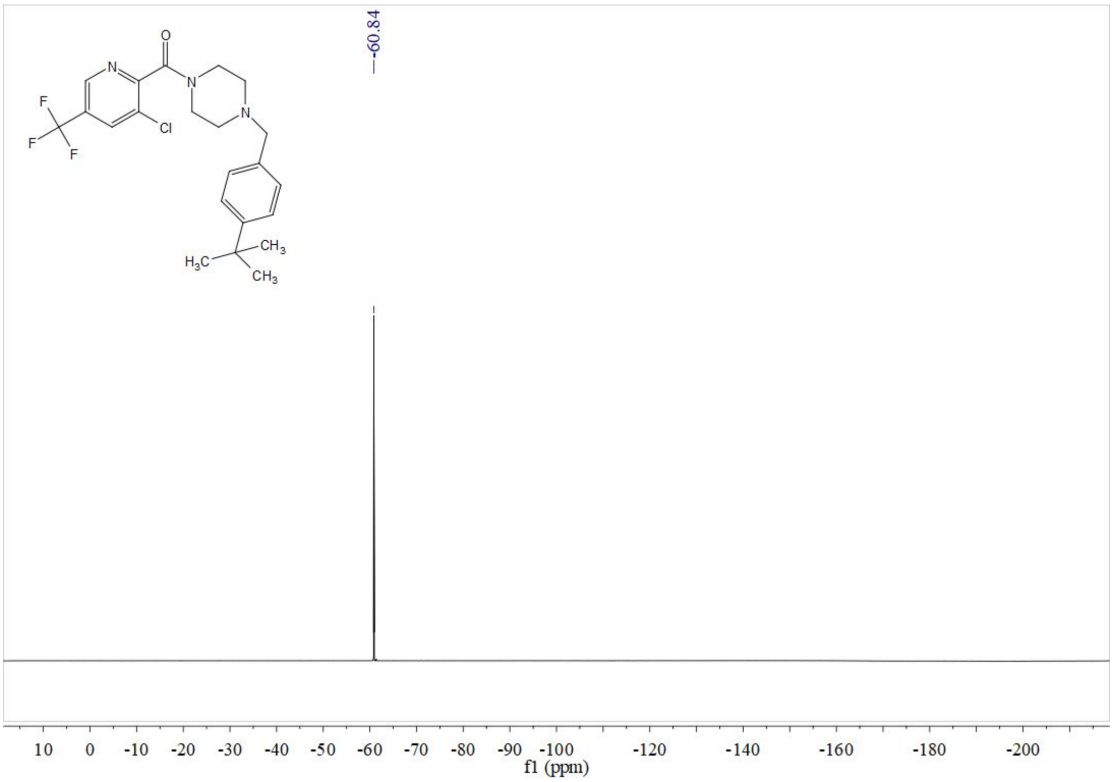

A8 HRMS

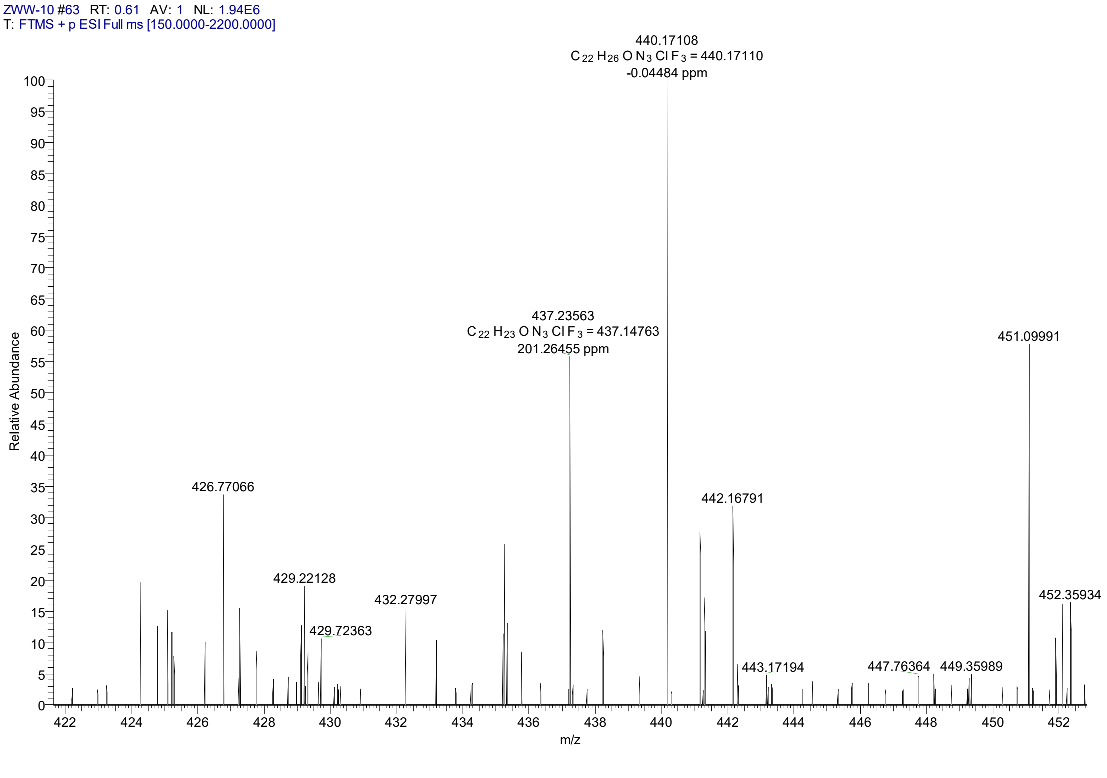

A9 ^{1}H NMR

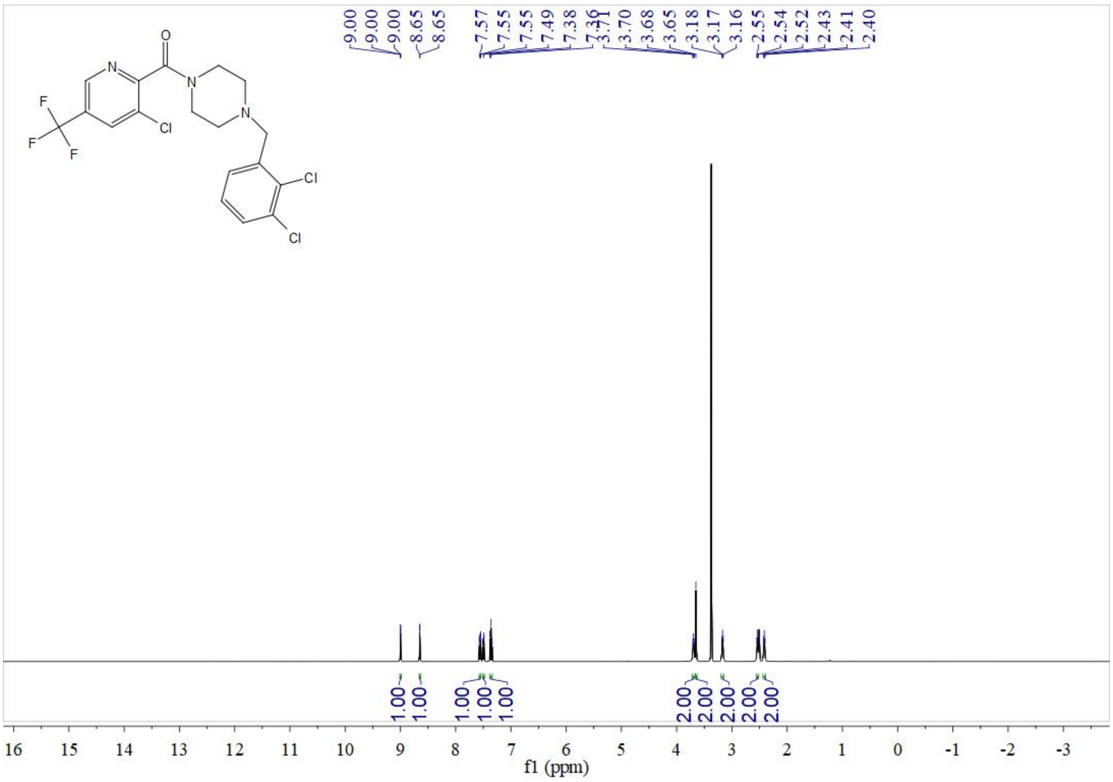

A9 ^{13}C NMR

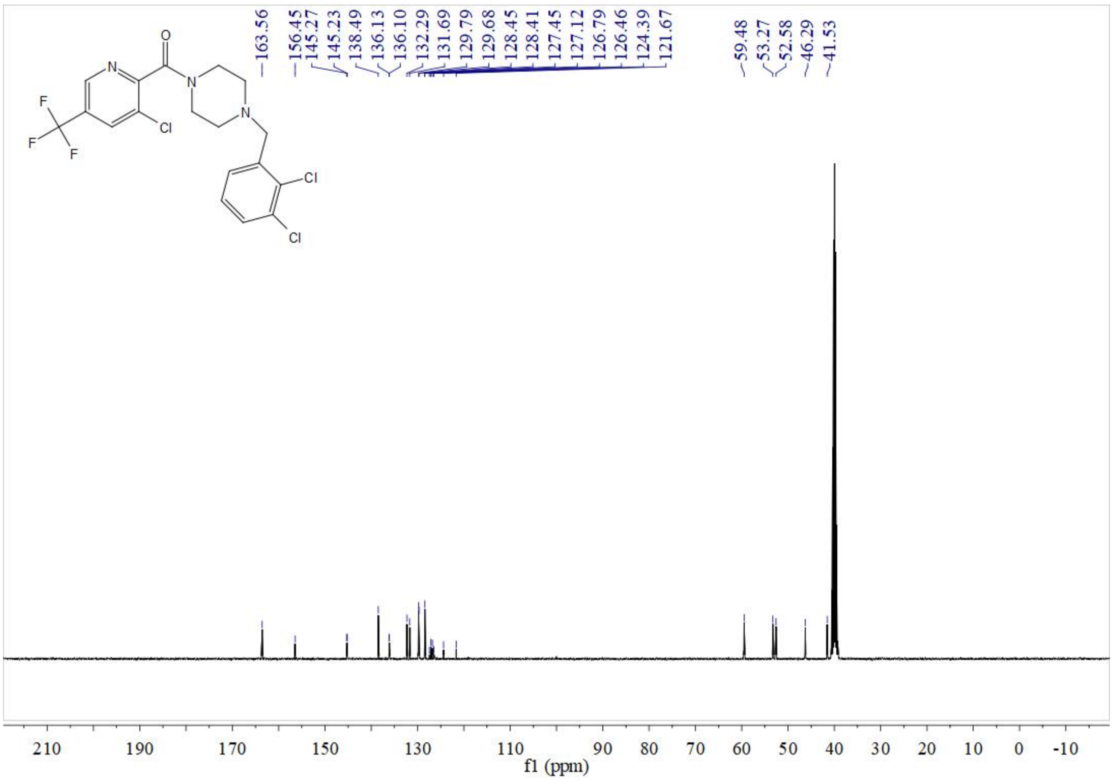

A9 ^{19}F NMR

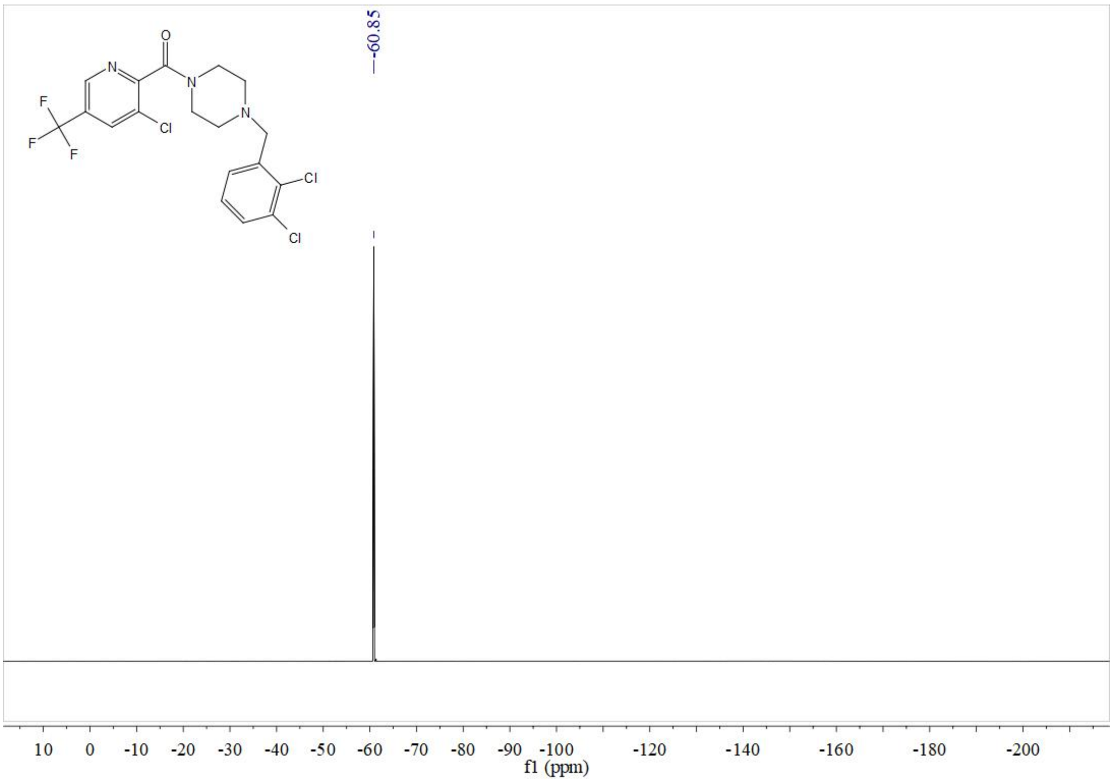

A9 HRMS

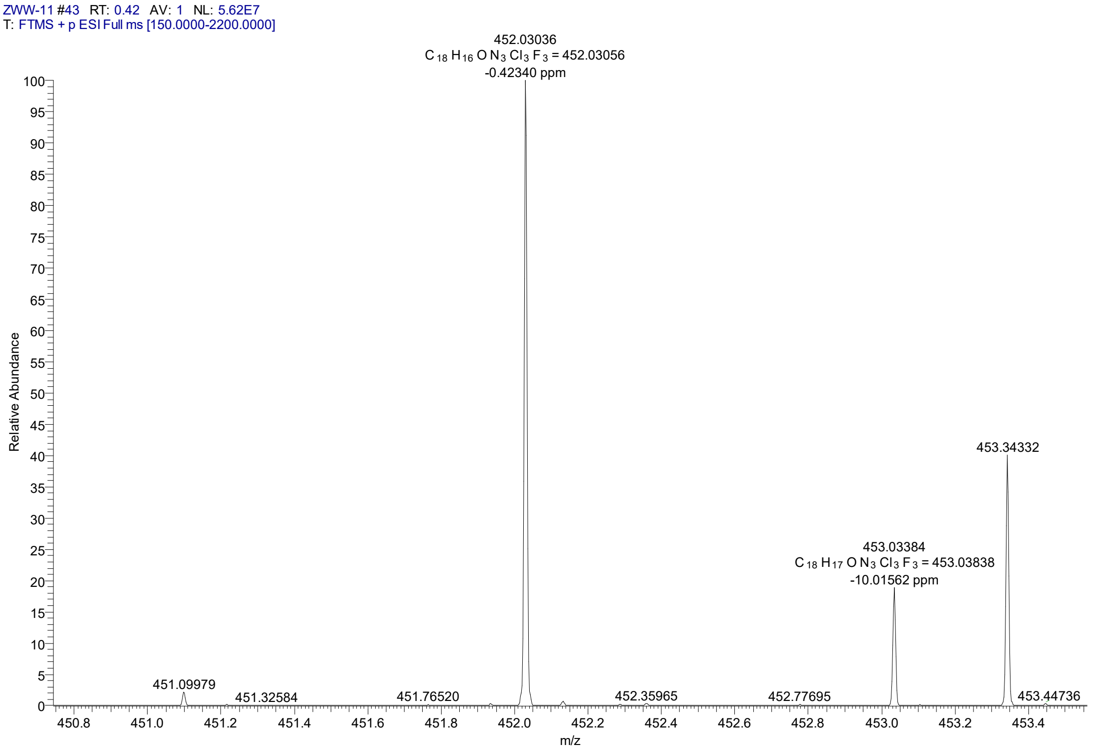

A10 ^{1}H NMR

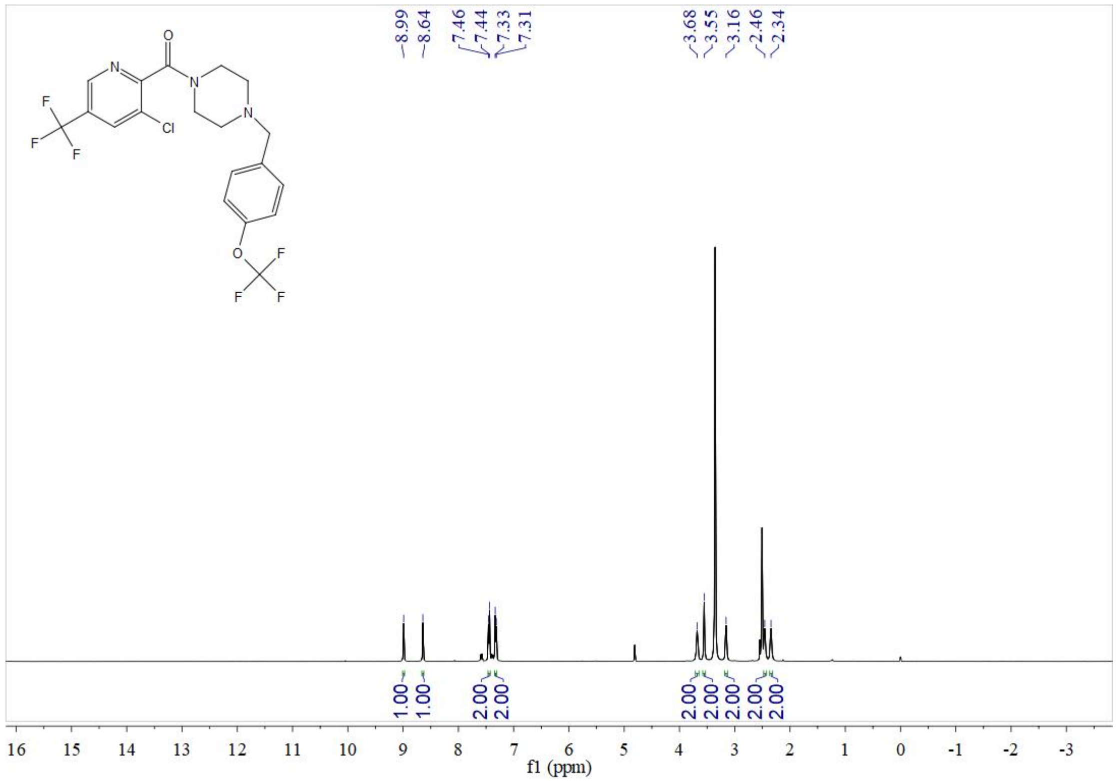

A10 ^{13}C NMR

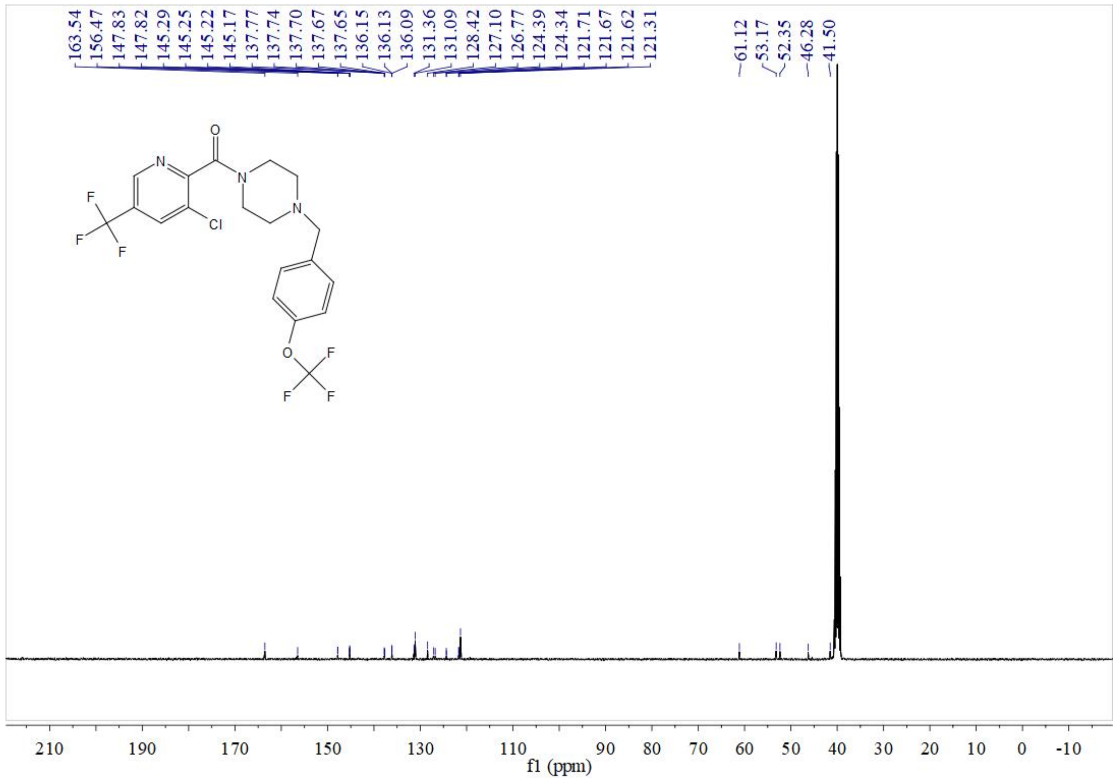

A10 ^{19}F NMR

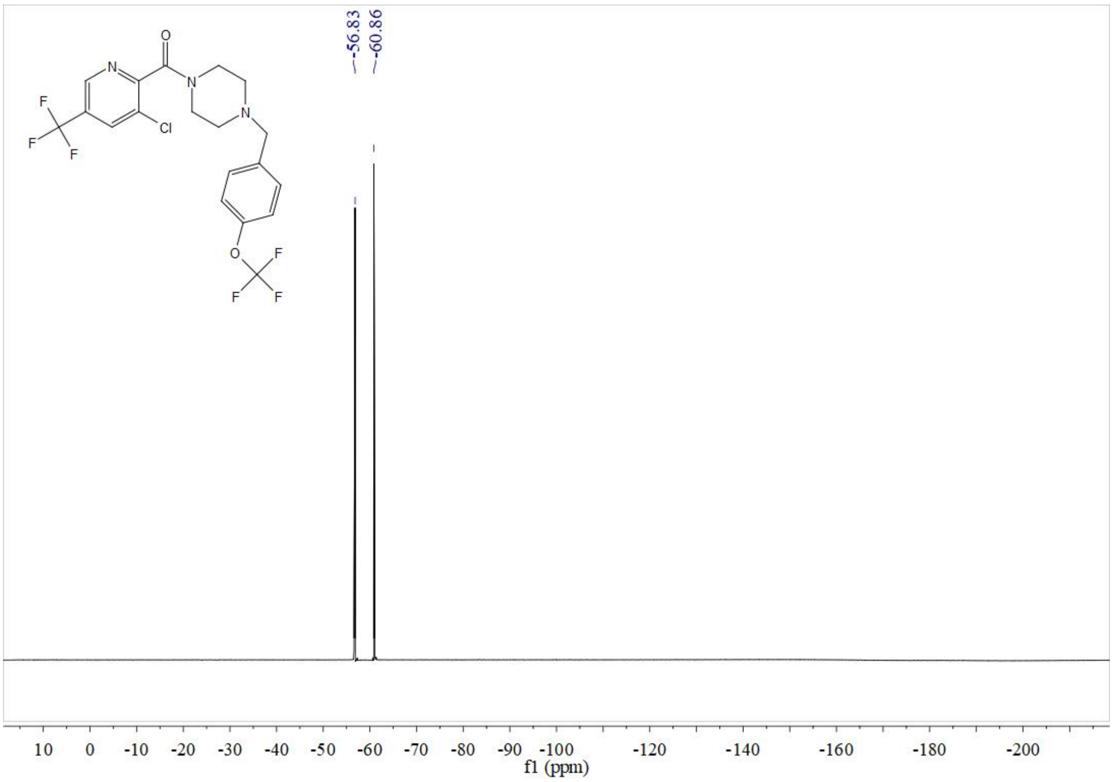

A10 HRMS

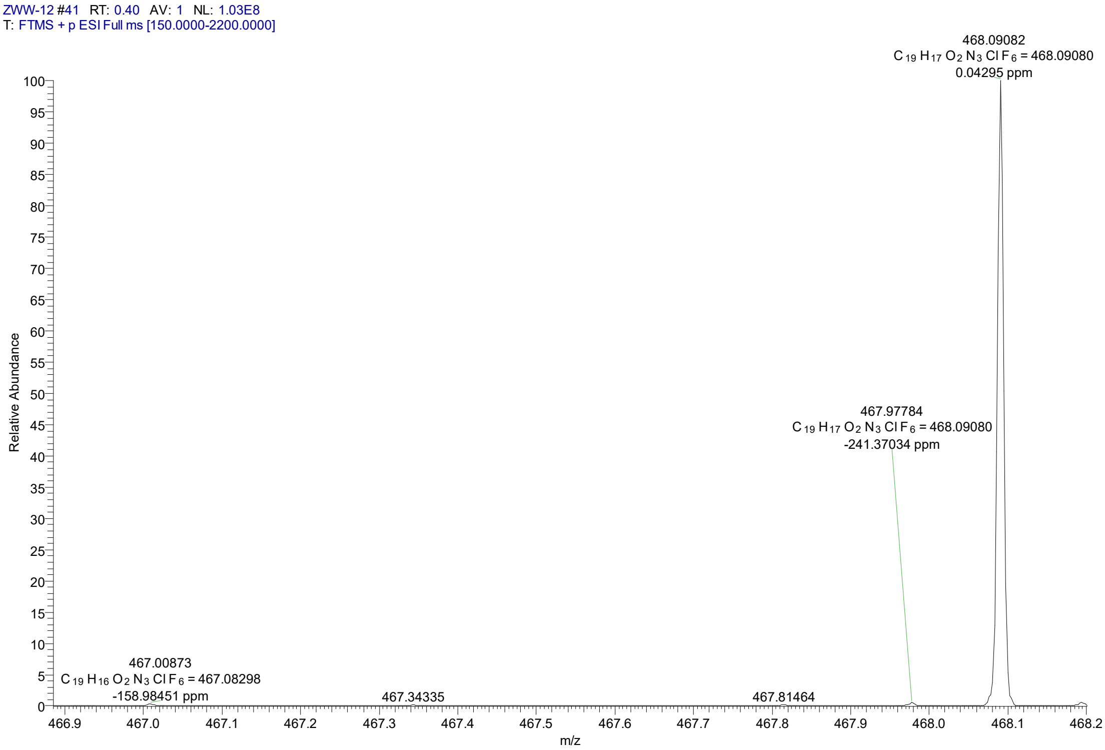

A11 ^{1}H NMR

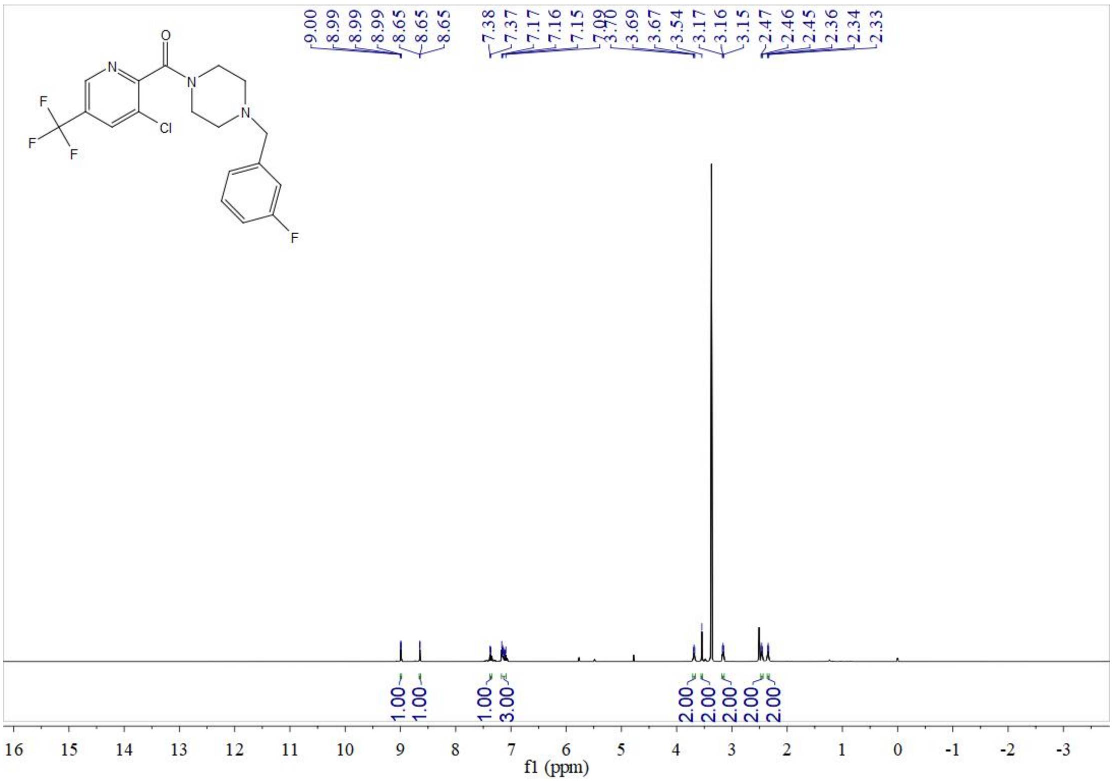

A11 ^{13}C NMR

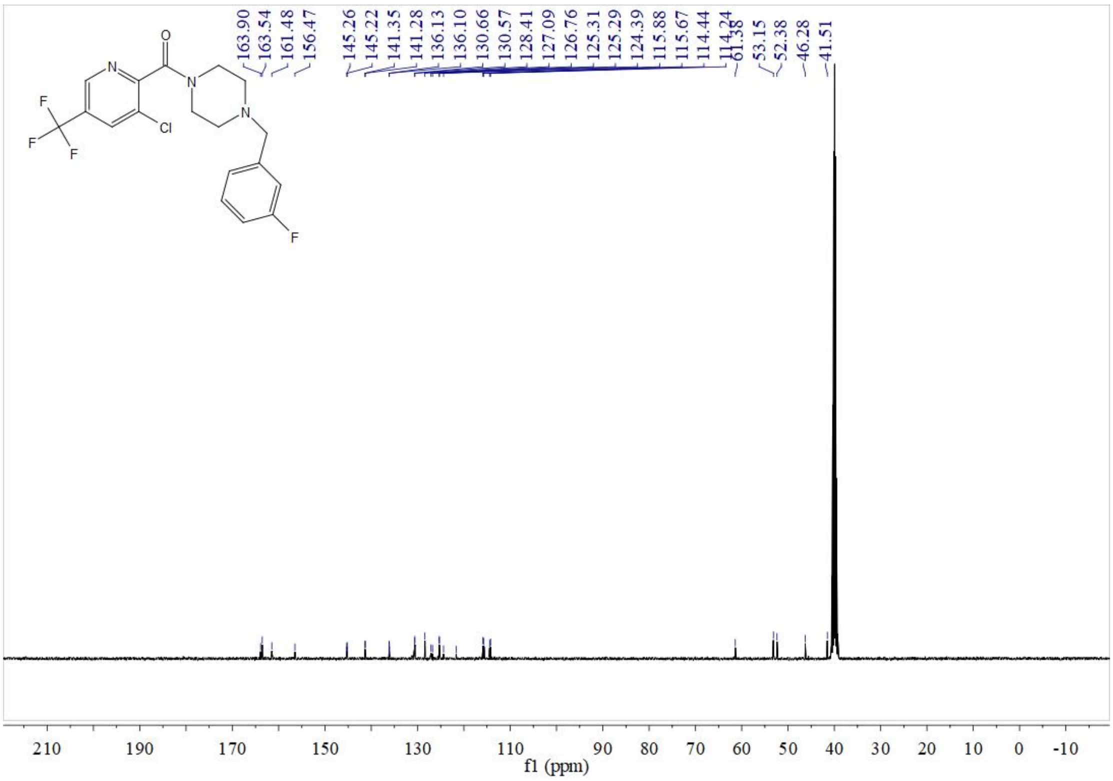

A11 ^{19}F NMR

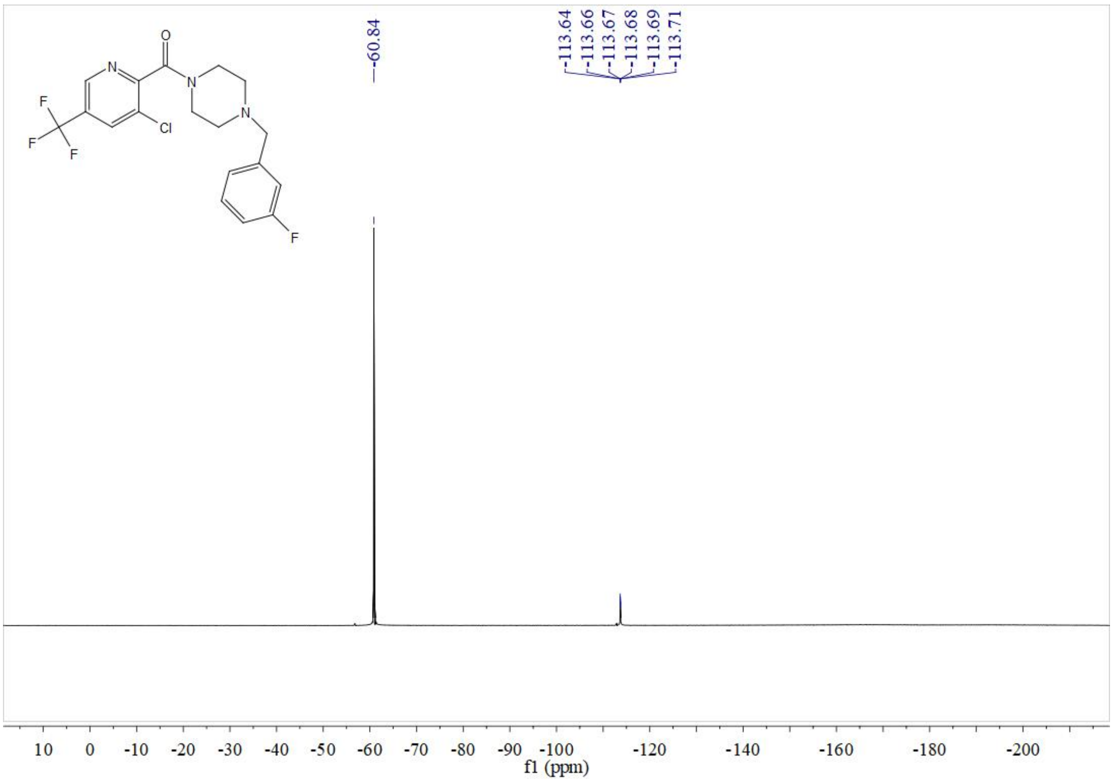

A11 HRMS

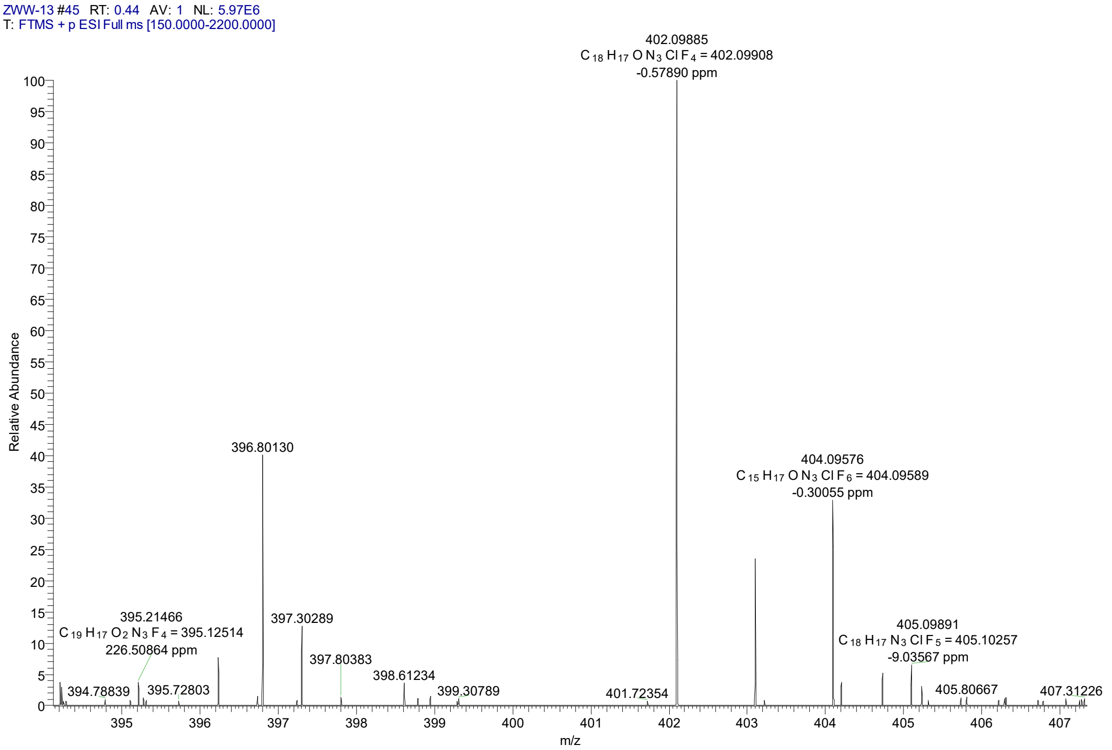

A12 ^{1}H NMR

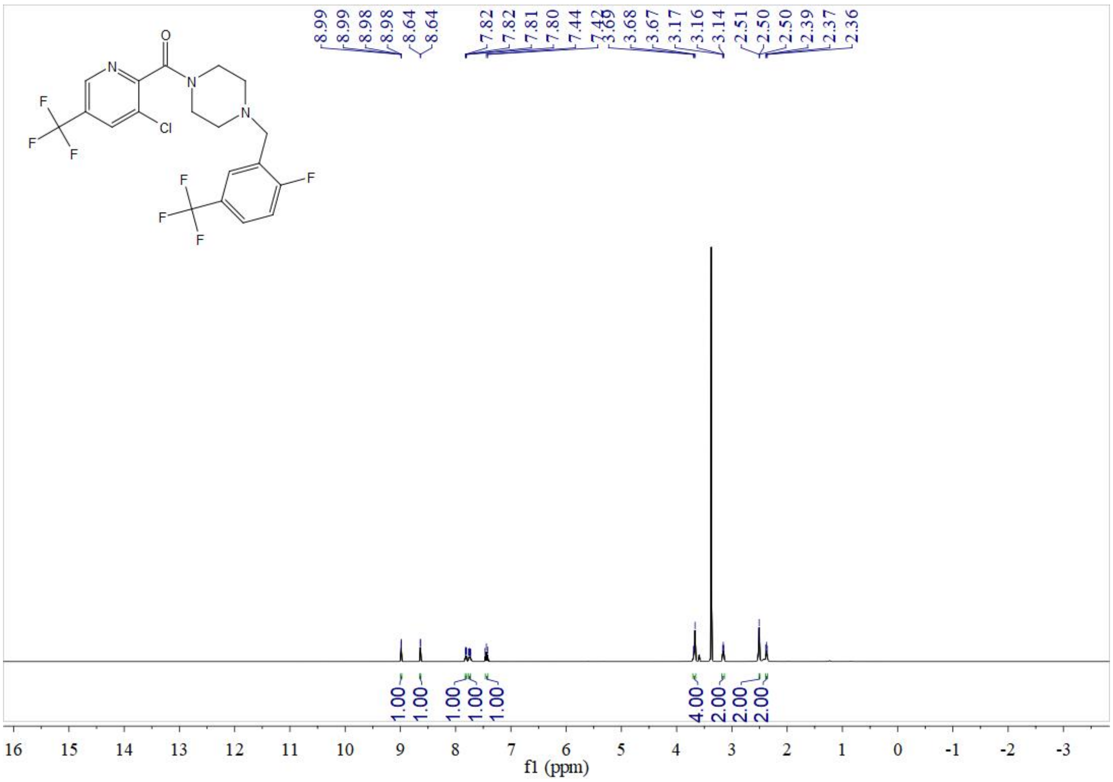

A12 ^{13}C NMR

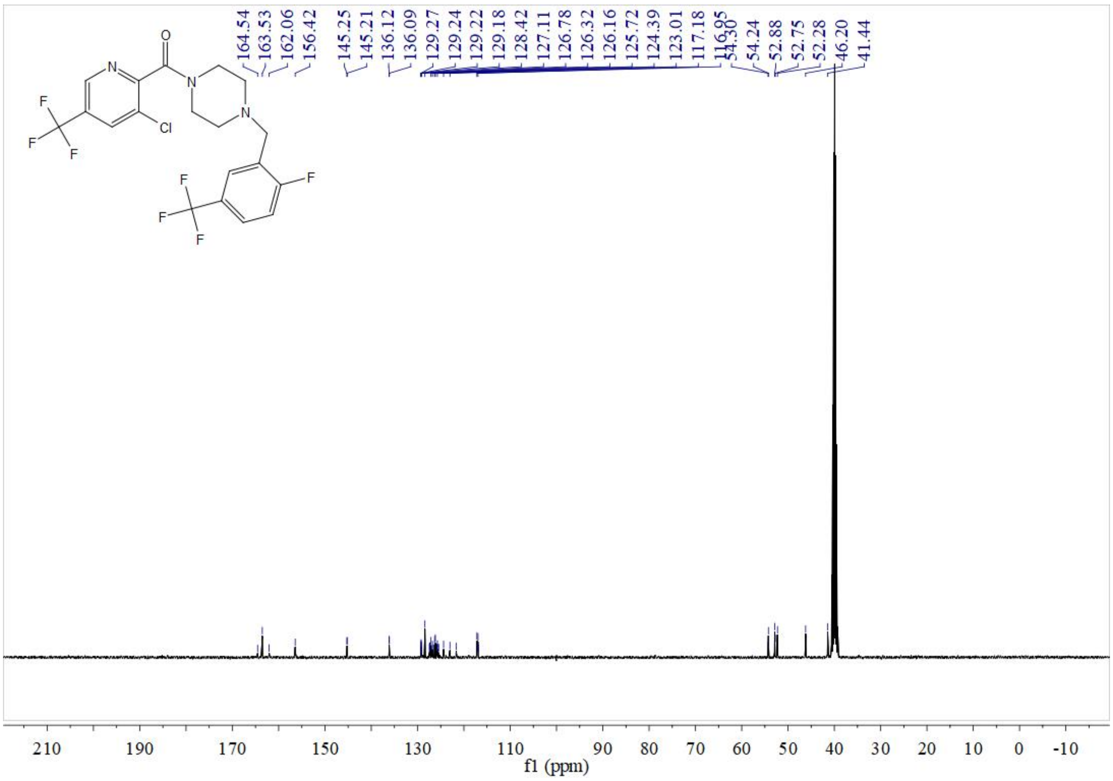

A12 ^{19}F NMR

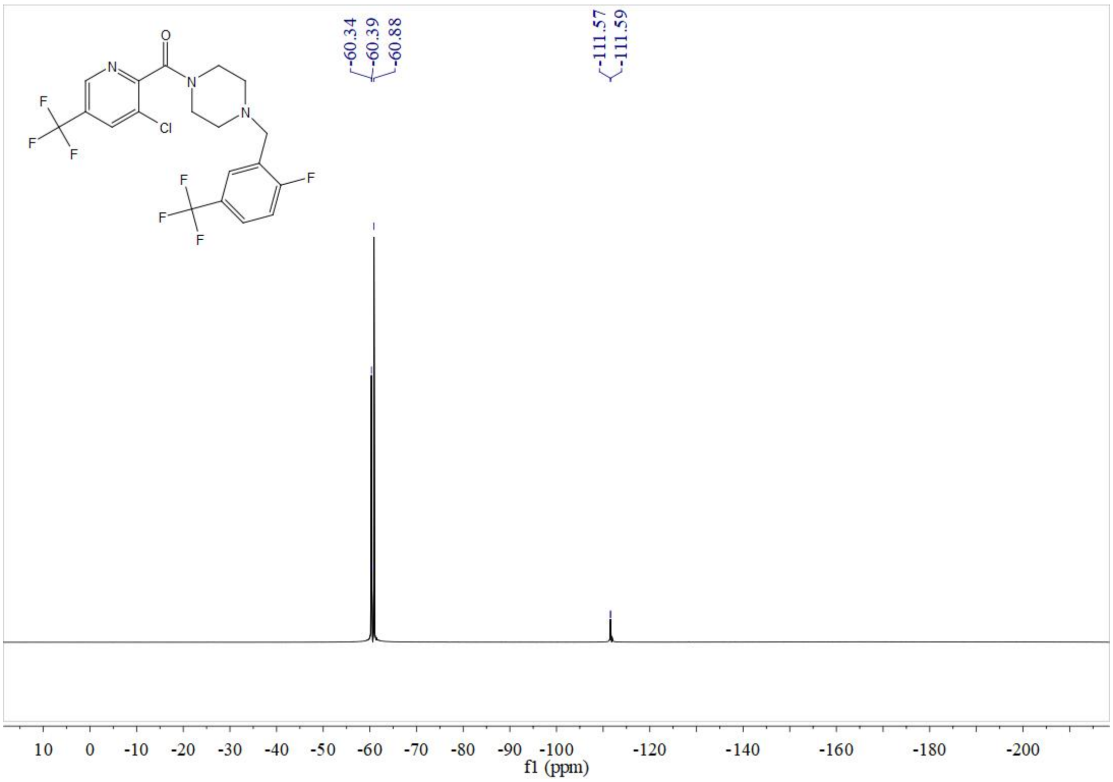

A12 HRMS

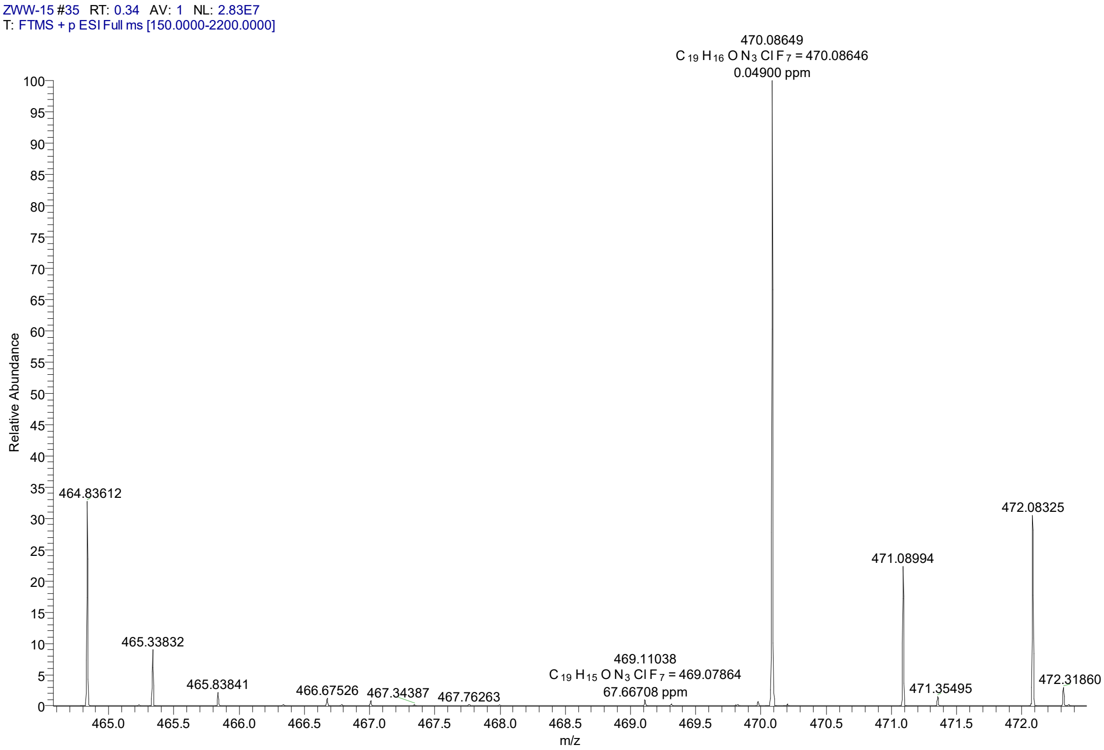

A13 ¹H NMR

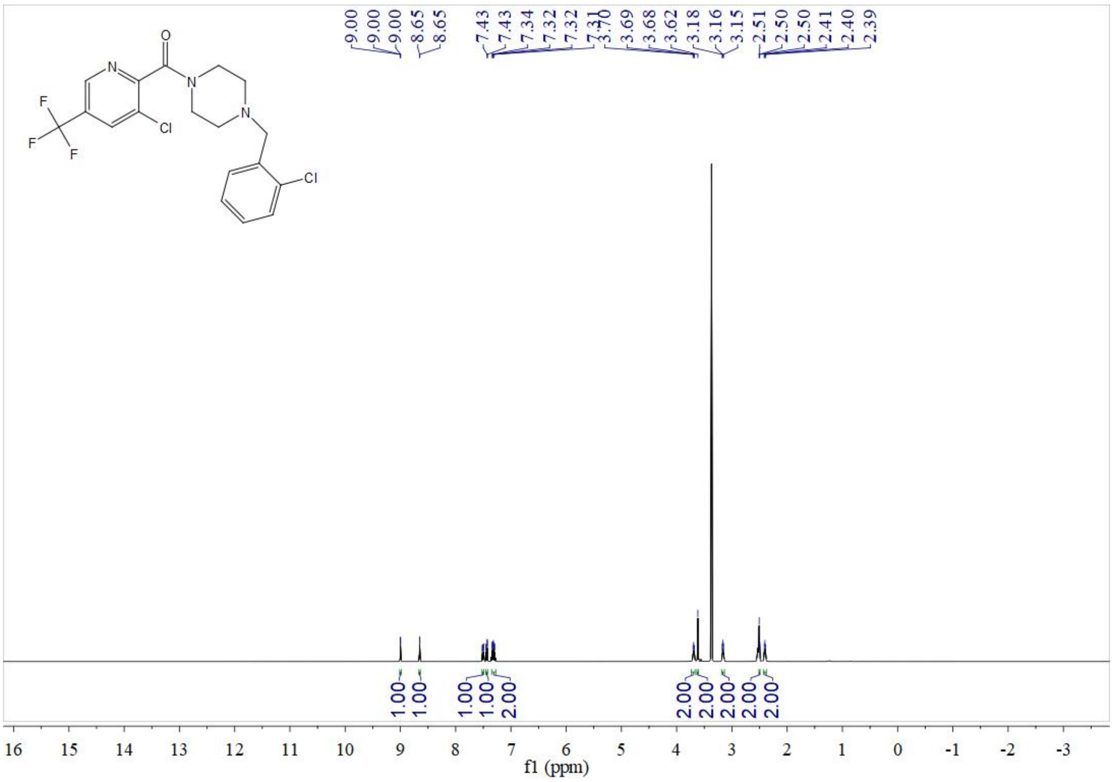

A13 ¹³C NMR

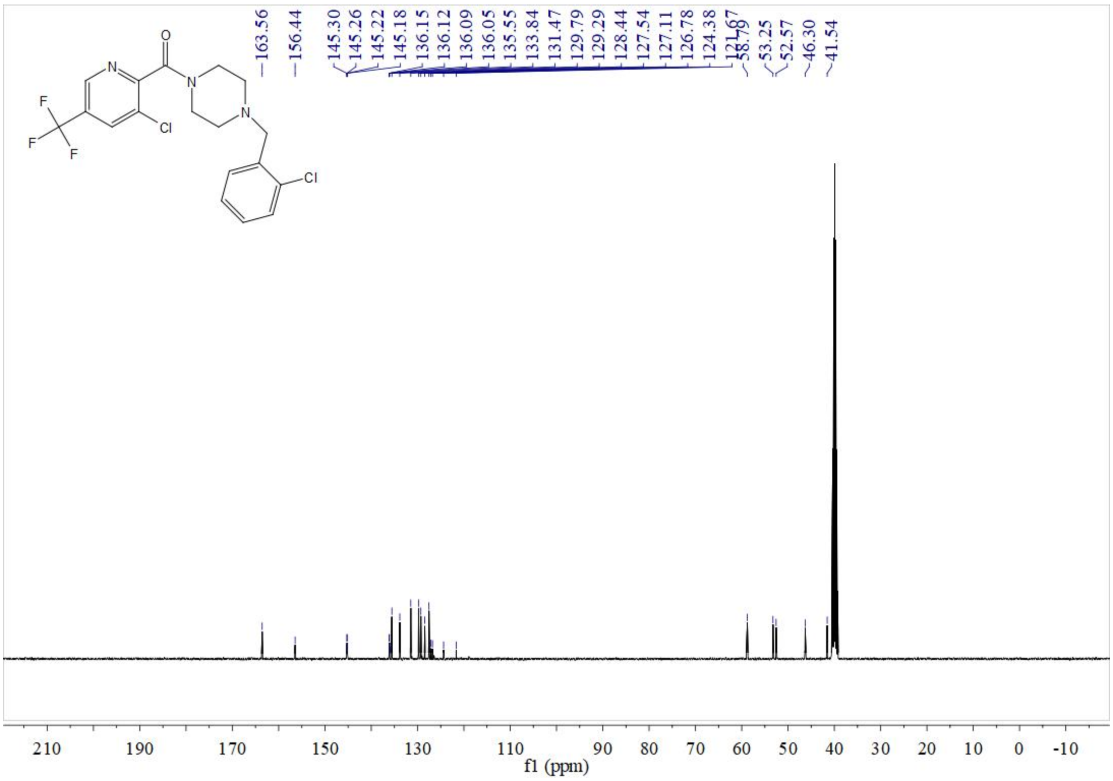

A13 ^{19}F NMR

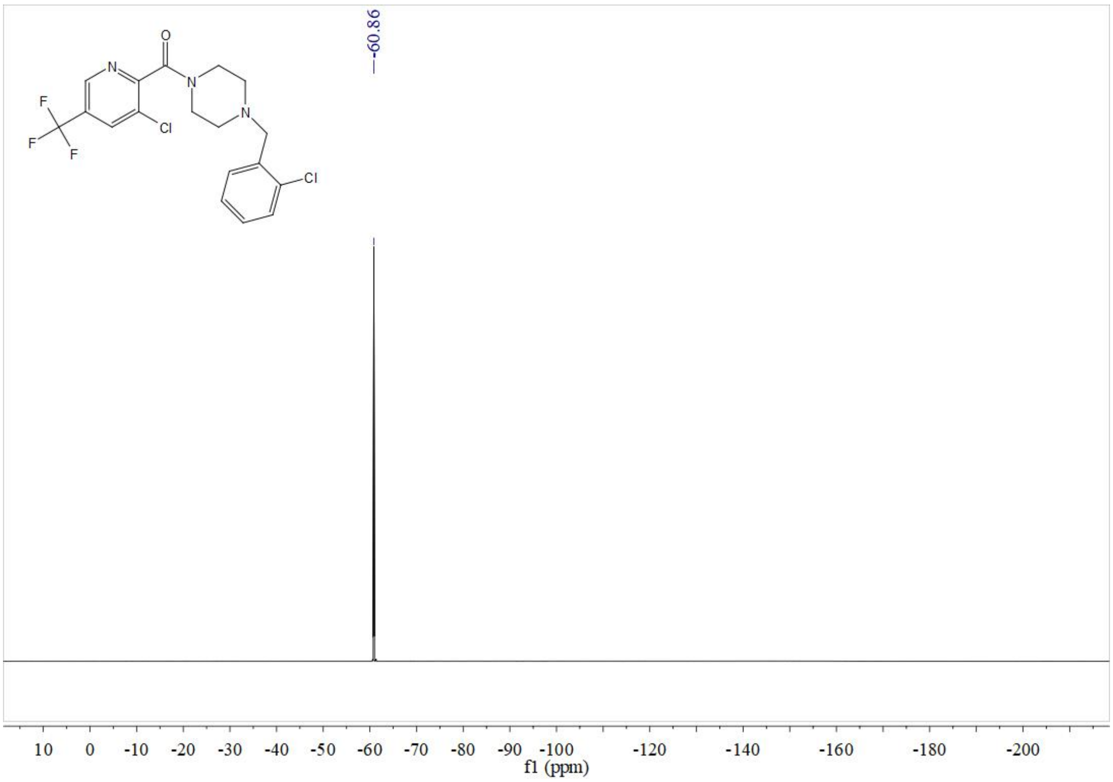

A13 HRMS

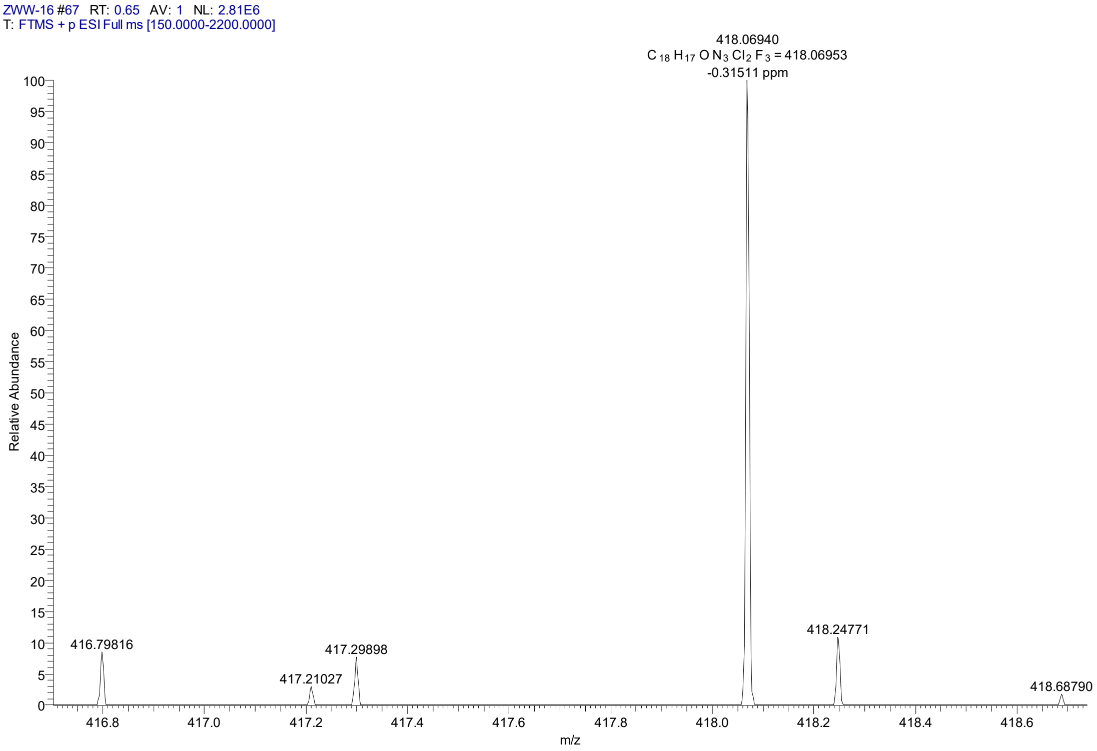

A14 ^{1}H NMR

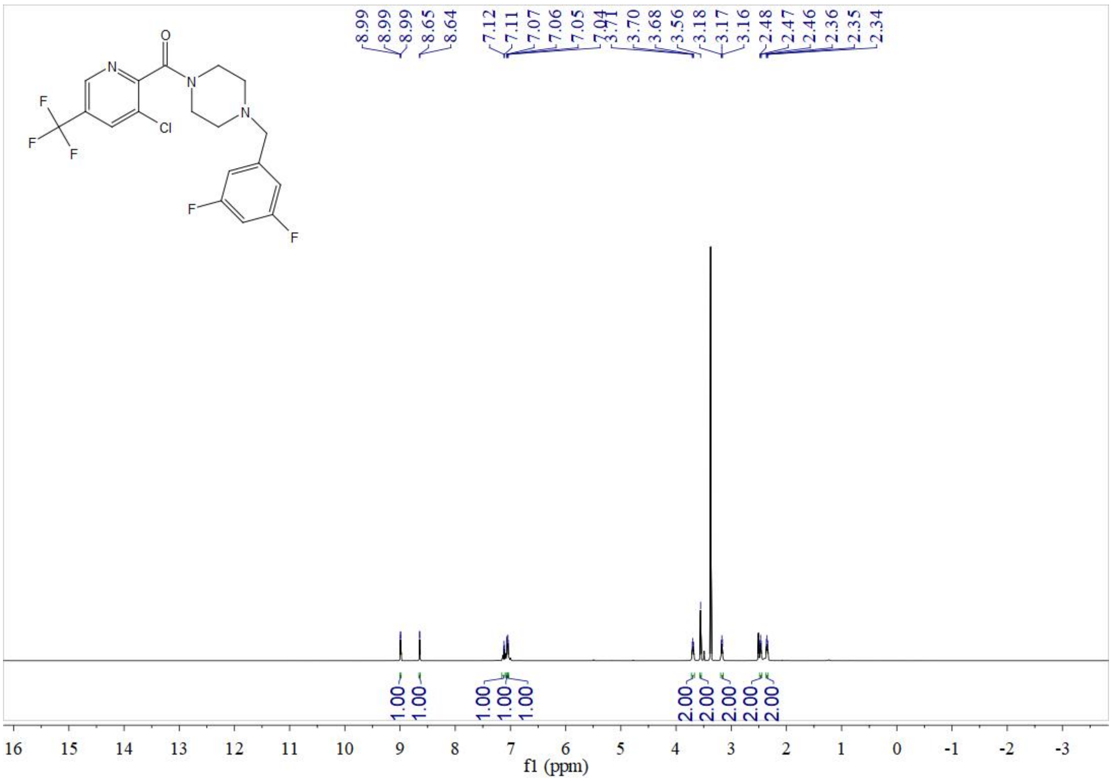

A14 ^{13}C NMR

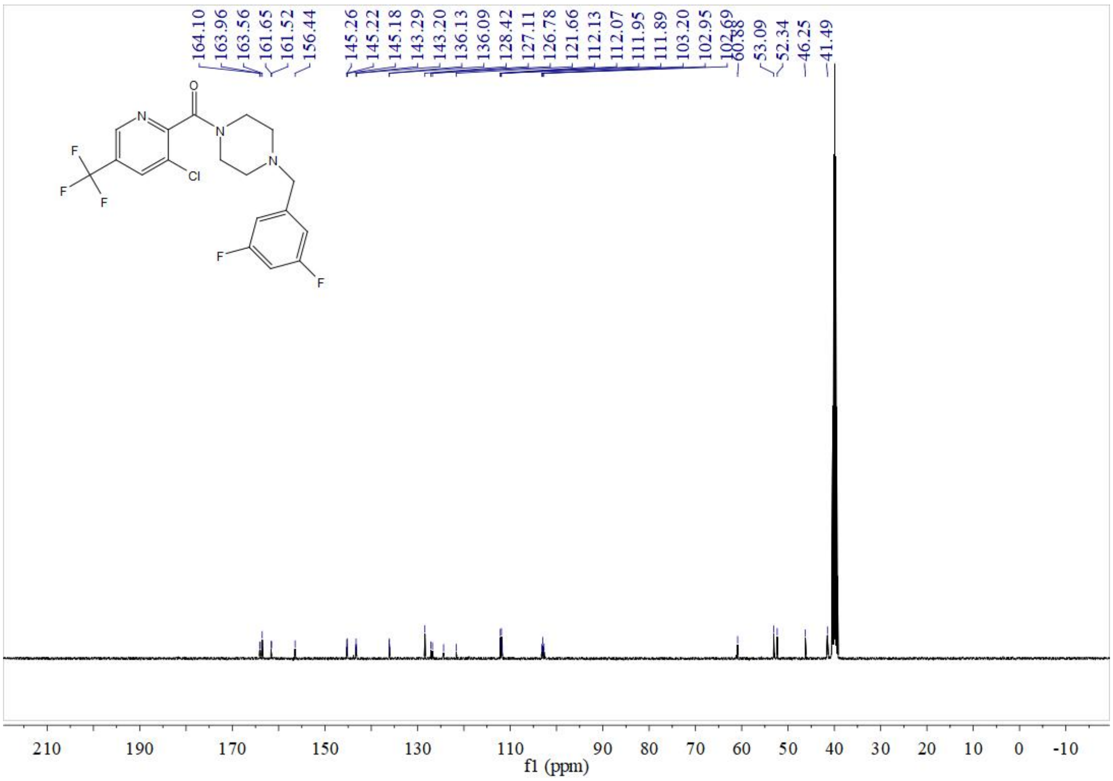

A14 ^{19}F NMR

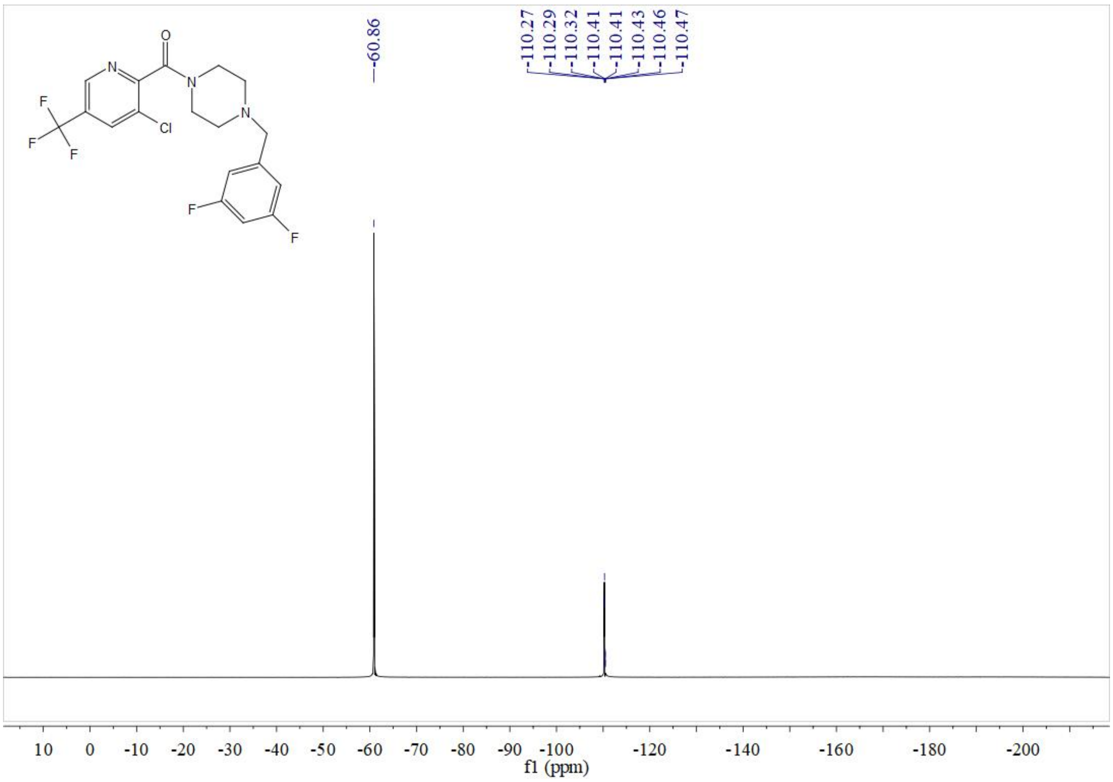

A14 HRMS

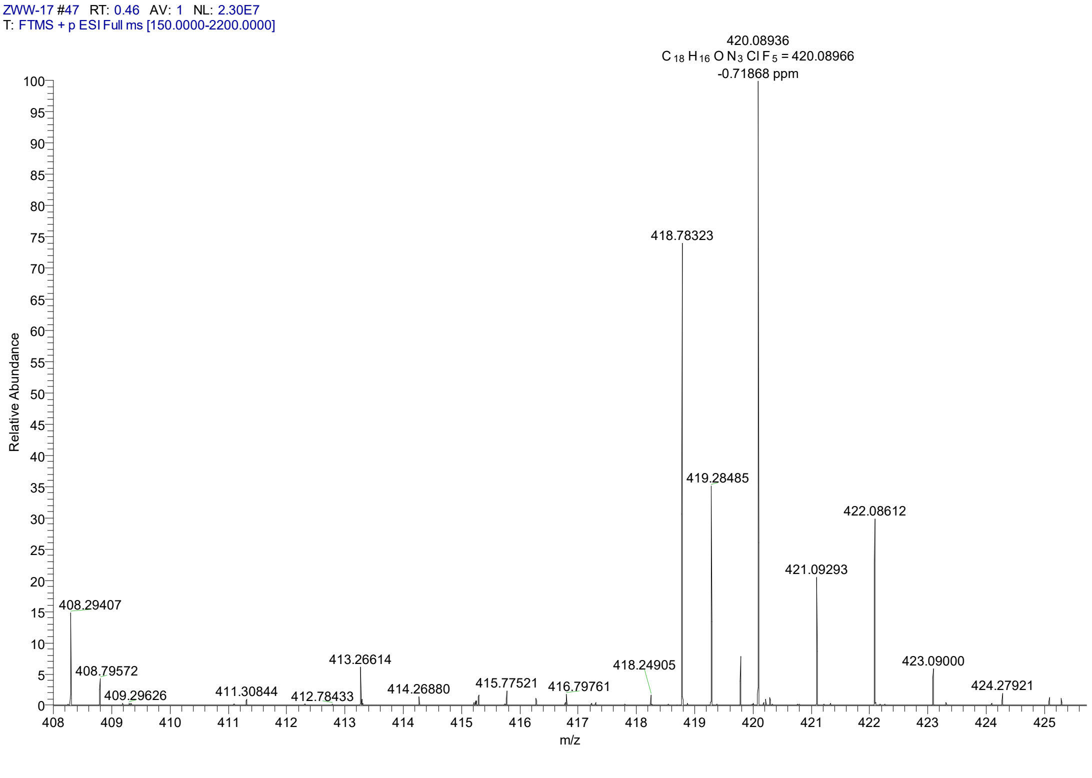

A15 ^{1}H NMR

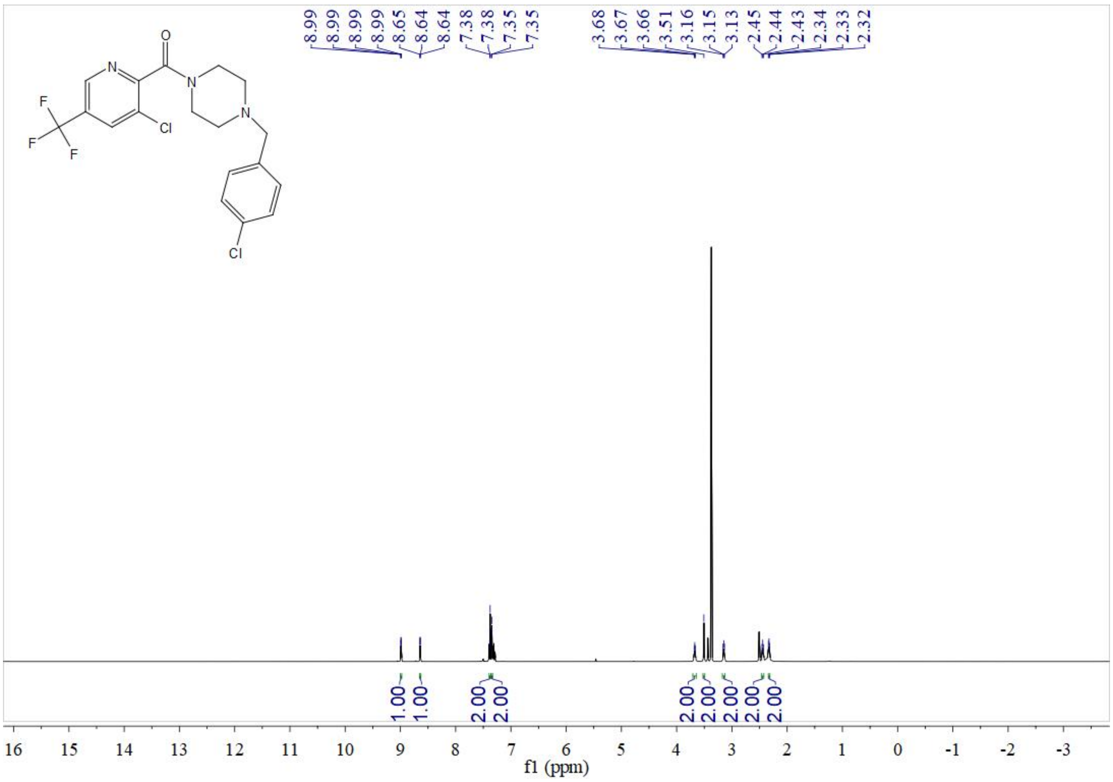

A15 ^{13}C NMR

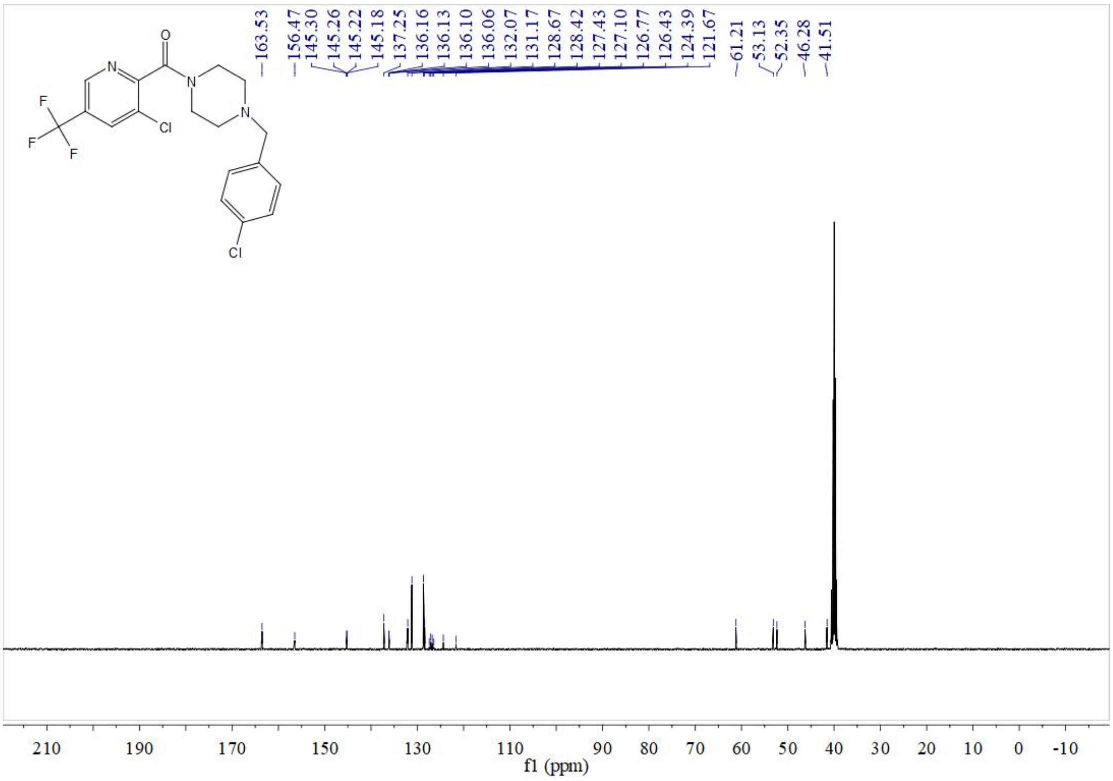

A15 ^{19}F NMR

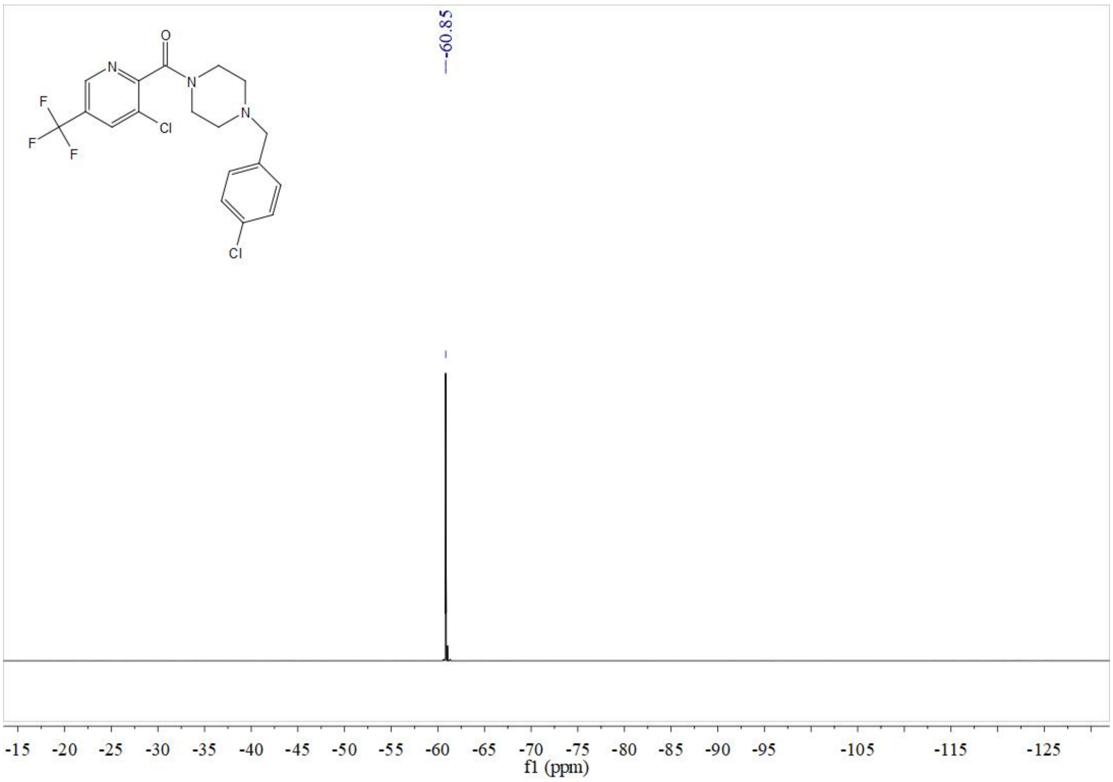

A15 HRMS

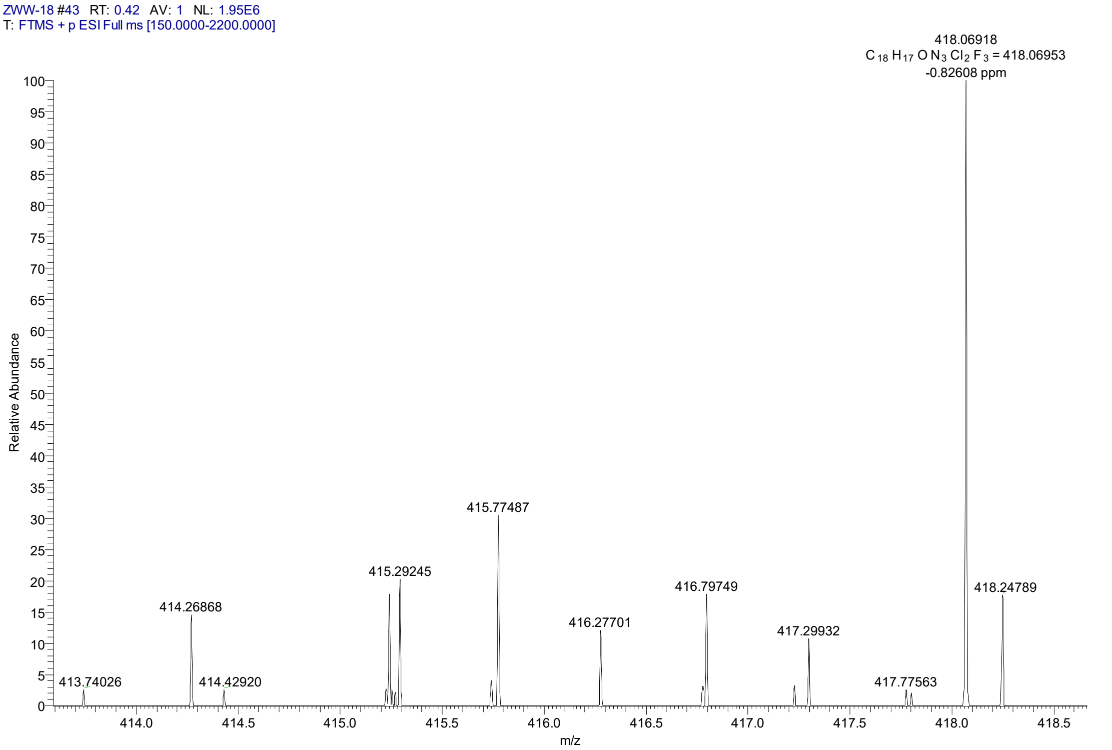

A16 ^{1}H NMR

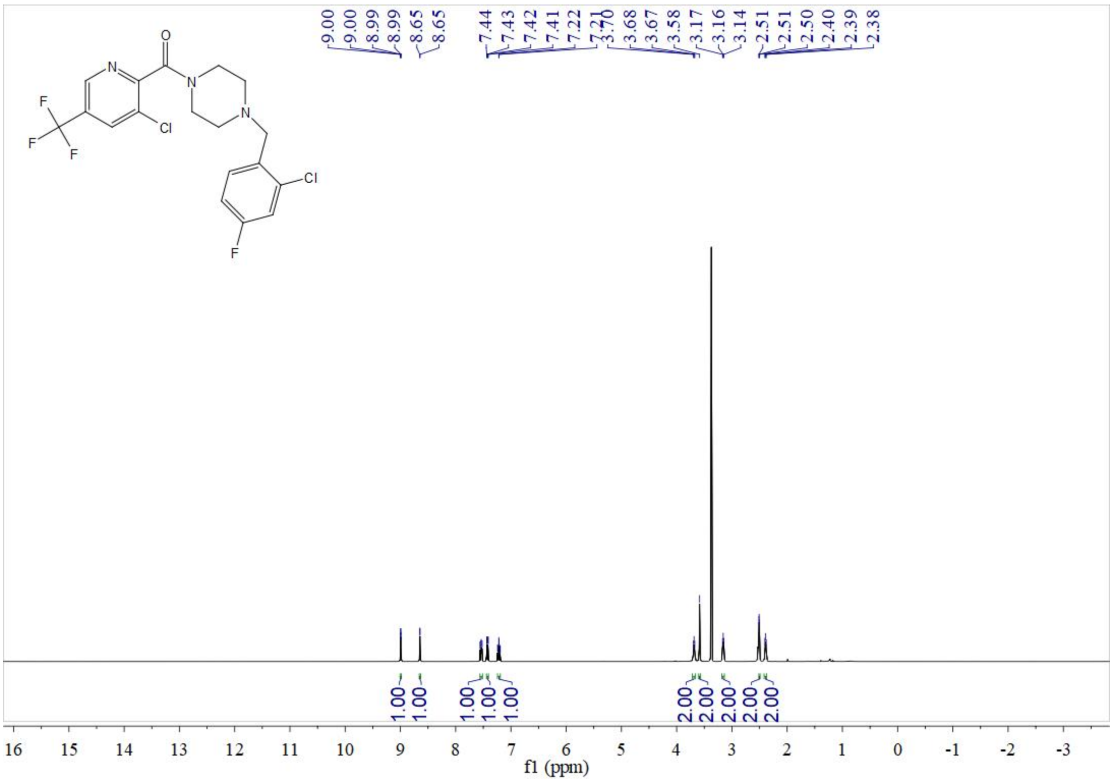

A16 ^{13}C NMR

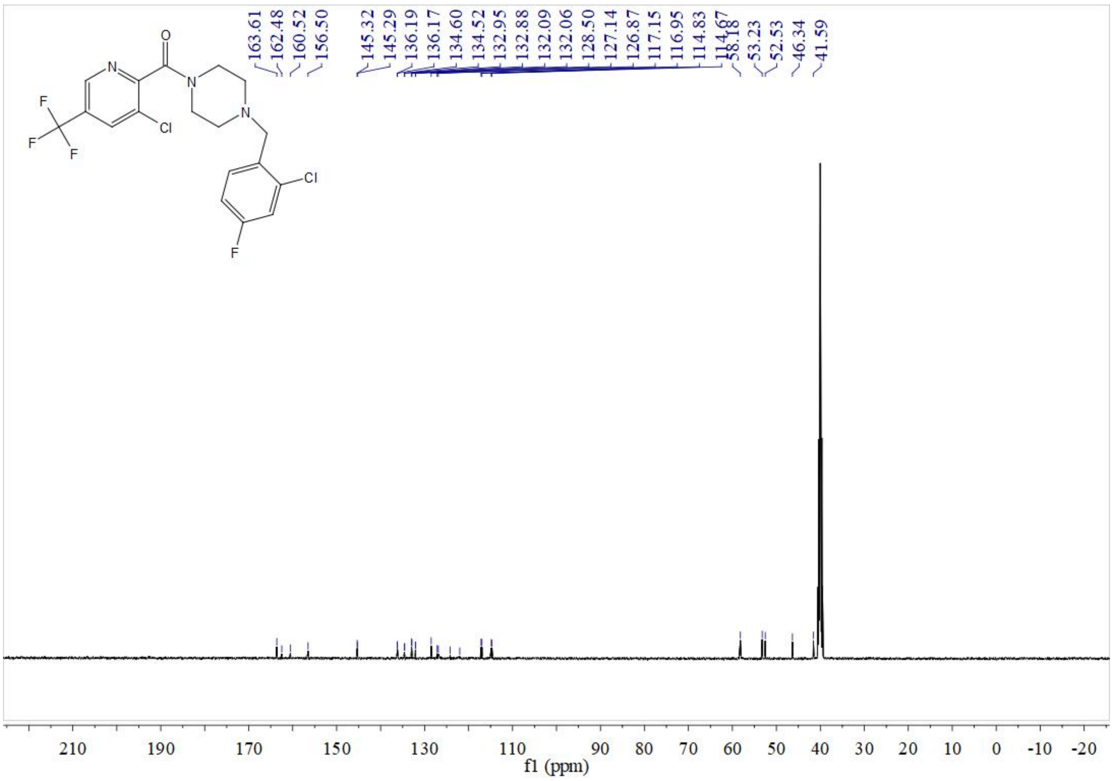

A16 ^{19}F NMR

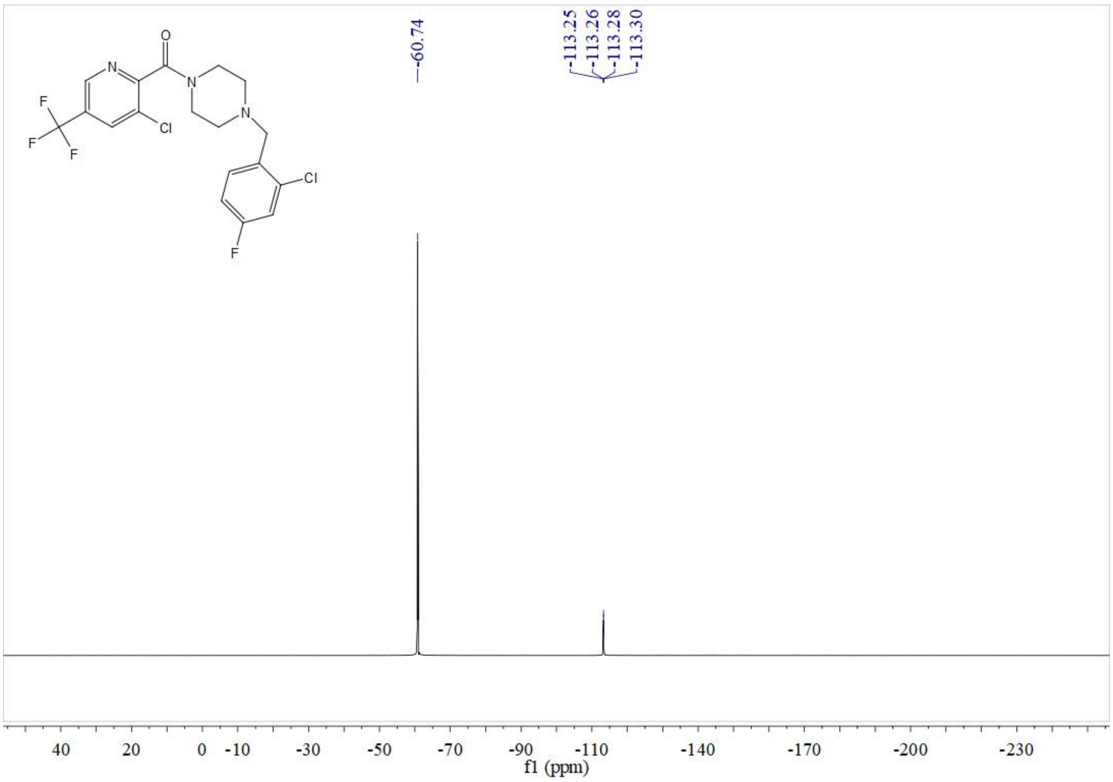

A16 HRMS

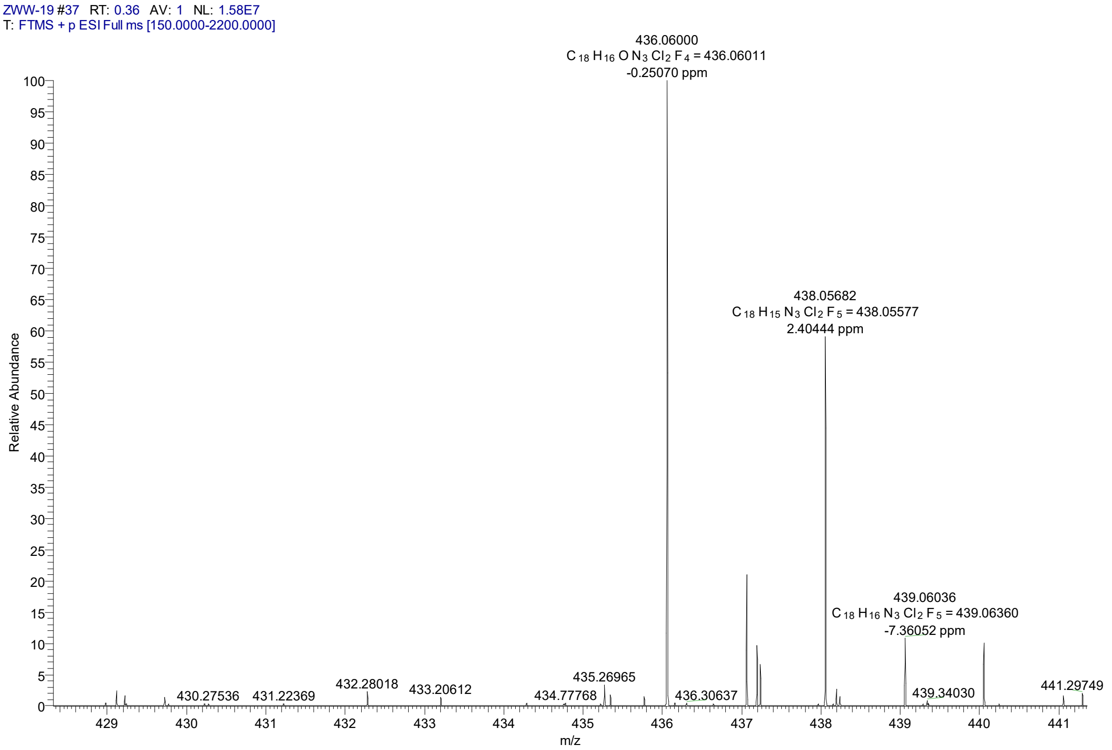

A17 ^{1}H NMR

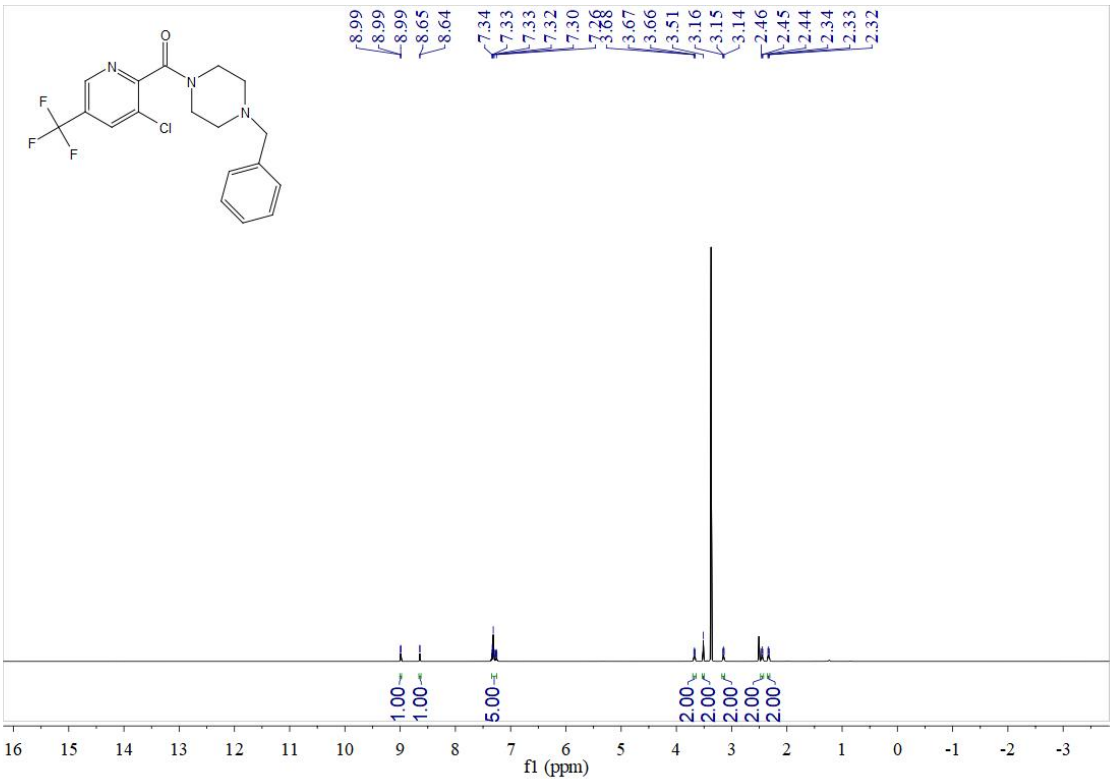

A17 ^{13}C NMR

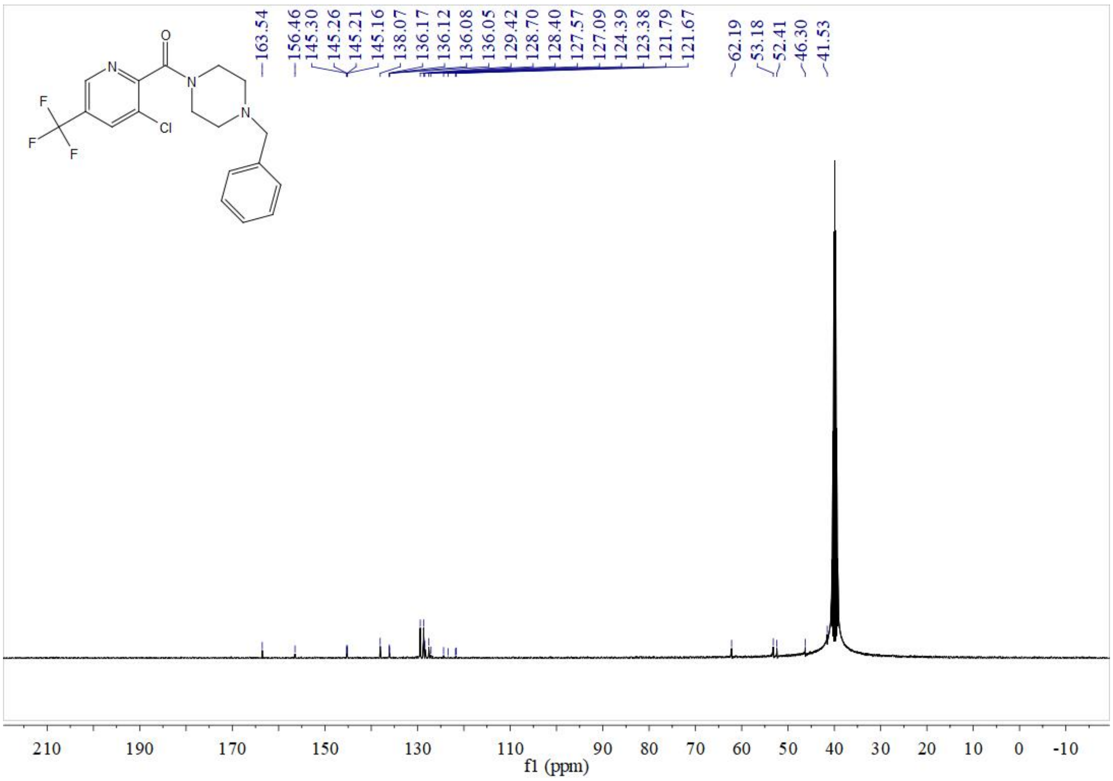

A17 ^{19}F NMR

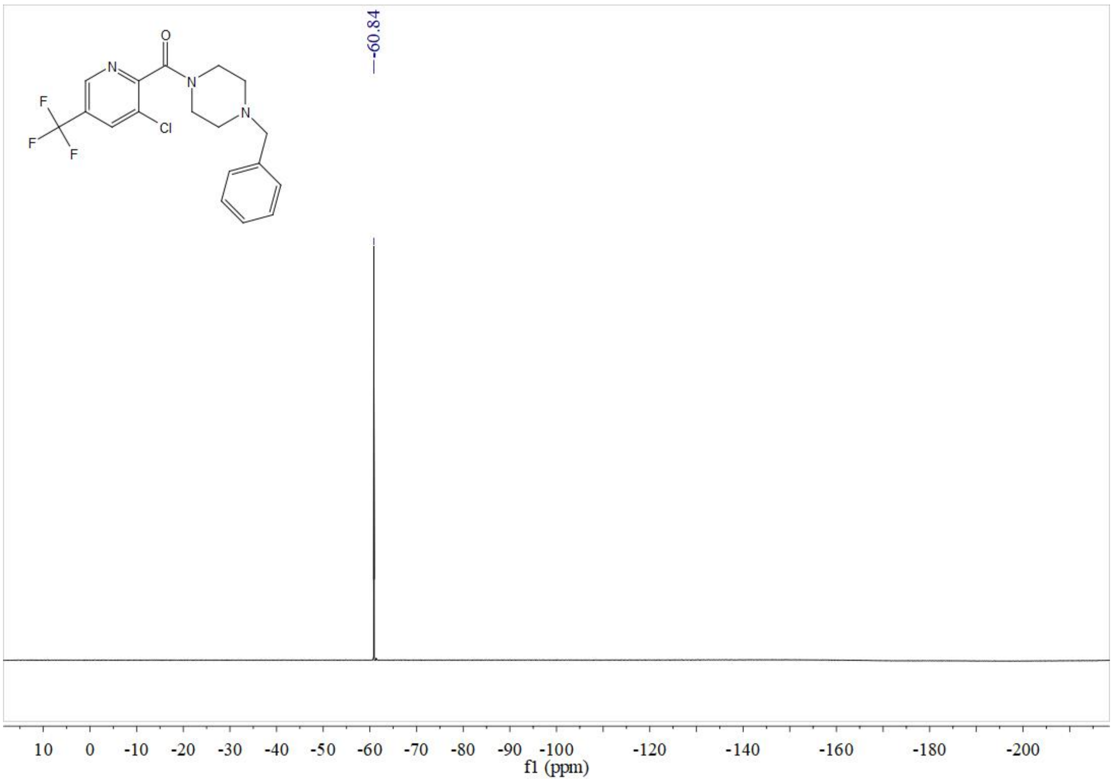

A17 HRMS

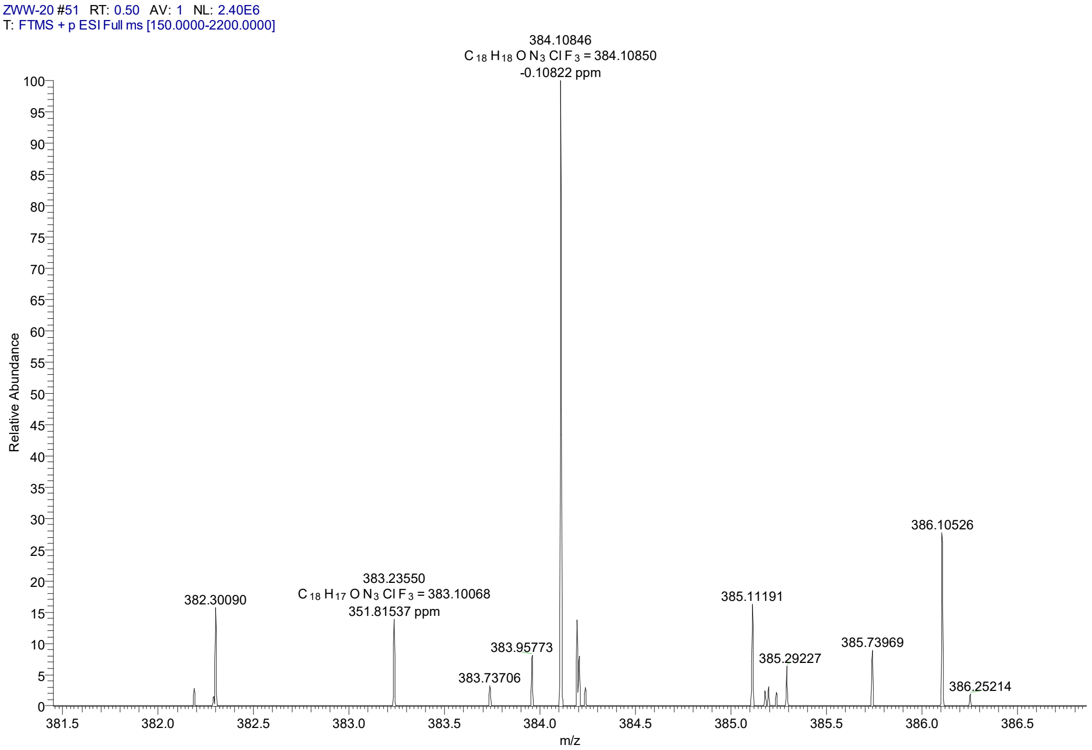

A18 ^{1}H NMR

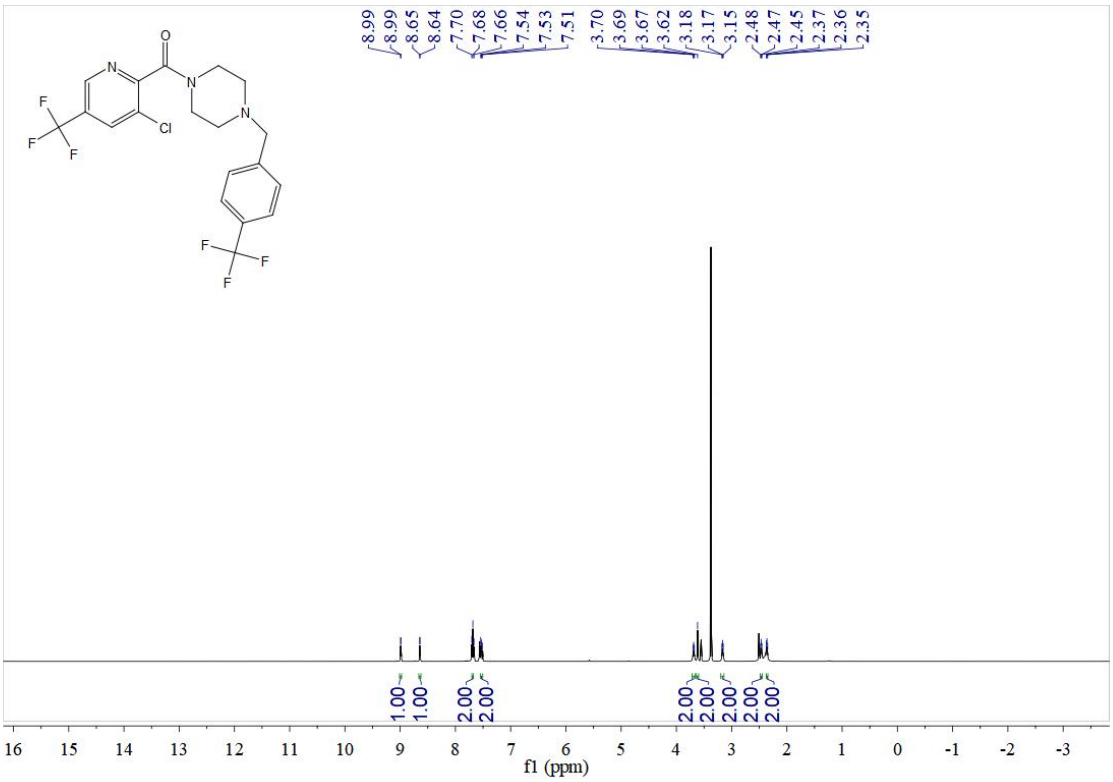

A18 ^{13}C NMR

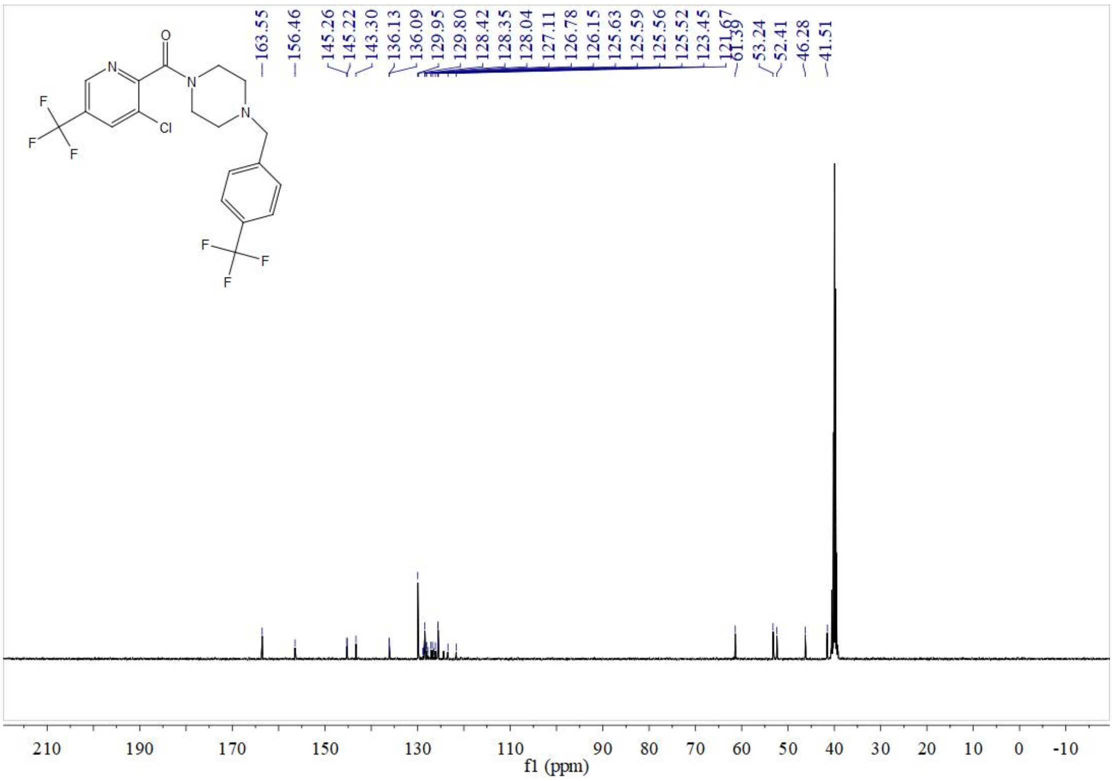

A18 ^{19}F NMR

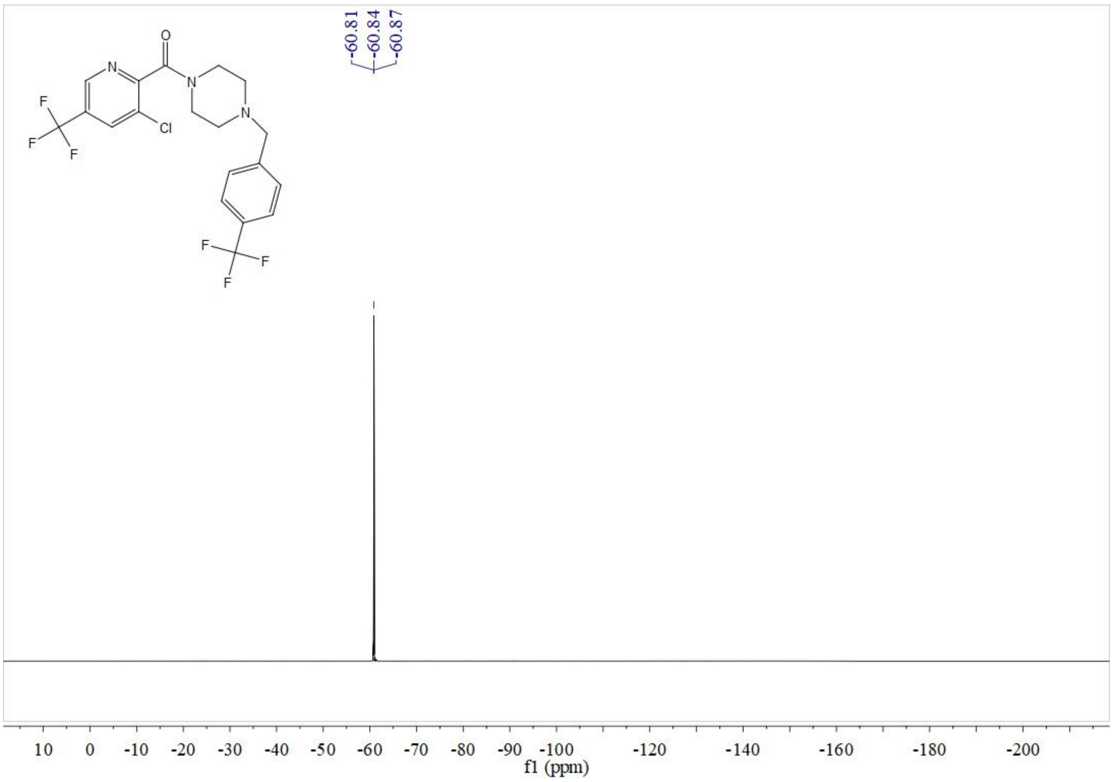

A18 HRMS

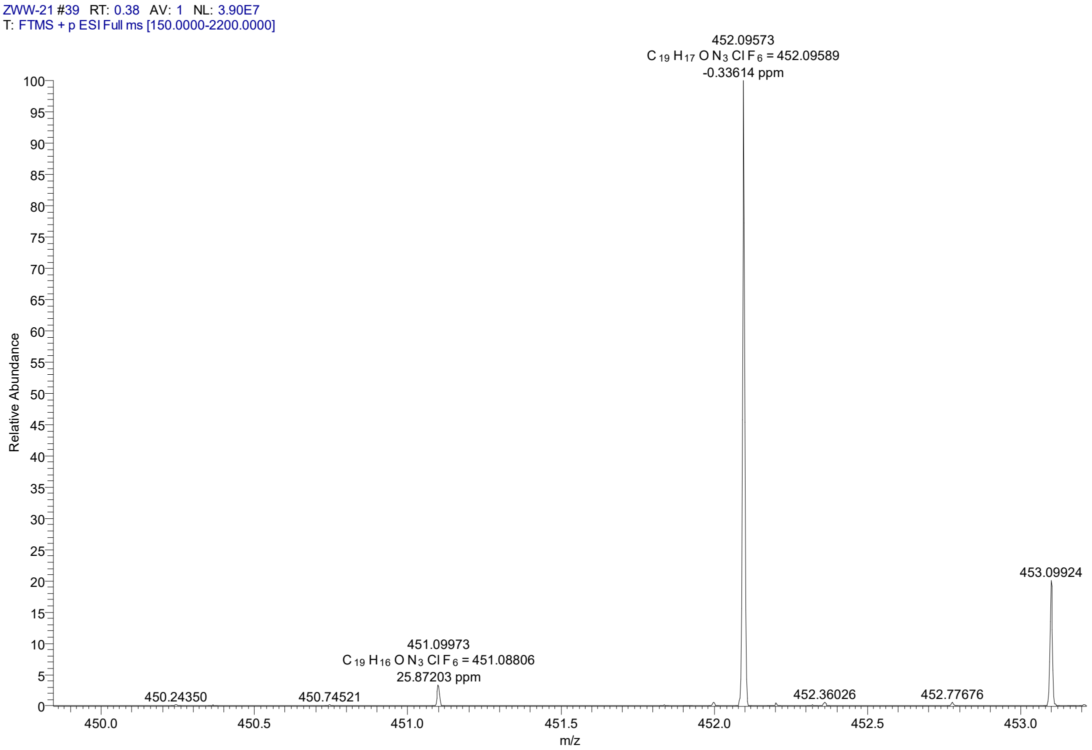

A19 ^{1}H NMR

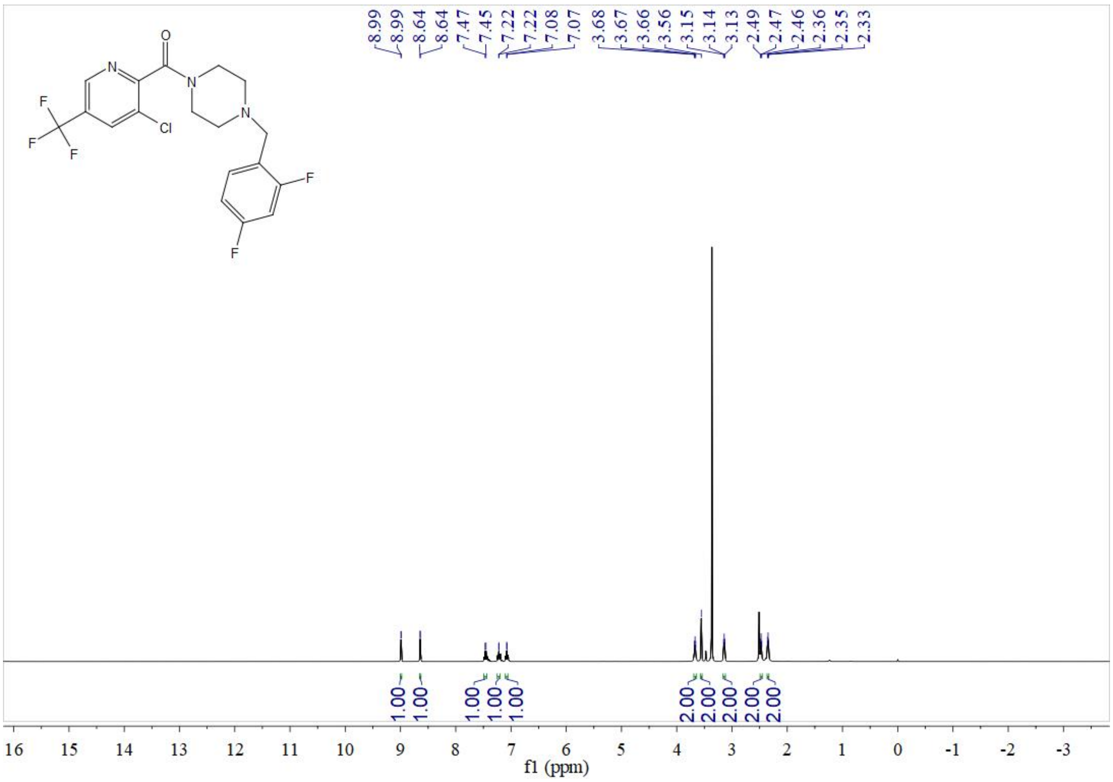

A19^{13}C NMR

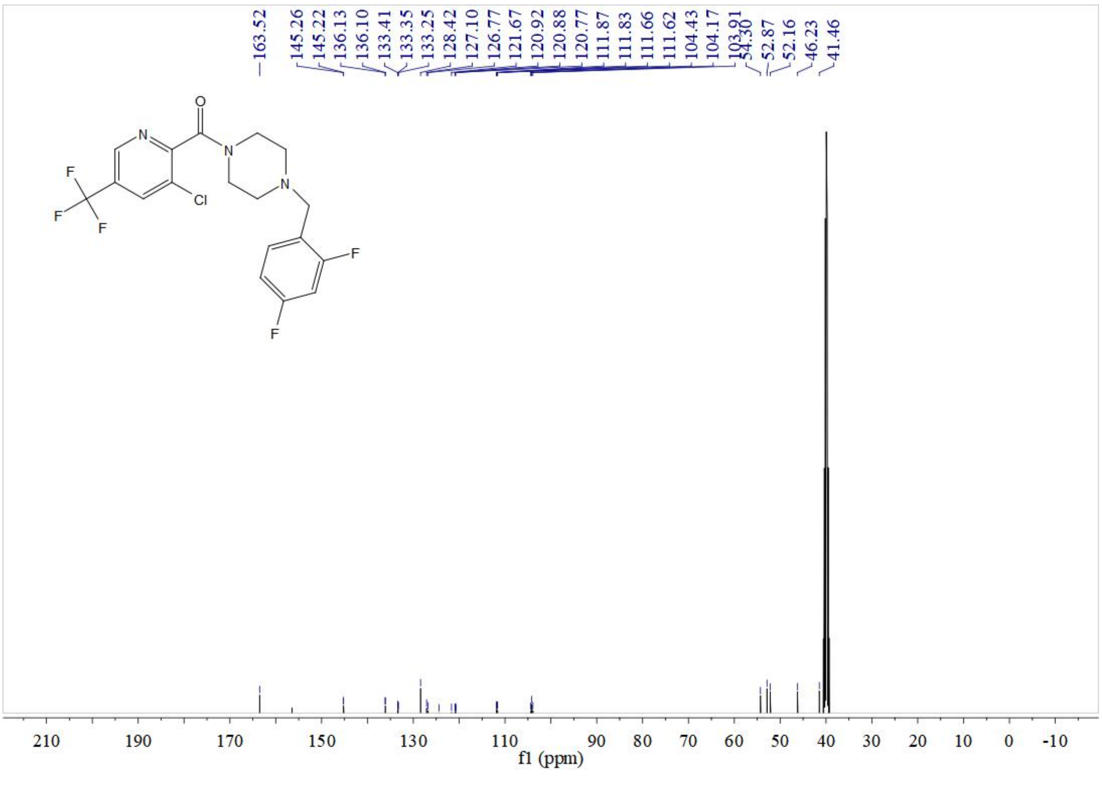

A19 ^{19}F NMR

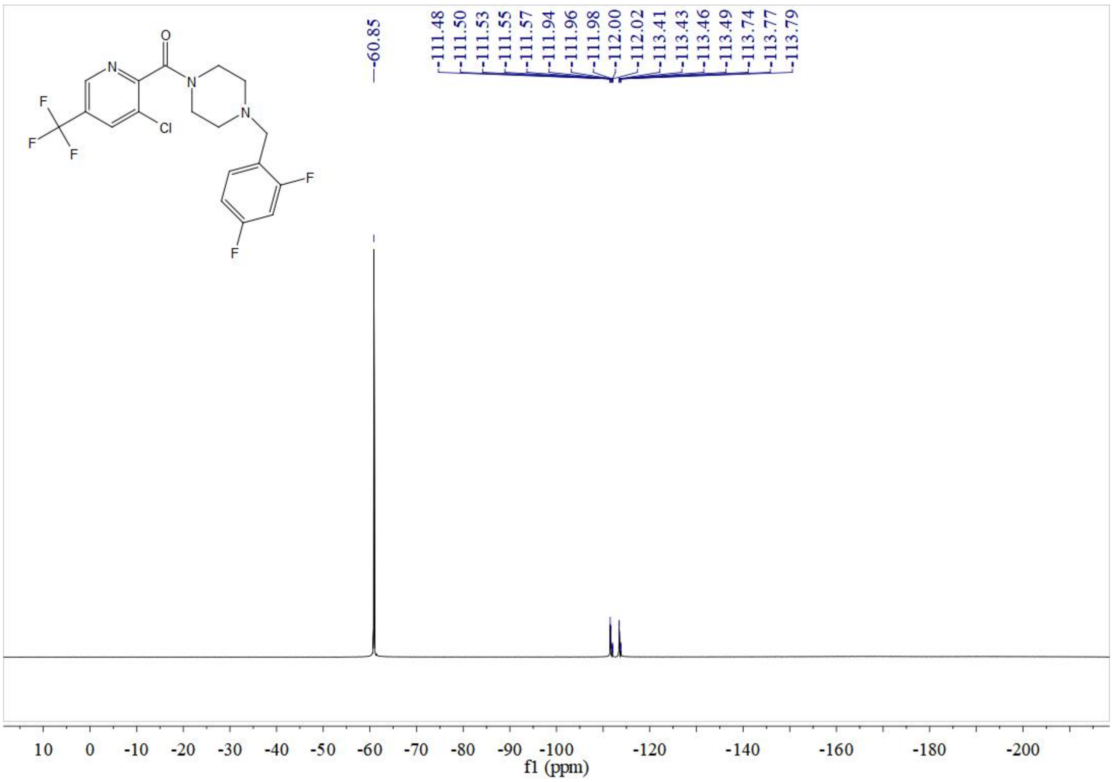

A19 HRMS

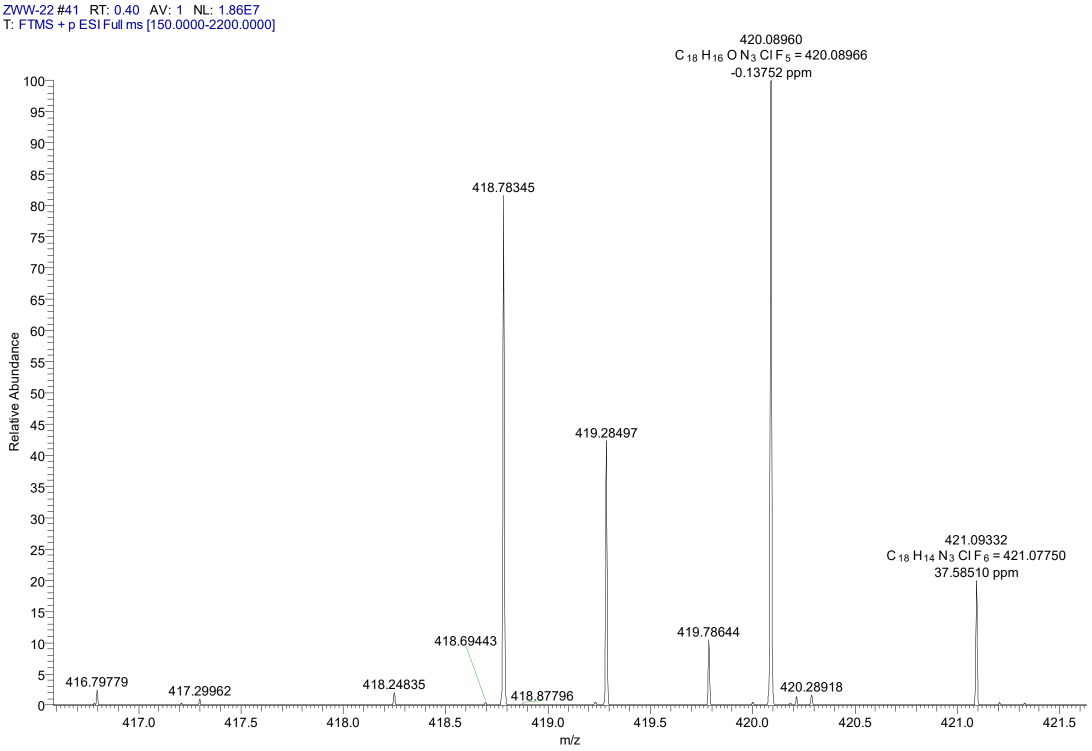

A20 ^{1}H NMR

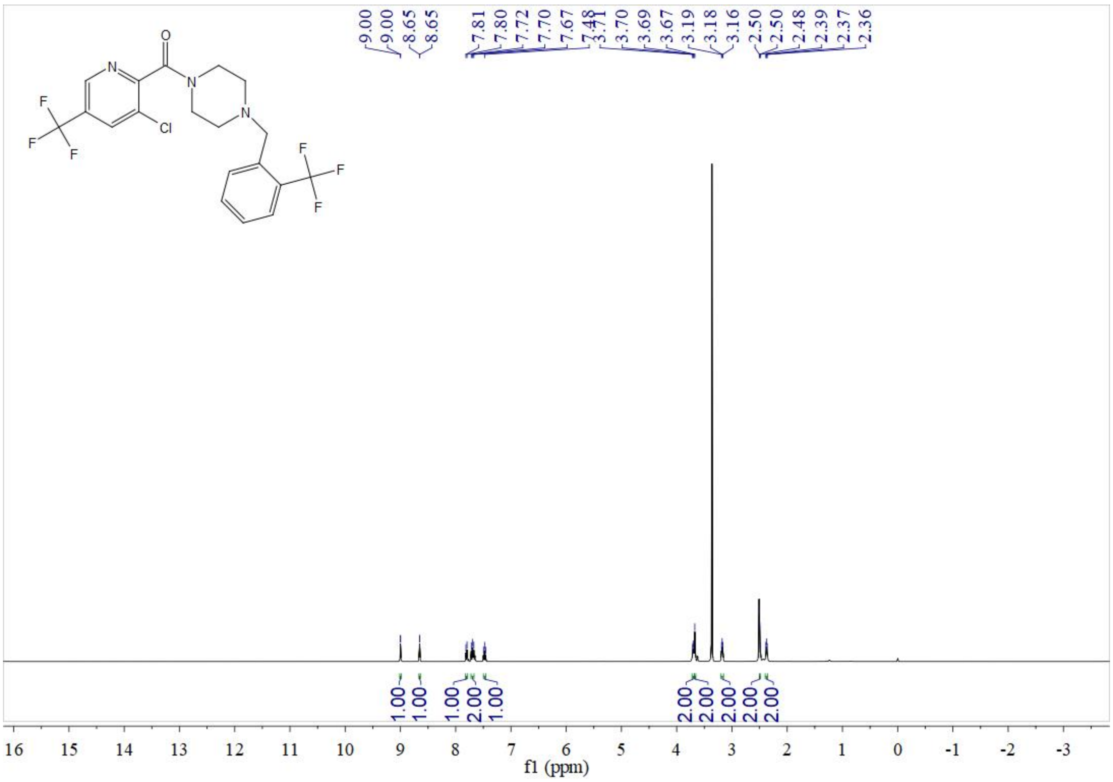

A20 ^{13}C NMR

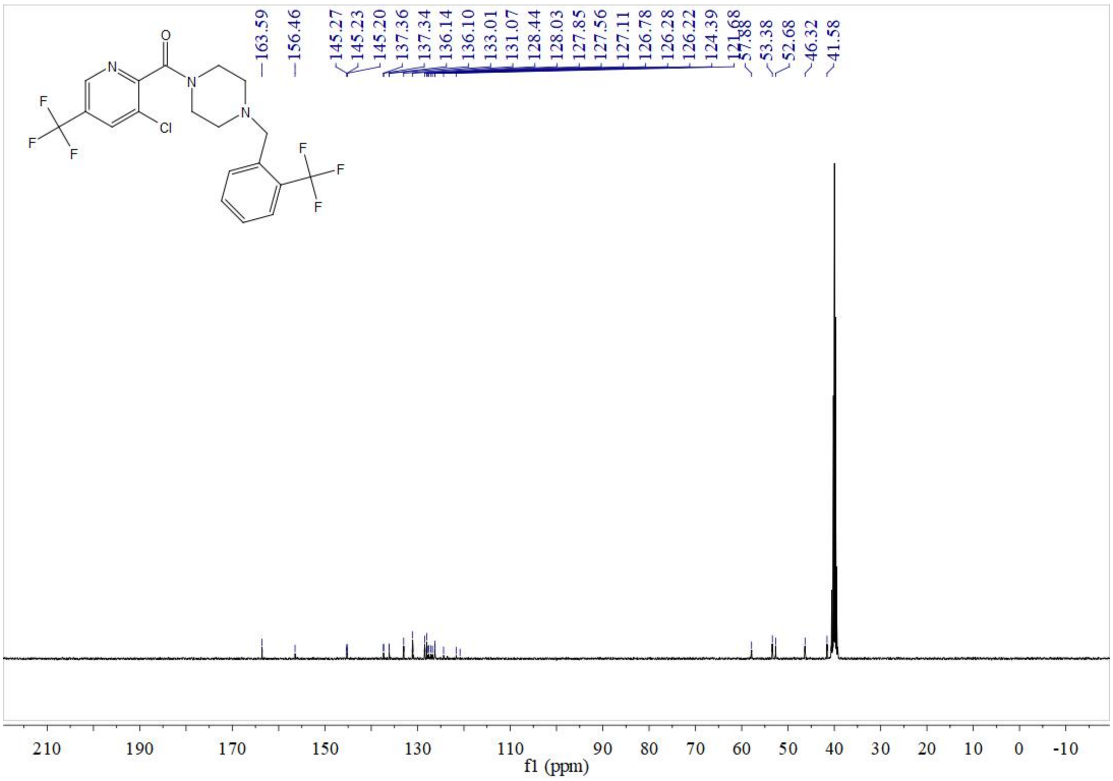

A20 ^{19}F NMR

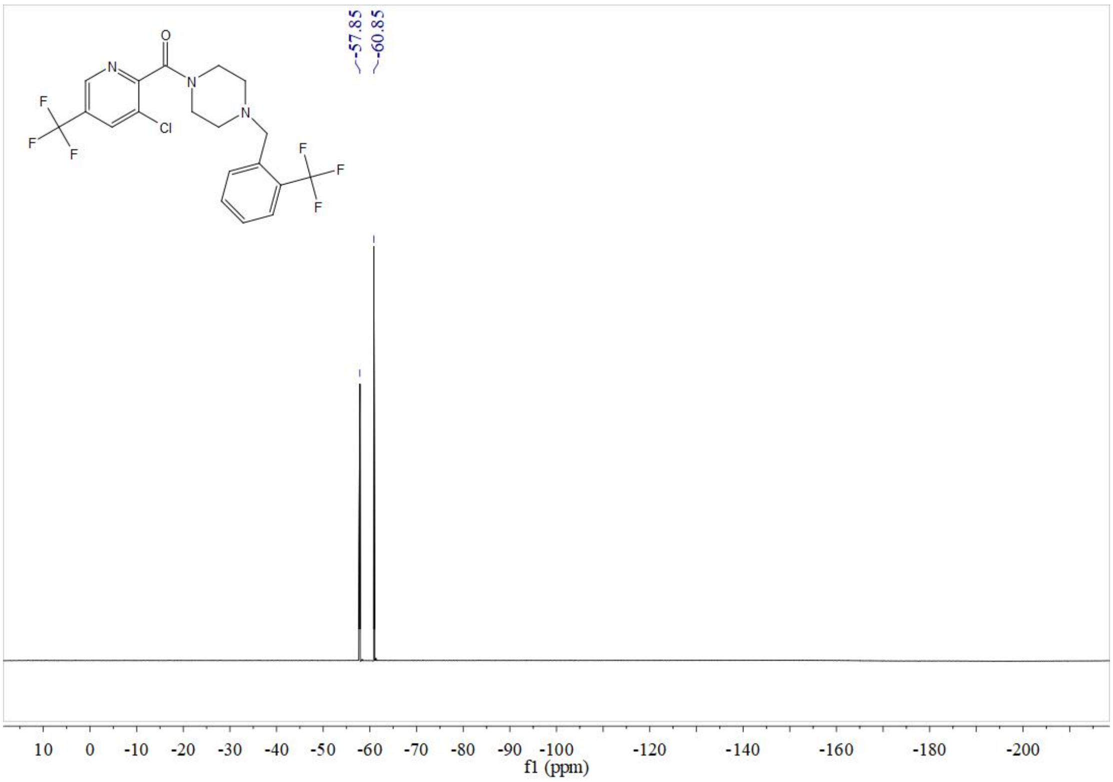

A20 HRMS

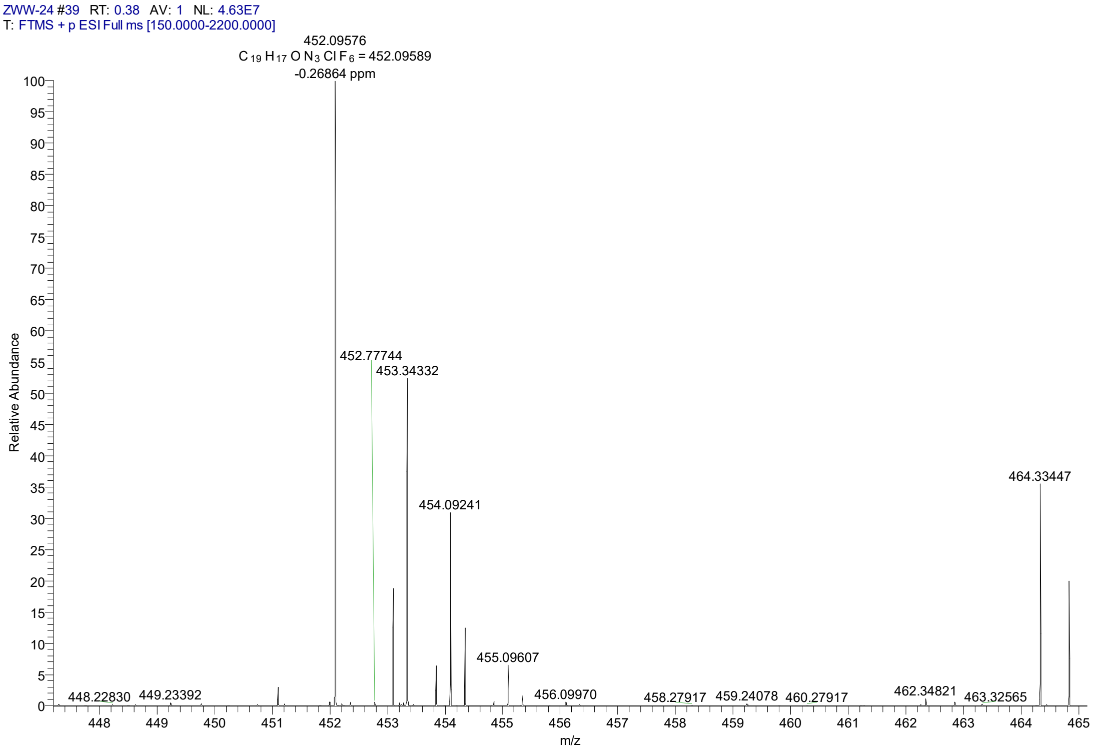

A21 ^{1}H NMR

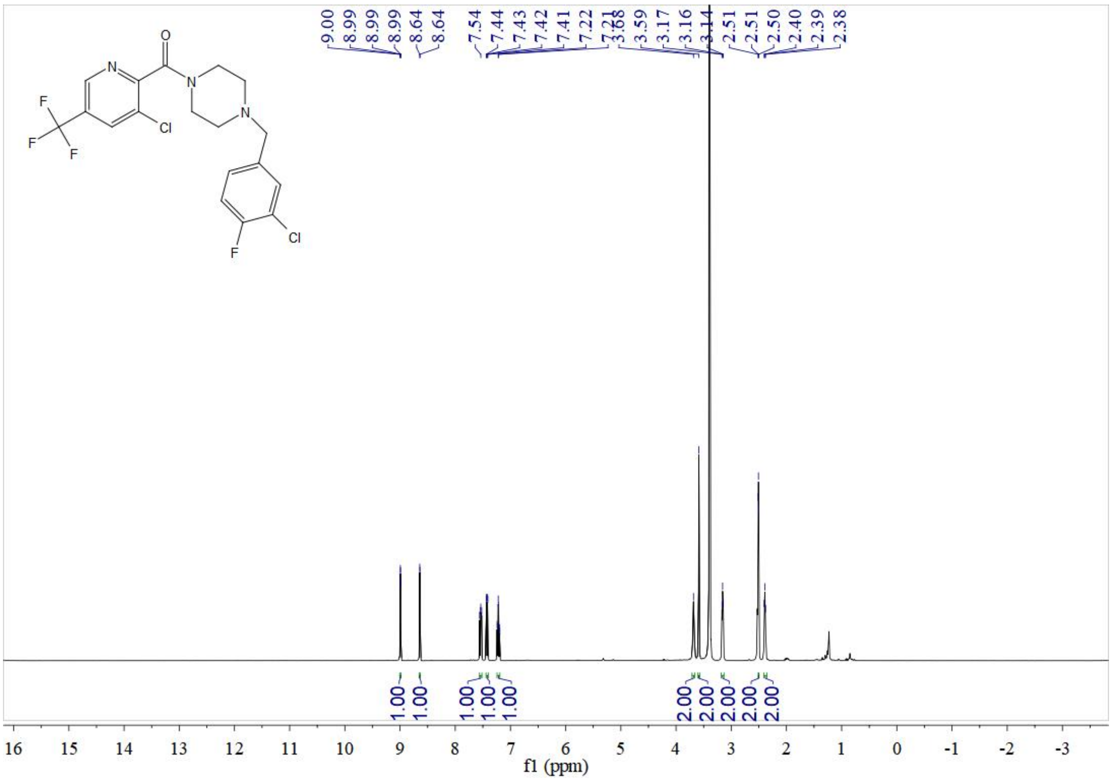

A21 ^{13}C NMR

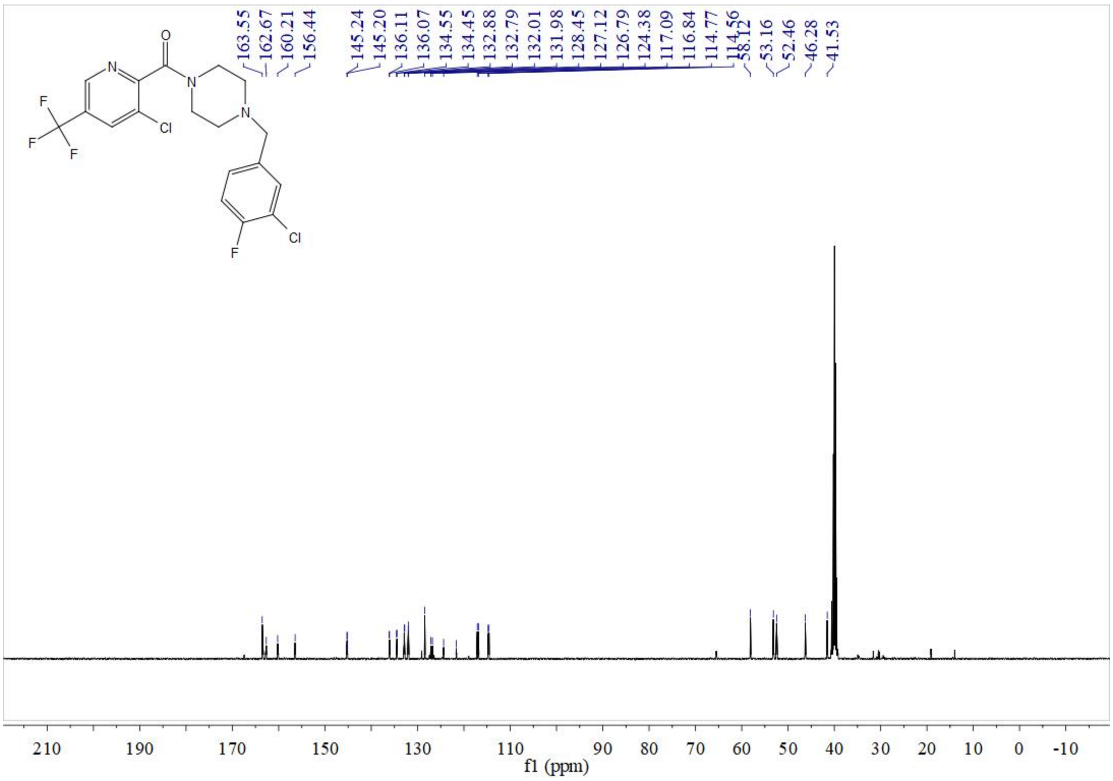

A21 ^{19}F NMR

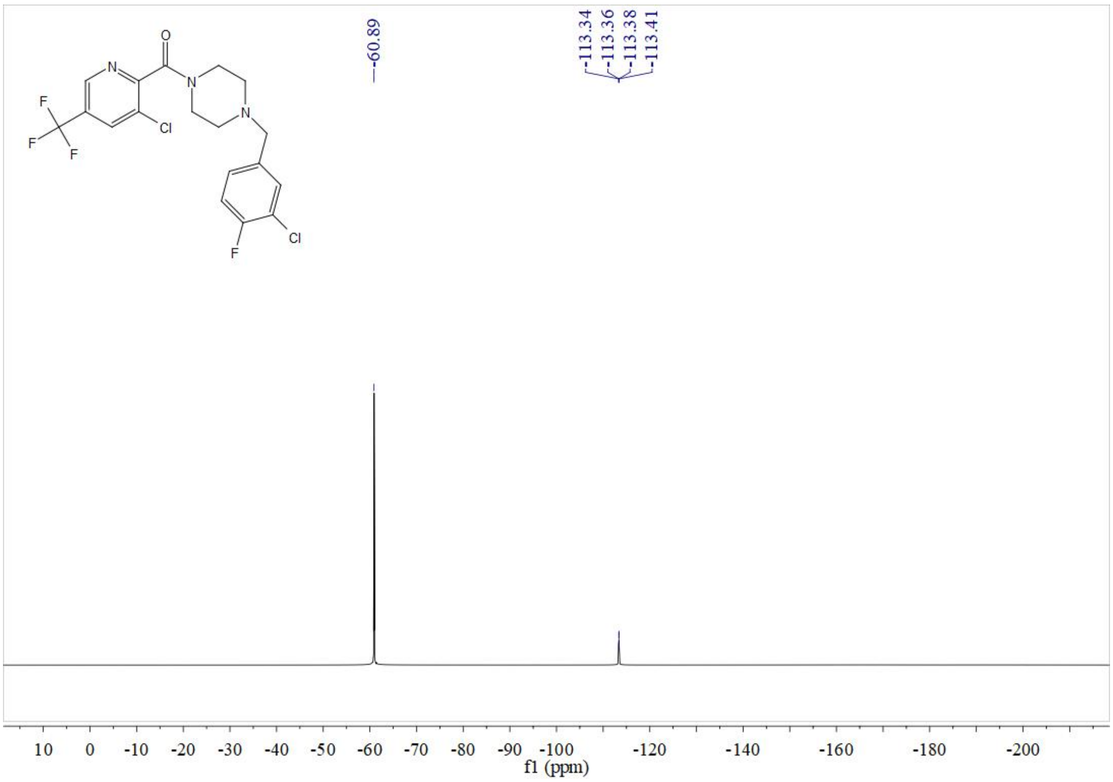

A21 HRMS

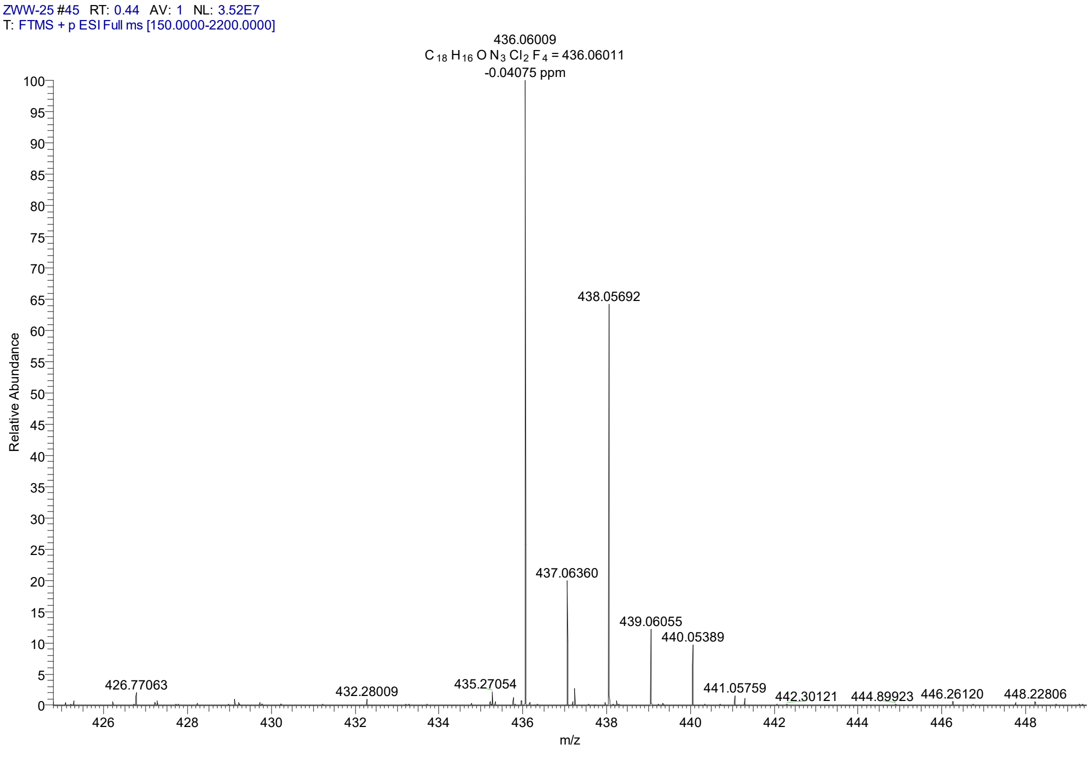

A22 ^{1}H NMR

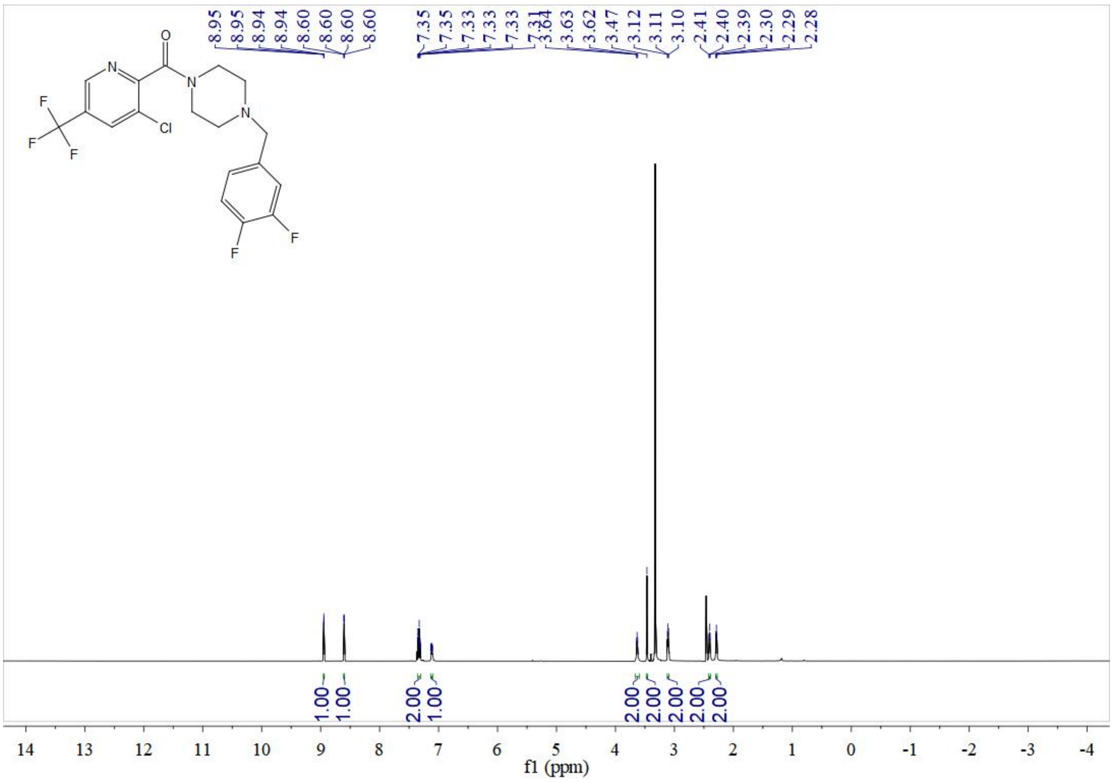

A22 ^{13}C NMR

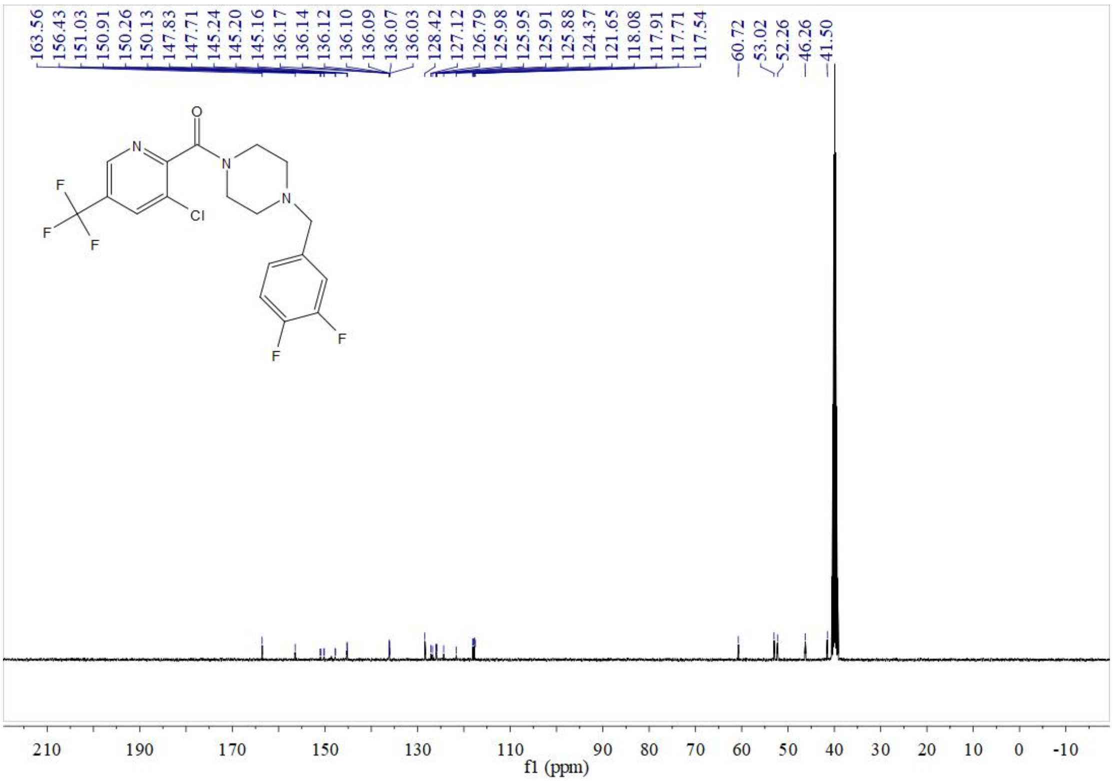

A22 ^{19}F NMR

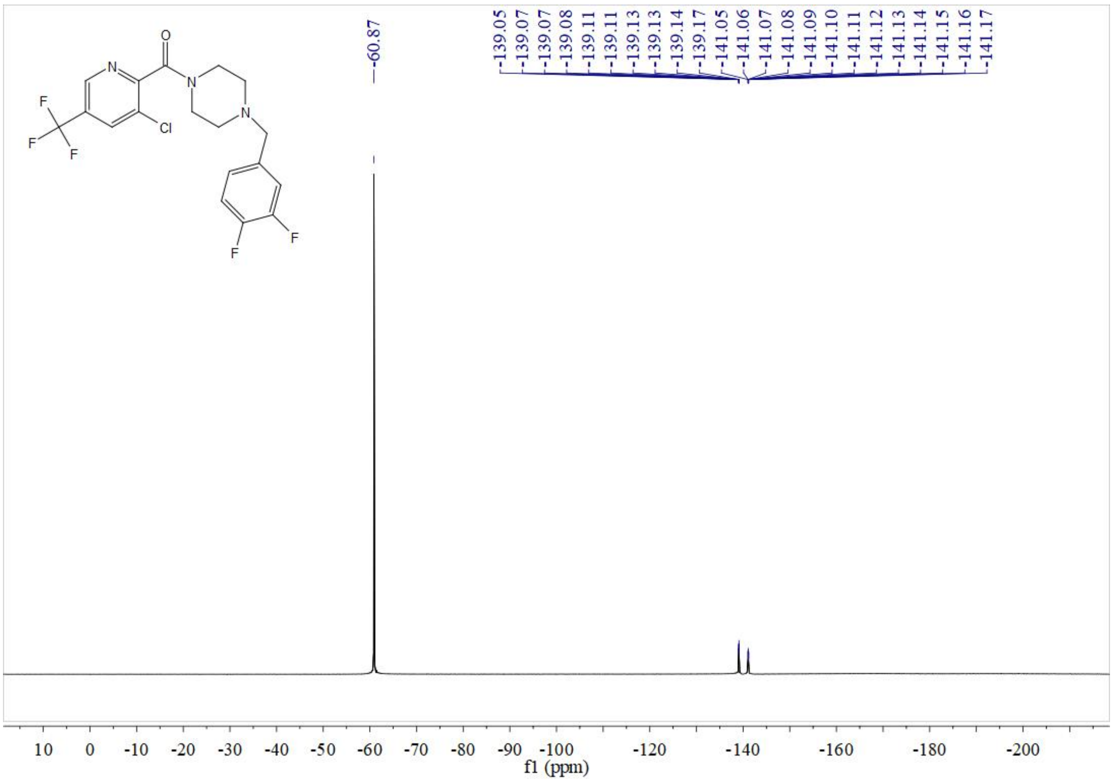

A22 HRMS

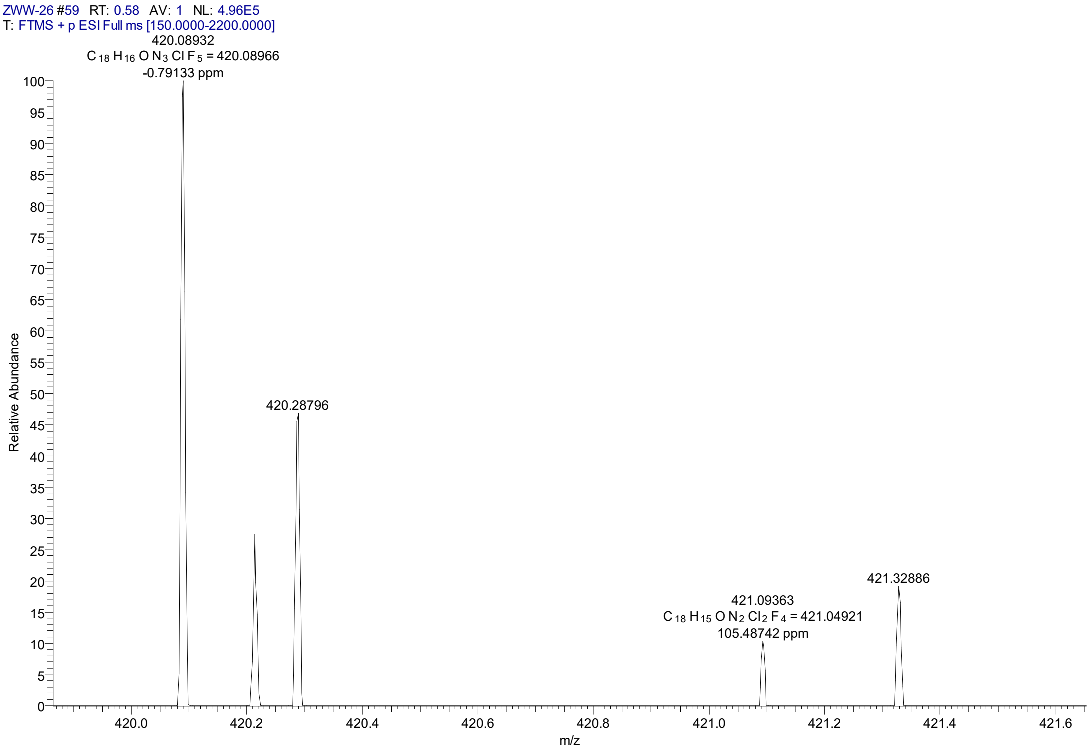

A23 ^{1}H NMR

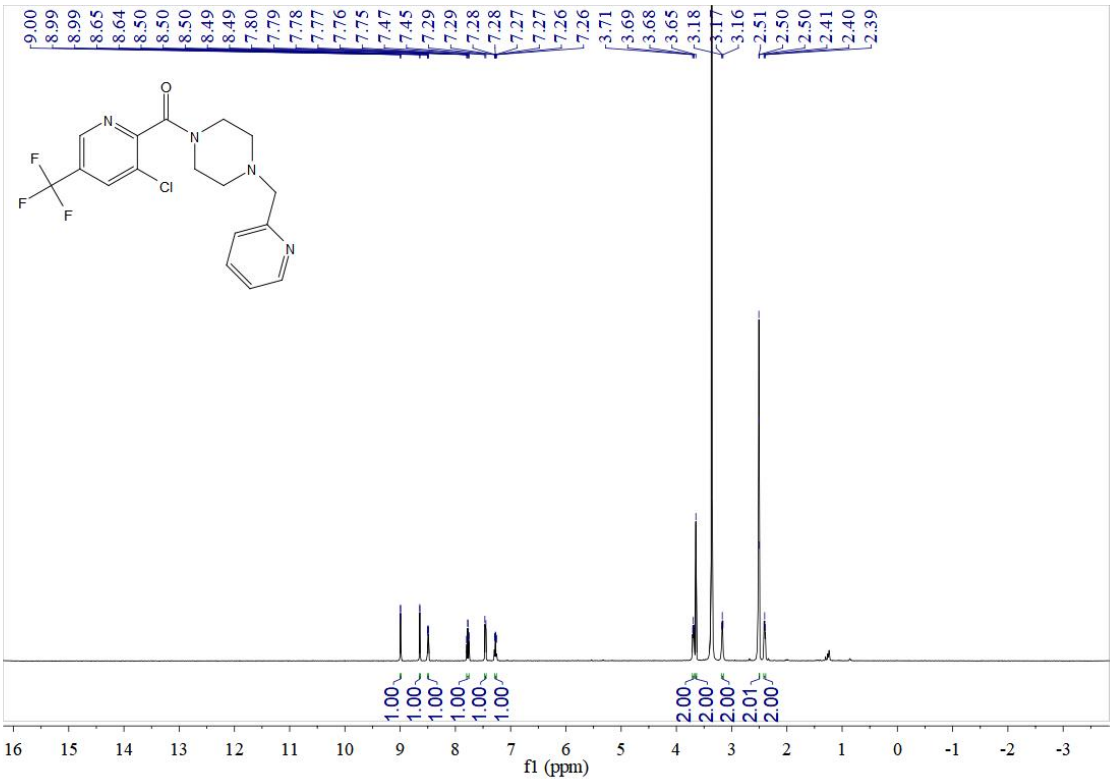

A23 ^{13}C NMR

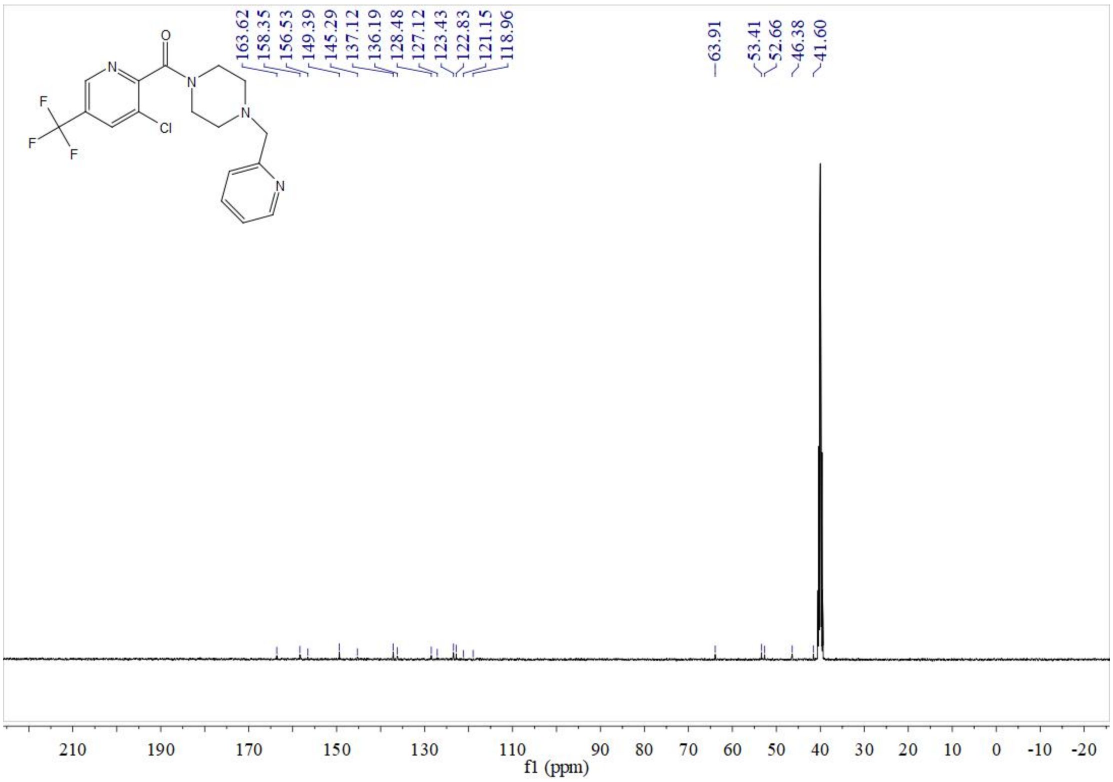

A23 ^{19}F NMR

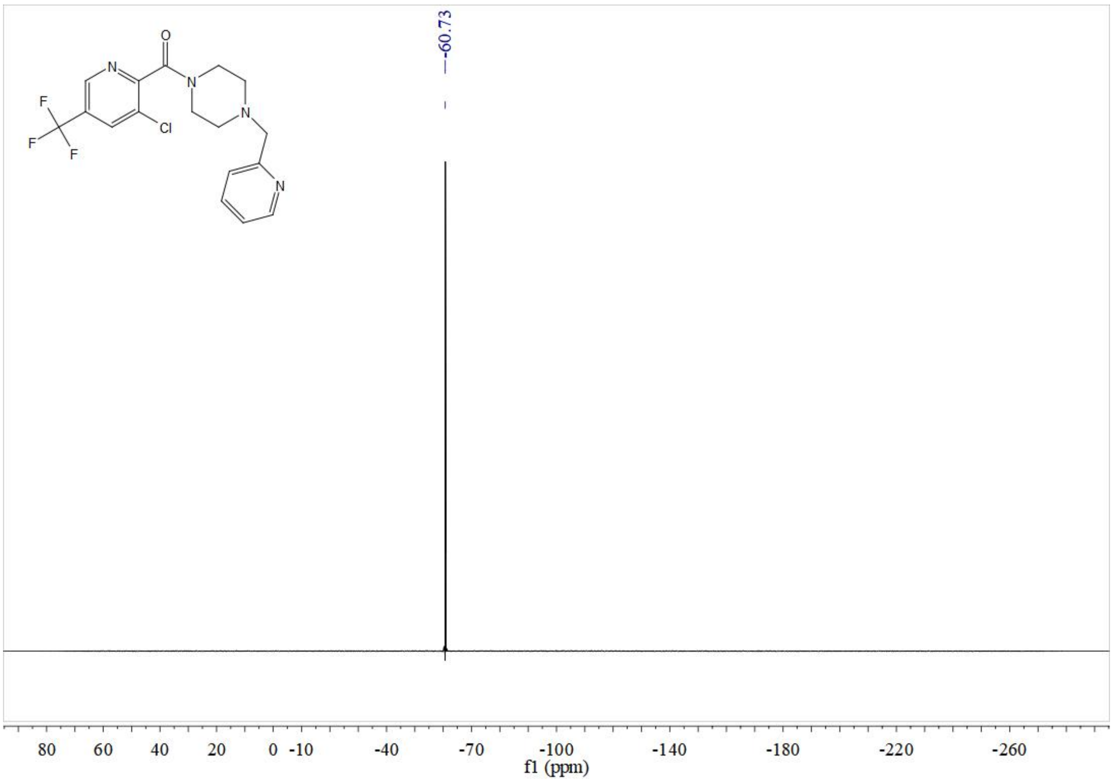

A23 HRMS

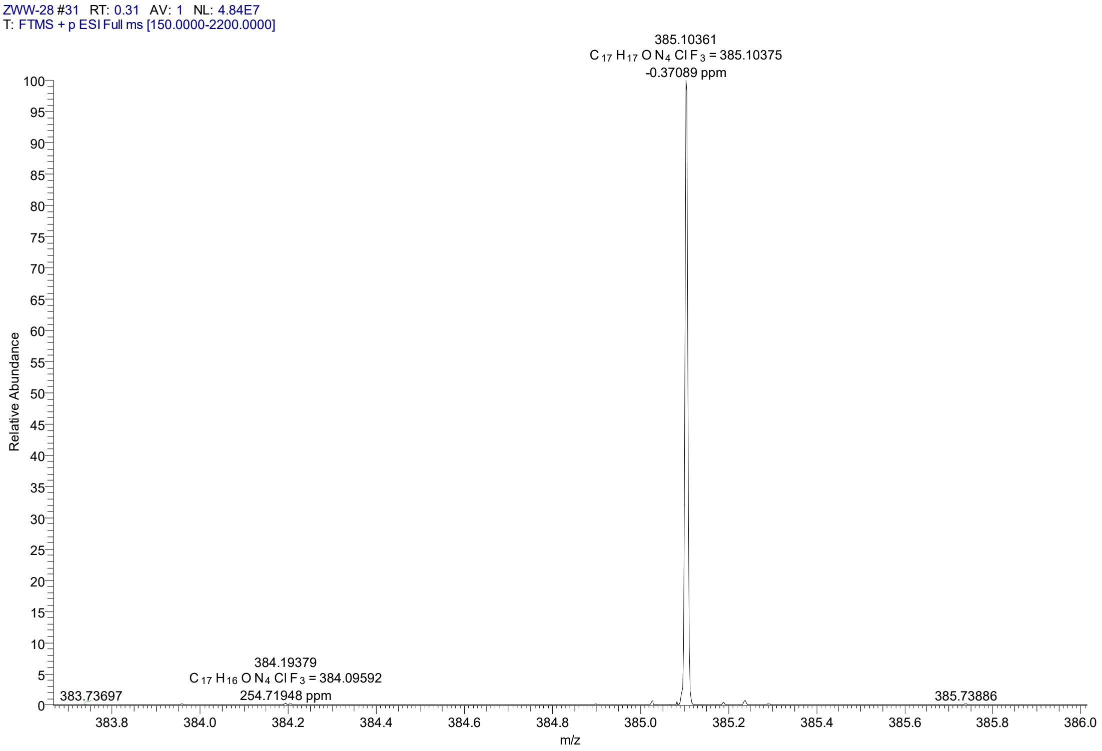

A24 ^{1}H NMR

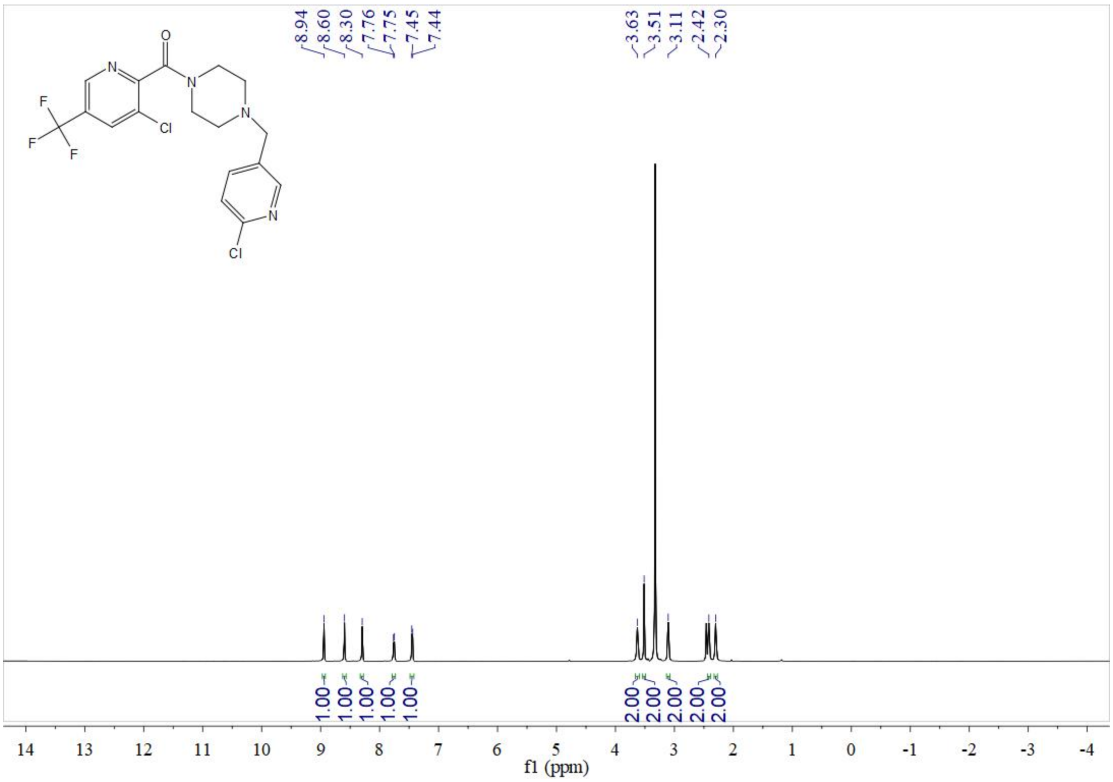

A24 ^{13}C NMR

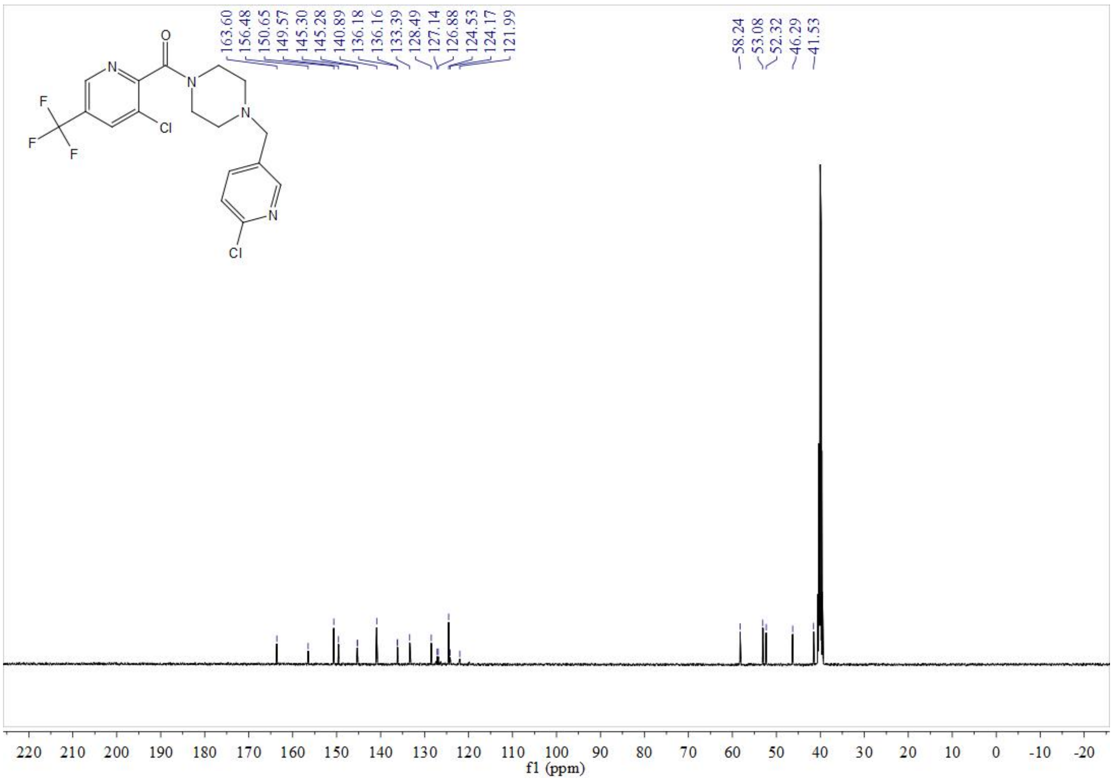

A24 ^{19}F NMR

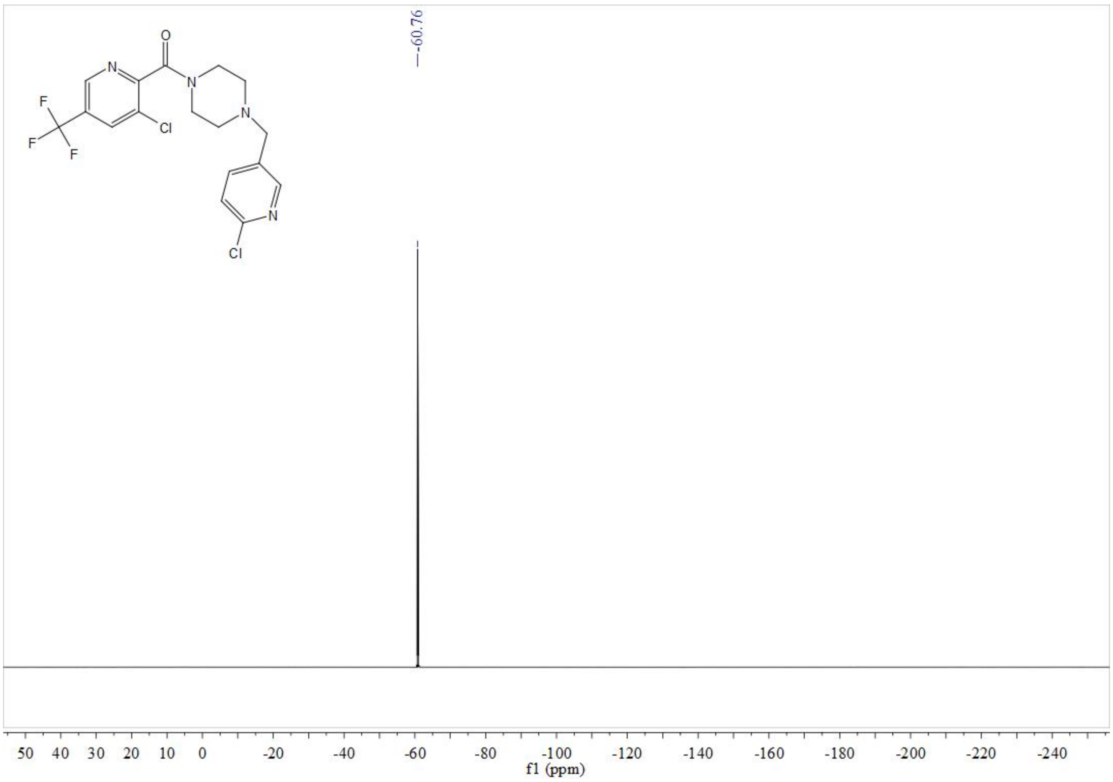

A24 HRMS

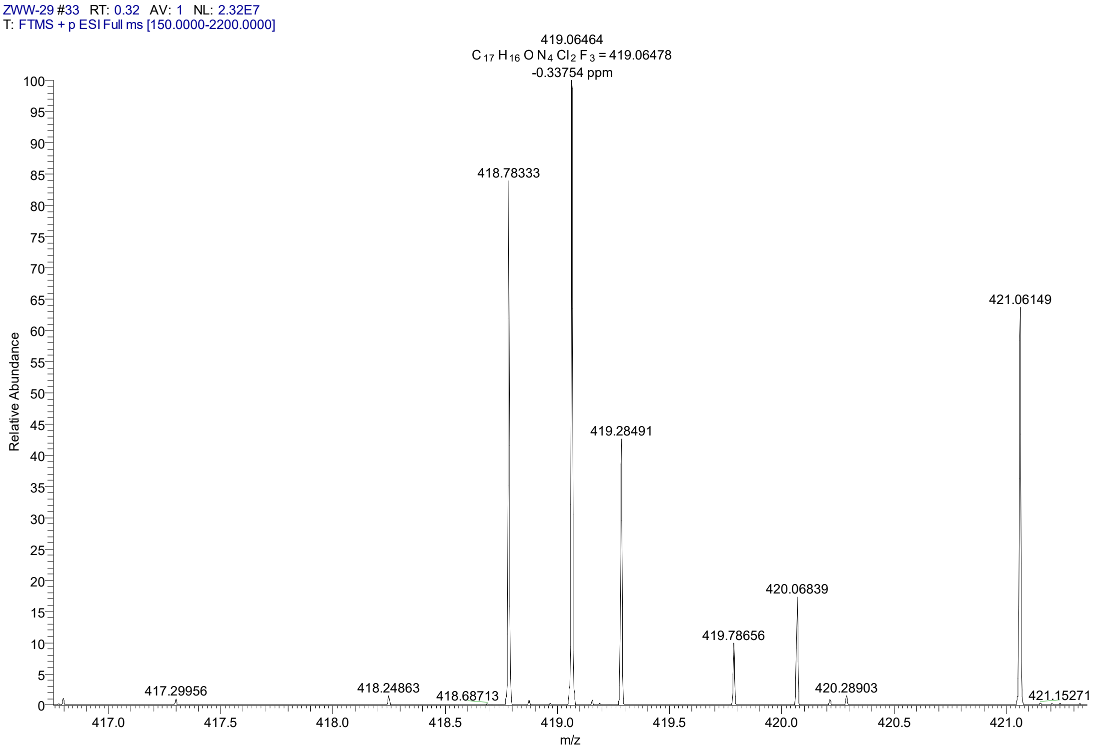

A25 ^{1}H NMR

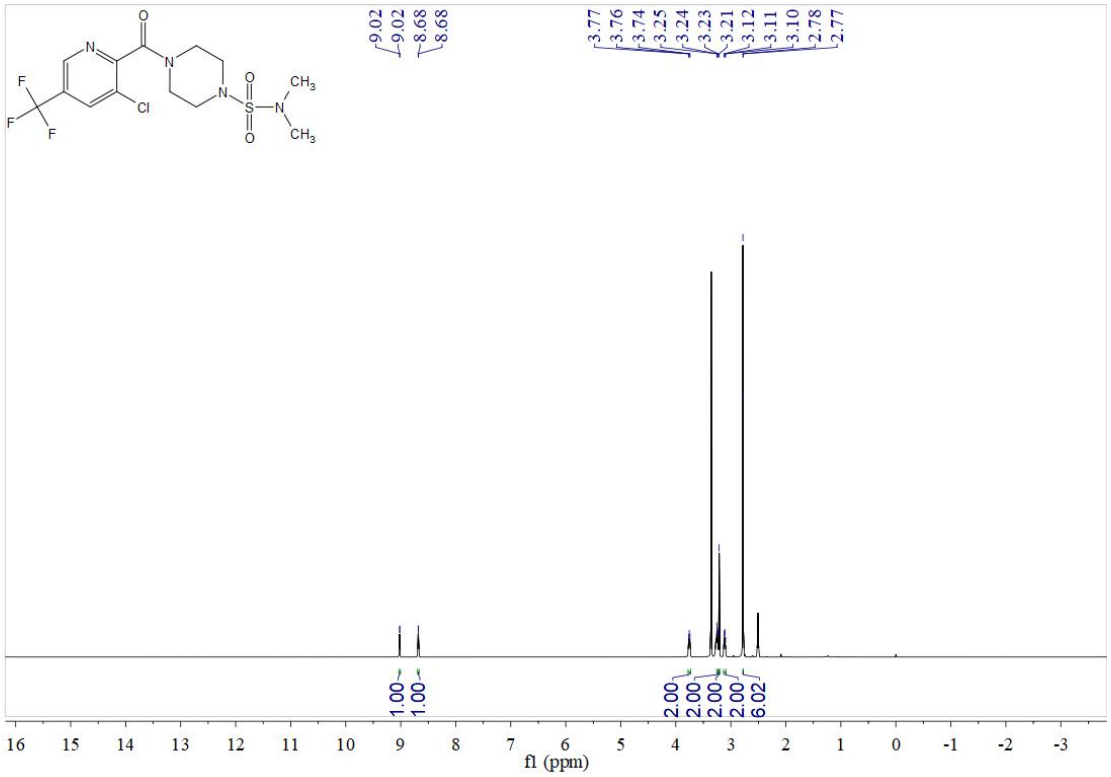

A25 ^{13}C NMR

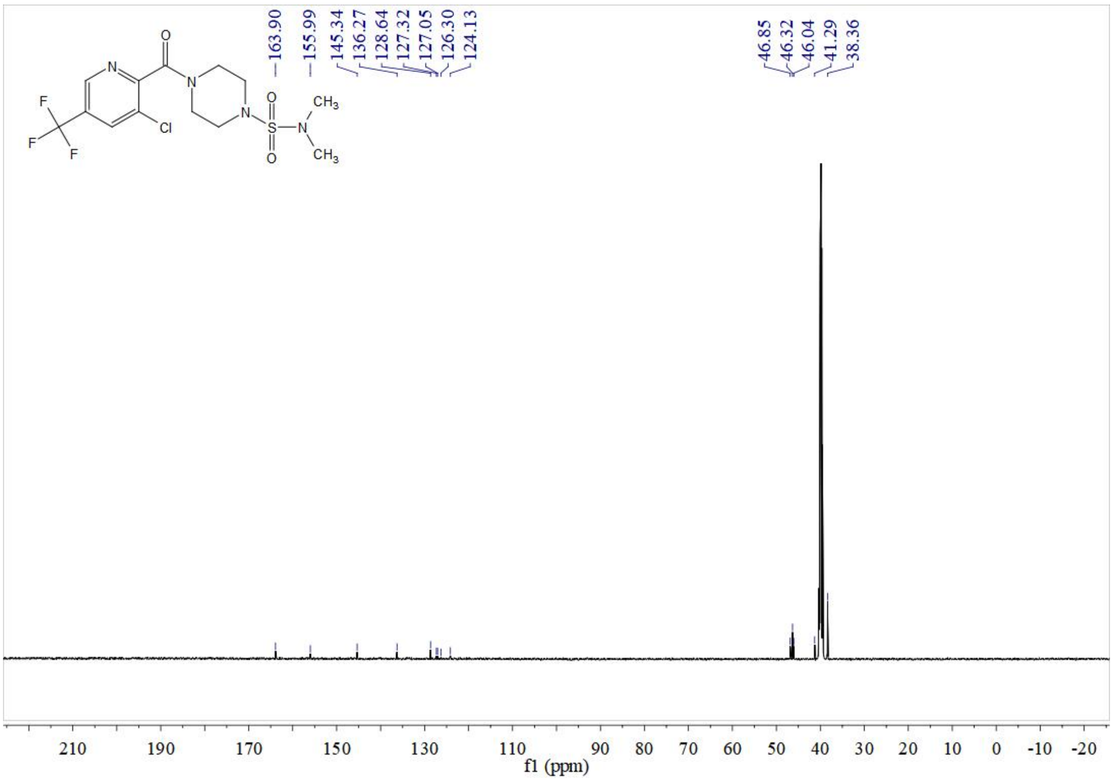

A25 ^{19}F NMR

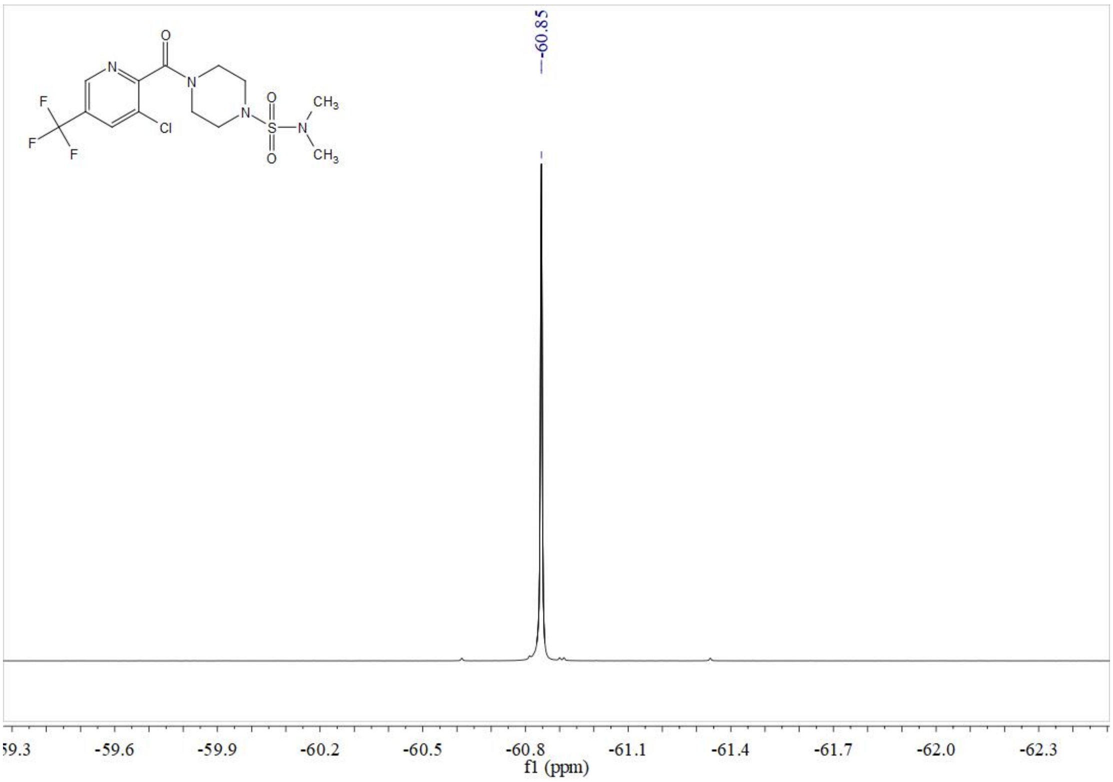

A25 HRMS

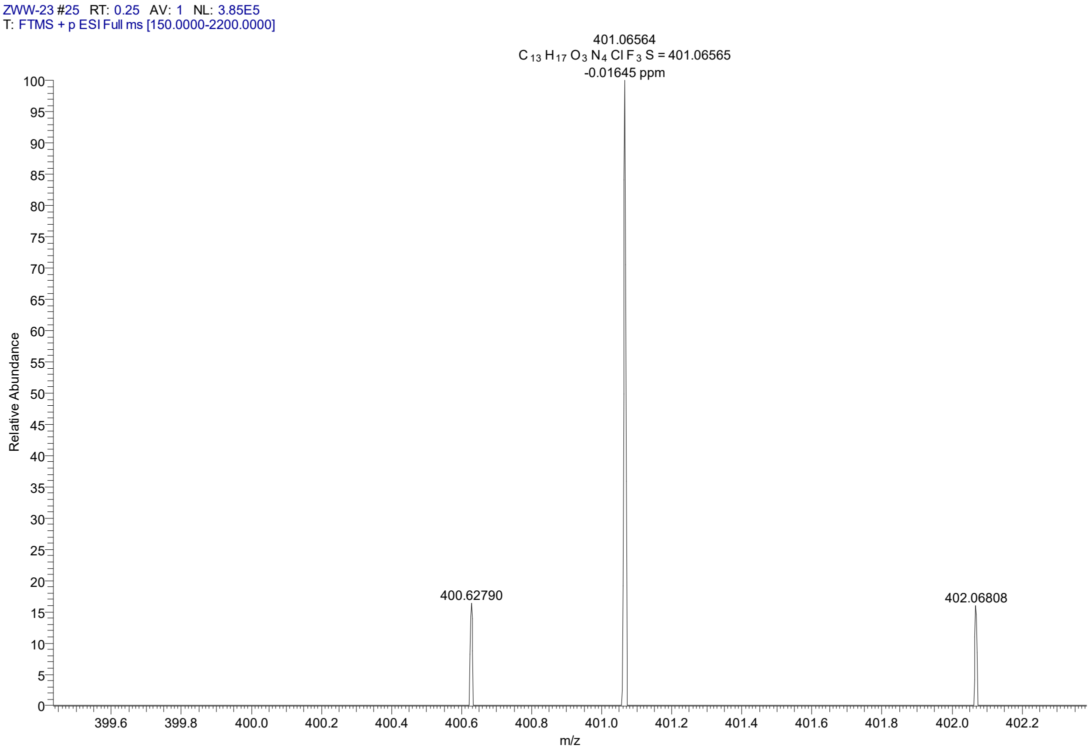

A26 ^{1}H NMR

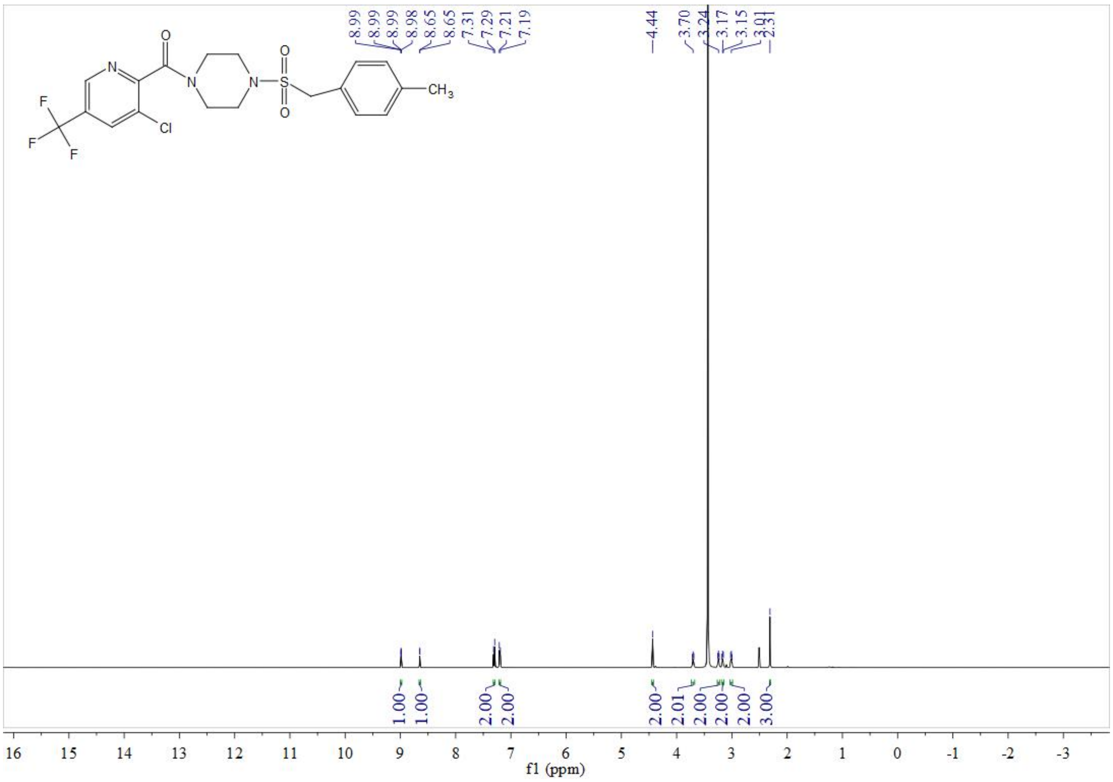

A26 ^{13}C NMR

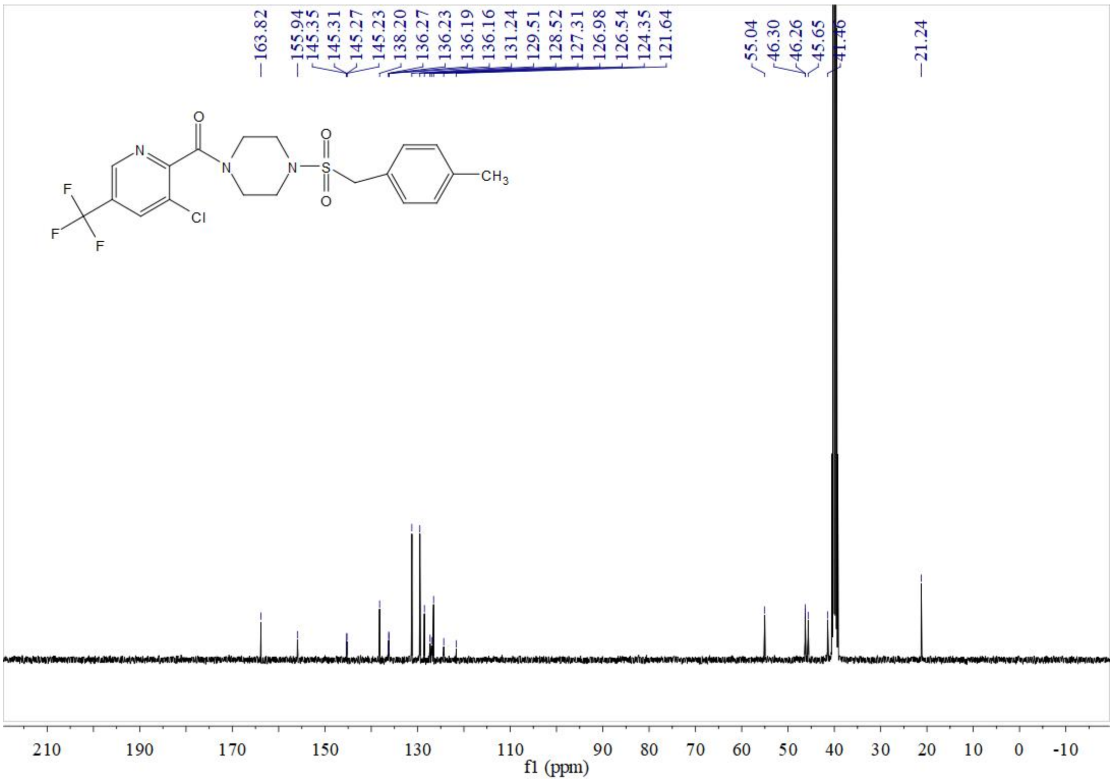

A26 ^{19}F NMR

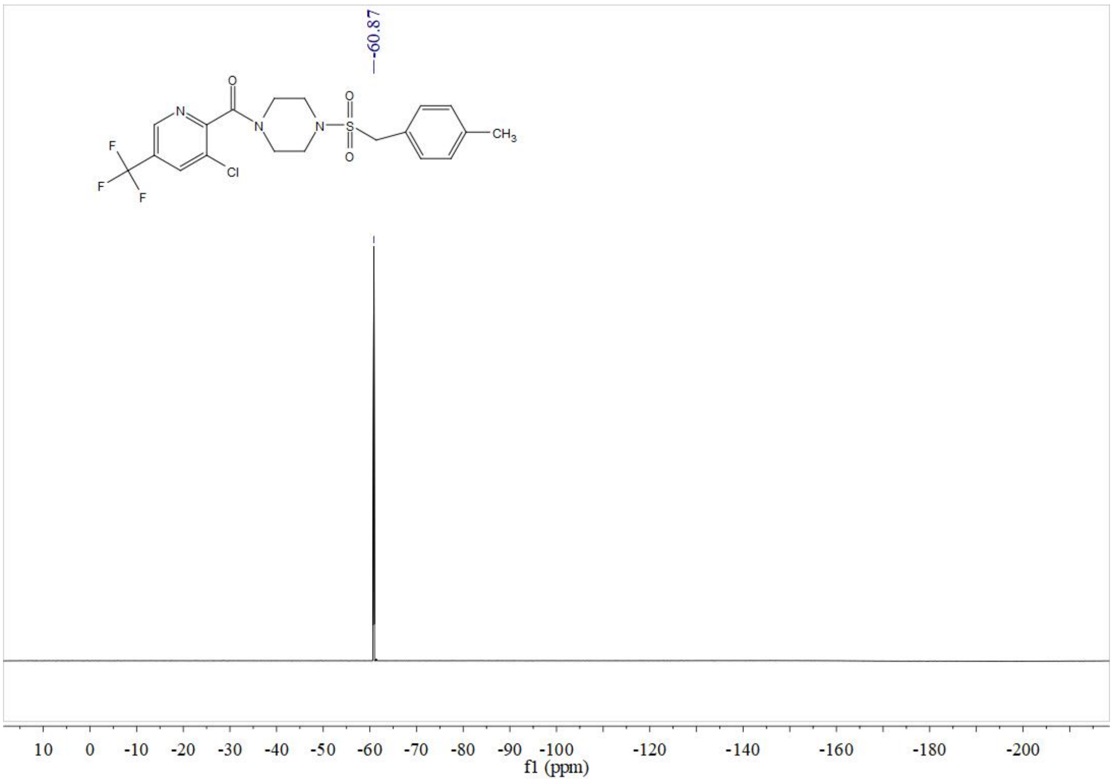

A26 HRMS

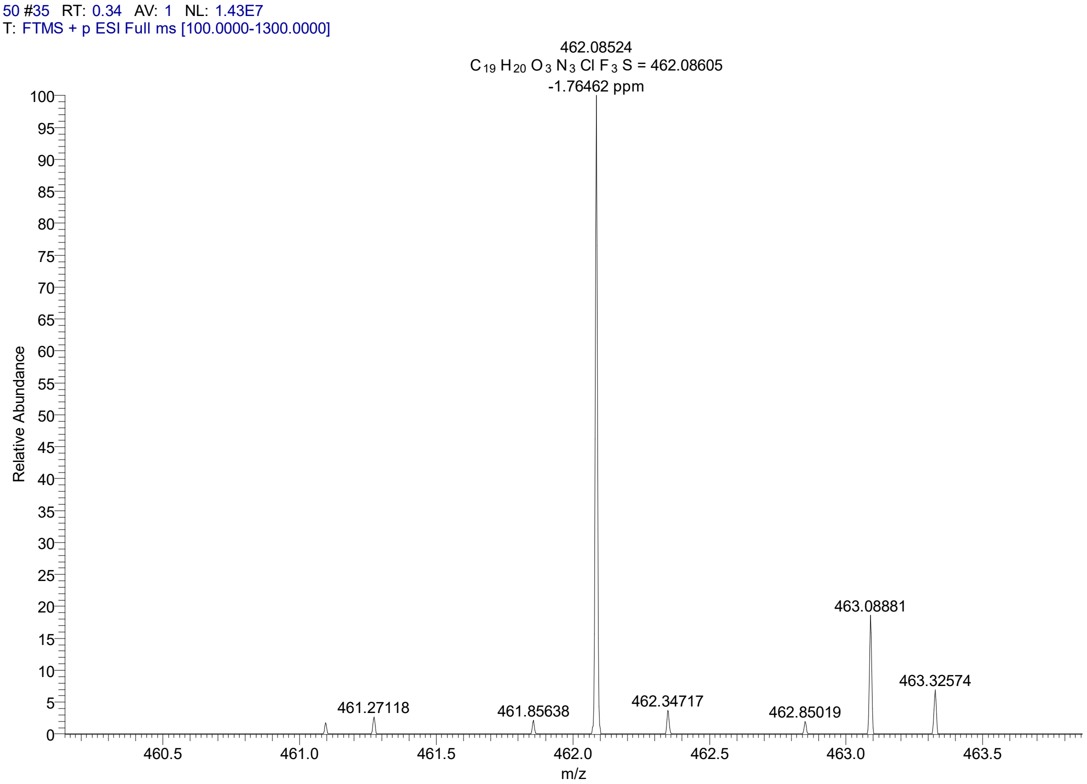

A27 ^{1}H NMR

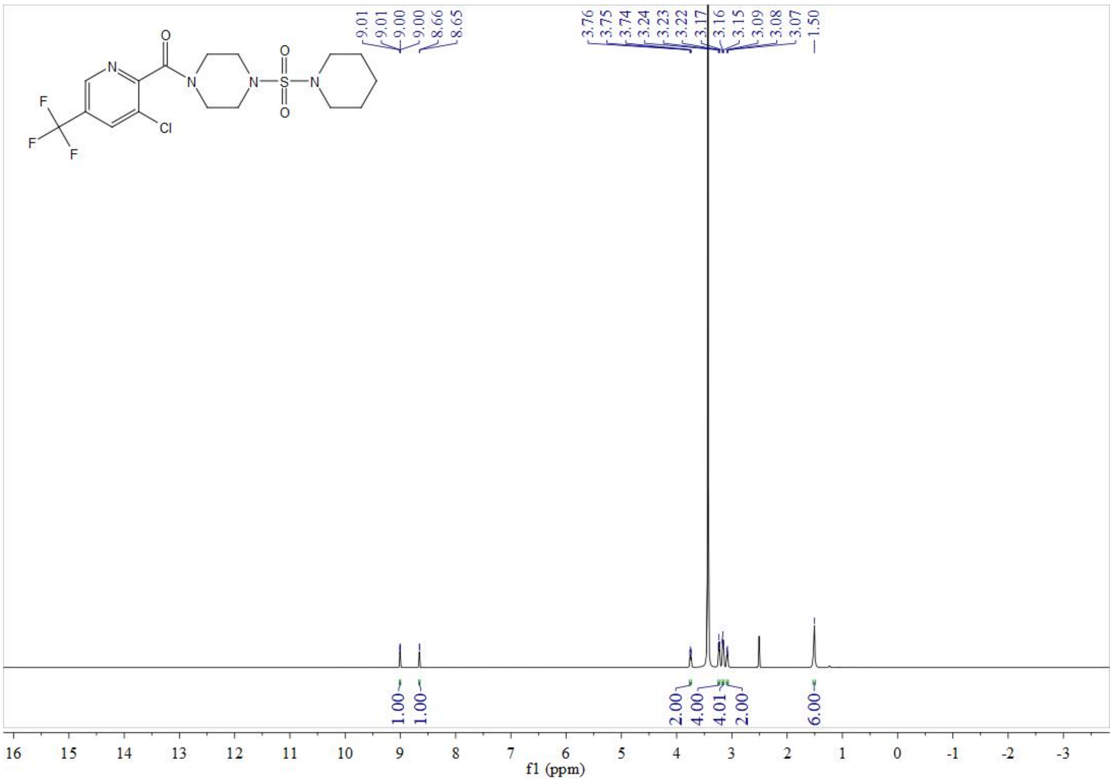

A27 ^{13}C NMR

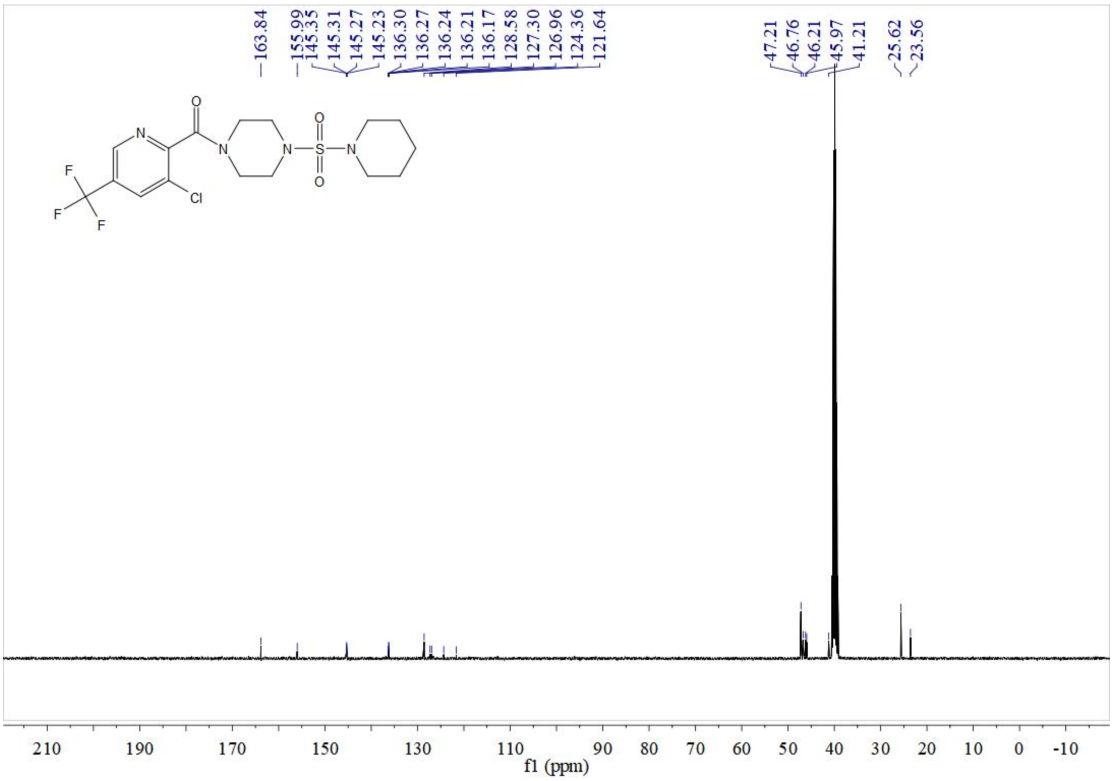

A27 ^{19}F NMR

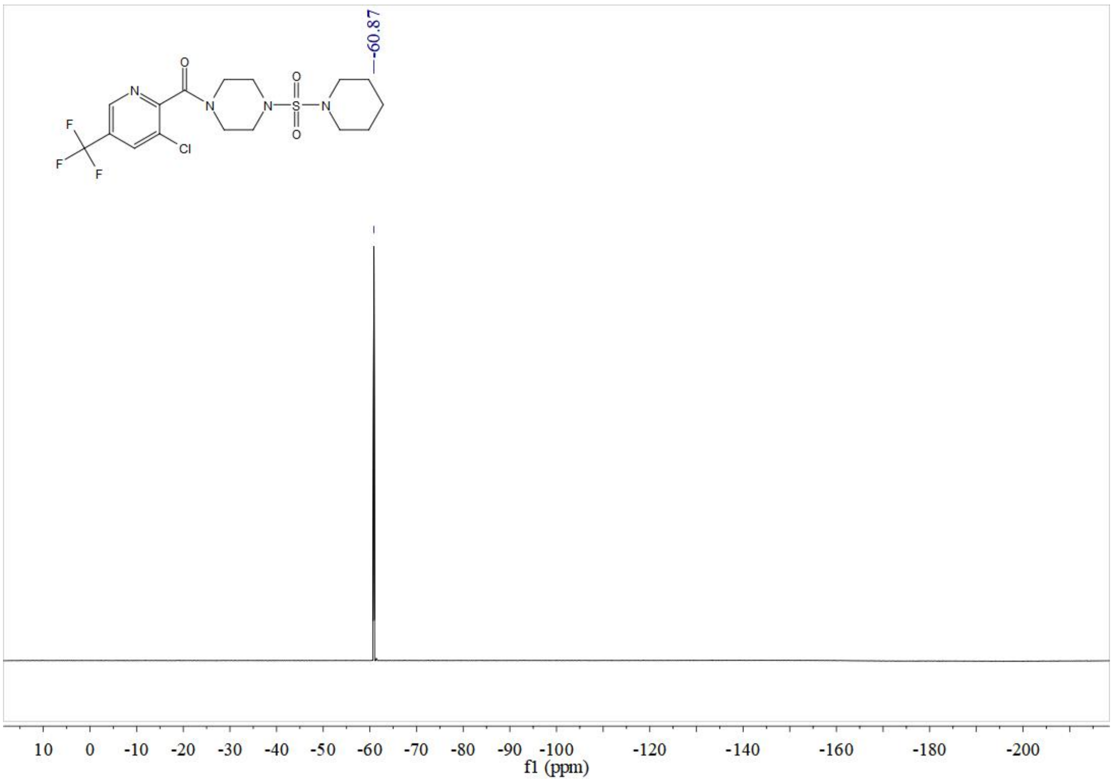

A27 HRMS

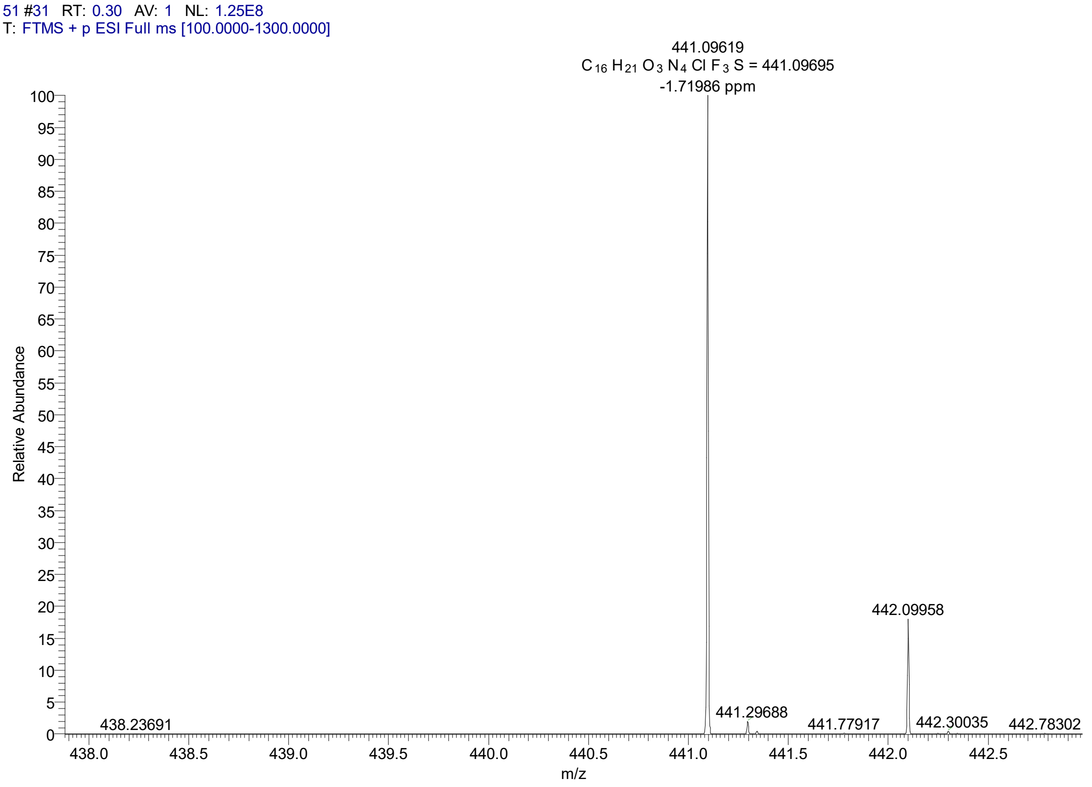

Supplement: Supplementary file 1 [file DataSheet_1.pdf]
